# Supplementary material for: Methodologies for studying depression in persons living with tuberculosis: Protocol for a scoping review
Source: PLoS One. 2025 Jul 21;20(7):e0322753. doi: 10.1371/journal.pone.0322753 (PMC12279151; doi:10.1371/journal.pone.0322753)
Supplement: Attachment — Submitted filename: PONE-D-25-16662_Mickael Essouma.pdf [file pone.0322753.s003.pdf]

PLOS ONE

Methodologies for Studying Depression in Persons living with Tuberculosis: Protocol for a Scoping Review

--Manuscript Draft--

**Manuscript Number:**	PONE-D-25-16622
**Article Type:**	Study Protocol
**Full Title:**	Methodologies for Studying Depression in Persons living with Tuberculosis: Protocol for a Scoping Review
**Short Title:**	Depression and TB: Protocol for Scoping Review
**Corresponding Author:**	Amanda J Gupta, Master of Public Health<br>Yale University<br>New Haven, Connecticut UNITED STATES OF AMERICA
**Keywords:**	Tuberculosis; Depression; global health; reviews
**Abstract:**	Background: Tuberculosis (TB) and depression frequently co-occur, yet research has largely focused on prevalence rather than diagnostic or treatment methodologies. Given overlapping symptoms, robust research approaches are critical for improving detection and treatment strategies. This scoping review aims to map methodologies used to study depression in individuals with TB, identifying gaps in research design, diagnosis, and treatment that may hinder clinical and public health advancements<br>Methods: A search will be conducted in MEDLINE, Embase, PsycINFO, Global Health, Cochrane Library, and Africa-Wide Information using controlled vocabulary related to TB and depression. Studies examining the TB-depression relationship will be included. We will use Covidence to facilitate screening, selection, and data extraction. We will extract data on study design, diagnostic tools, treatment interventions, and analytical approaches.<br>Results: Descriptive characteristics of included studies will be presented using figures and tables. Using the RE-AIM framework, we will evaluate reach by assessing the extent to which studies explore both directions of the TB-depression relationship and include diverse, high-risk populations. Effectiveness will be examined by categorizing study designs to assess methodological diversity and evaluating the diagnostic tools and treatment interventions used, along with their reported efficacy. Adoption will be analyzed by identifying where and by whom depression diagnosis and treatment methodologies have been implemented and whether research findings have influenced clinical guidelines or public health policies. Implementation will be assessed by identifying barriers and facilitators reported in qualitative studies or by researchers regarding the integration of depression care into TB treatment settings. Finally, maintenance will be determined by examining whether diagnostic and treatment methodologies were sustained within health systems, including long-term patient outcomes and the persistence of interventions beyond the research phase.<br>Conclusions: By mapping existing methodologies and identifying research gaps, this review will provide valuable insights to guide future research and improve diagnostic and treatment strategies.
**Order of Authors:**	Amanda J Gupta, Master of Public Health
	Patricia Turimumahoro
	Lori Rosman
	Jonathan E Golub
	David W Dowdy
**Opposed Reviewers:**	
**Additional Information:**	
**Question**	**Response**
**Financial Disclosure**	The author(s) received no specific funding for this work.

Powered by Editorial Manager® and ProduXion Manager® from Aries Systems Corporation

Enter a financial disclosure statement that describes the sources of funding for the work included in this submission. Review the submission guidelines for detailed requirements. View published research articles from *PLOS ONE* for specific examples.<br><br>This statement is required for submission and **will appear in the published article** if the submission is accepted. Please make sure it is accurate.<br><br>**Funded studies**<br>Enter a statement with the following details:<br>• Initials of the authors who received each award<br>• Grant numbers awarded to each author<br>• The full name of each funder<br>• URL of each funder website<br>• Did the sponsors or funders play any role in the study design, data collection and analysis, decision to publish, or preparation of the manuscript?<br><br>Did you receive funding for this work?	
**Competing Interests**<br><br>Use the instructions below to enter a competing interest statement for this submission. On behalf of all authors, disclose any competing interests that could be perceived to bias this work—acknowledging all financial support and any other relevant financial or non-financial competing interests.<br><br>This statement is required for submission and **will appear in the published article** if the submission is accepted. Please make sure it is accurate and that any funding sources listed in your Funding Information later in the submission form are also declared in your Financial Disclosure statement.<br><br>View published research articles from	The authors have declared that no competing interests exist

Powered by Editorial Manager® and ProduXion Manager® from Aries Systems Corporation

PLOS ONE for specific examples.<br><br>**NO authors have competing interests**<br><br>Enter: *The authors have declared that no competing interests exist.*<br><br>**Authors with competing interests**<br><br>Enter competing interest details beginning with this statement:<br><br>*I have read the journal's policy and the authors of this manuscript have the following competing interests: [insert competing interests here]*<br><br>* typeset	
**Ethics Statement**<br><br>Enter an ethics statement for this submission. This statement is required if the study involved:<br><br>• Human participants<br>• Human specimens or tissue<br>• Vertebrate animals or cephalopods<br>• Vertebrate embryos or tissues<br>• Field research<br><br>Write "N/A" if the submission does not require an ethics statement.<br><br>General guidance is provided below. Consult the submission guidelines for detailed instructions. **Make sure that all information entered here is included in the Methods section of the manuscript.**	N/A

Powered by Editorial Manager® and ProduXion Manager® from Aries Systems Corporation

**Format for specific study types**<br><br>**Human Subject Research (involving human participants and/or tissue)**<br>• Give the name of the institutional review board or ethics committee that approved the study<br>• Include the approval number and/or a statement indicating approval of this research<br>• Indicate the form of consent obtained (written/oral) or the reason that consent was not obtained (e.g. the data were analyzed anonymously)<br><br>**Animal Research (involving vertebrate animals, embryos or tissues)**<br>• Provide the name of the Institutional Animal Care and Use Committee (IACUC) or other relevant ethics board that reviewed the study protocol, and indicate whether they approved this research or granted a formal waiver of ethical approval<br>• Include an approval number if one was obtained<br>• If the study involved *non-human primates*, add *additional details* about animal welfare and steps taken to ameliorate suffering<br>• If anesthesia, euthanasia, or any kind of animal sacrifice is part of the study, include briefly which substances and/or methods were applied<br><br>**Field Research**<br><br>Include the following details if this study involves the collection of plant, animal, or other materials from a natural setting:<br>• Field permit number<br>• Name of the institution or relevant body that granted permission	
**Data Availability**<br><br>Authors are required to make the data underlying their research findings fully available, without restriction.<br><br>If the manuscript reports pilot or preliminary results, the data underlying those results must be made available at the time of publication. A Data Availability	N/A - No results are reported

Powered by Editorial Manager® and ProduXion Manager® from Aries Systems Corporation

Statement describing where the data can be found is required at submission. Your answers to this question constitute the Data Availability Statement and will be published in the article, if accepted. When legal or ethical requirements prohibit public data sharing, authors must indicate how researchers may obtain access to the data. See the PLOS Data Policy and FAQ for detailed information.<br><br>**Important:** Stating 'data available on request from the author' is not sufficient. If your data are only available upon request, select 'No' for the first question and explain your situation in the text box.<br><br>For protocols without pilot or preliminary data, authors are strongly encouraged to state how they plan to share research data from their study when it is completed or published.<br><br>If your study includes pilot data, will they be made fully available without restriction?	
**Describe where the data may be found in full sentences. If you are copying our sample text, replace any instances of XXX with the appropriate details.**<br><br>• If **no pilot data are reported** in the manuscript, please enter the following: *No datasets were generated or analysed during the current study. All relevant data from this study will be made available upon study completion.*<br>• If the manuscript reports pilot data **held in a public repository**, include URLs, accession numbers or DOIs. If this information will only be available after acceptance, indicate this by ticking the box below. For example: *All XXX files are available from the XXX database (accession number(s) XXX, XXX.).*<br>• If the manuscript reports pilot data contained **within the manuscript and/or**	No datasets were generated or analysed during the current study. All relevant data from this study will be made available upon study completion.

Powered by Editorial Manager® and ProduXion Manager® from Aries Systems Corporation

**Supporting Information files**, enter the following:<br>*All relevant data are within the manuscript and its Supporting Information files.*<br>• If neither of these applies but you are able to provide **details of access elsewhere**, with or without limitations, please do so. For example:<br><br>*Data cannot be shared publicly because of [XXX]. Data are available from the XXX Institutional Data Access / Ethics Committee (contact via XXX) for researchers who meet the criteria for access to confidential data.*<br><br>*The data underlying the results presented in the study are available from (include the name of the third party and contact information or URL).*<br>• This text is appropriate if the data are owned by a third party and authors do not have permission to share the data.<br><br>For study protocols with data management or sharing plans, authors are encouraged to briefly describe in their Data Availability Statement how data they generate will be made accessible when the study is completed. For example:<br>• Deidentified research data will be made publicly available when the study is completed and published.<br><br>* typeset	
Additional data availability information:	

Powered by Editorial Manager® and ProduXion Manager® from Aries Systems Corporation

Amanda J Gupta
Yale University
School of Public Health
amanda.meyer@yale.edu
March 25, 2025

Dear Academic Editors,

I am pleased to submit our protocol manuscript titled “Methodologies for Studying Depression in Persons living with Tuberculosis: Protocol for a Scoping Review” for consideration for publication in PLOS ONE. This manuscript presents a scoping review protocol that aims to systematically map the methodologies employed in studying the intersection of tuberculosis (TB) and depression. Given the high prevalence of depression among persons with tuberculosis and diagnostic challenges posed by overlapping symptomatology, our study contributes to the scientific literature by identifying methodological gaps that may limit the development of effective diagnostic tools and treatment strategies.

This work is a protocol for a scoping review and is designed to provide a comprehensive overview of current research approaches. Our protocol employs the Levac et al framework, and incorporates the RE-AIM framework to systematically evaluate reach, effectiveness, adoption, implementation, and maintenance of methodologies reported in the literature. This approach will highlight opportunities to optimize research and public health interventions, ultimately improving outcomes for individuals with TB and depression.

We believe that this protocol will be of particular interest to readers and researchers in global health, epidemiology, mental health, and public health policy, as it offers valuable insights into the current state of research and highlights areas in need of methodological advancement. The findings from our scoping review are expected to guide future studies by providing a roadmap for integrating validated diagnostic measures and specialized treatment approaches into routine TB care.

To handle our manuscript, we suggest that Academic Editors with expertise in systematic reviews, global health, and mental health be considered. We recommend:

- Dr. Divya Bhandari (expertise in systematic reviews and global health)
- Dr. Esther Buregyeya (expertise in tuberculosis and epidemiology)
- Dr. Abel Fekadu Dadi, (expertise in depression, global health, and reviews)

Thank you for considering our protocol for publication in PLOS ONE. We look forward to your feedback and the opportunity to contribute to the scientific discourse in this important area.

Sincerely,

Amanda J Gupta
Research Associate II
Yale School of Public Health

Manuscript

Click here to access/download;Manuscript;Gupta et al_TB and depression Scoping Review Protocol.docx

Title: Methodologies for Studying Depression in Persons living with Tuberculosis: Protocol for a Scoping Review

Authors: Amanda J Gupta[1,2], Patricia Turimumahoro[3], Lori Rosman[4], Jonathan E. Golub[5-7], David W Dowdy[5,6]

1. Epidemiology of Microbial Diseases, Yale School of Public Health, New Haven, CT, USA
2. Johns Hopkins Bloomberg School of Public Health, Maryland, USA
3. WALIMU, Kampala, Uganda
4. Welch Medical Library, School of Medicine, Johns Hopkins University, Baltimore, MD, USA
5. Department of Epidemiology, Johns Hopkins Bloomberg School of Public Health, Baltimore, Maryland, USA
6. Department of International Health, Johns Hopkins Bloomberg School of Public Health, Baltimore, Maryland, USA
7. Center for Tuberculosis Research, Johns Hopkins University School of Medicine, Baltimore, Maryland, USA

Corresponding Author: Amanda J Gupta,

60 College Street, New Haven, CT 06510

amanda.meyer@yale.edu

Keywords: tuberculosis; depression; global health; reviews

Abstract (296 words):

Background: Tuberculosis (TB) and depression frequently co-occur, yet research has largely focused on prevalence rather than diagnostic or treatment methodologies. Given overlapping symptoms, robust research approaches are critical for improving detection and treatment strategies. This scoping review aims to map methodologies used to study depression in individuals with TB, identifying gaps in research design, diagnosis, and treatment that may hinder clinical and public health advancements

Methods: A search will be conducted in MEDLINE, Embase, PsycINFO, Global Health, Cochrane Library, and Africa-Wide Information using controlled vocabulary related to TB and depression. Studies examining the TB-depression relationship will be included. We will use Covidence to facilitate screening, selection, and data extraction. We will extract data on study design, diagnostic tools, treatment interventions, and analytical approaches.

Results: Descriptive characteristics of included studies will be presented using figures and tables. Using the RE-AIM framework, we will evaluate reach by assessing the extent to which studies explore both directions of the TB-depression relationship and include diverse, high-risk populations. Effectiveness will be examined by categorizing study designs to assess methodological diversity and evaluating the diagnostic tools and treatment interventions used, along with their reported efficacy. Adoption will be analyzed by identifying where and by whom depression diagnosis and treatment methodologies have been implemented and whether research findings have influenced clinical guidelines or public health policies. Implementation will be assessed by identifying barriers and facilitators reported in qualitative studies or by researchers regarding the integration of depression care into TB treatment settings.

Finally, maintenance will be determined by examining whether diagnostic and treatment methodologies were sustained within health systems, including long-term patient outcomes and the persistence of interventions beyond the research phase.

Conclusions: By mapping existing methodologies and identifying research gaps, this review will provide valuable insights to guide future research and improve diagnostic and treatment strategies.

Introduction

Tuberculosis (TB) is one of the leading infectious killers worldwide. The COVID-19 pandemic accelerated these deaths as less attention, and subsequently funding, has been paid to TB [1, 2]. This resulted in an estimated 10.8 million people developing new cases of active TB in 2023 with an estimated 1.25 million deaths due to TB disease [2]. Despite this large burden, TB is vastly underfunded leading to under-supported research endeavors and national TB programs left without appropriate funding to successfully combat the large TB burdens that they may face [1-5]. This results in gaps in TB diagnosis, treatment, and care in high TB burden settings [6-8].

One such gap in care is in the screening, diagnosis, and treatment of comorbidities in people with TB (PWTB) [9]. While certain communicable comorbidities (such as TB diagnosis and treatment in those living with HIV [10-13]) have been extensively researched, non-communicable comorbidities – and especially mental health comorbidities – have received little attention despite their impacts on successful TB treatment [9, 14, 15]. Depression is ~~one such~~ overlooked ~~comorbidity~~ with potentially large impacts on PWTB's long-term health and wellbeing.

Depression appears to impact PWTB more frequently than the general population with one systematic review estimating the prevalence of depression amongst PWTB to be a staggering 45% (16). In fact, depression may even be a risk factor for developing TB [17]. However, most PWTB are never screened for depression, let alone receive treatment for their depression, due to a lack of providers, funding, cultural norms, and

stigma [16, 18-20]. Without treatment, depression increases the chances that a PWTB delays seeking and initiating treatment [18, 21], is non-adherent to TB medications once they are diagnosed [22, 23], and ultimately increases their chances for negative TB outcomes such as loss to follow up and death [24, 25].

Despite the large burden that depression poses for both health systems and PWTB, research appears to have been limited to prevalence surveys with few studies using longitudinal designs to investigate the intersection of these two diseases and even fewer exploring the methodologies used to screen, diagnose, or treat depression in PWTB. This may be in part due to diagnostic challenges posed by overlapping symptoms, such as TB-related fatigue, weight loss, and general malaise, all of which can masquerade as signs of depression, especially at the point of TB diagnosis, leading to misdiagnoses or missed diagnoses. Therefore, we aim to conduct a scoping review to examine the methodologies used to study depression, including diagnostic and treatment methodologies, in PWTB, identifying research gaps and highlighting opportunities for improved methodological approaches in future public health research.

~~Methods~~

We will utilize the framework for conducting scoping studies originally developed by Arskey and O'Malley [26] and updated by Levac et al [27]. This framework ~~consists~~ of six steps ~~consisting of~~ 1) Identifying the research question 2) Identifying relevant studies 3) Study Selection 4) Charting the data 5) Collating, summarizing, and reporting the

results and 6) Consultation with stakeholders on study findings.

Identifying the Research Question

The research question for our review is: what methodologies have been used in the research literature to study depression (including diagnosis and treatment) among individuals with tuberculosis, and what are the gaps in methodological approaches? We will screen studies that have sought to examine the relationship between TB and depression.

Identification of Relevant Studies

Table 1 includes the eligibility criteria for this scoping review. We will search MEDLINE, Embase, The WHO Regional Libraries, Global Health, and The Cochrane Library due to their near-ubiquitous use for biomedical and healthcare research along with Africa-Wide Information due to the high burden of TB in Africa, and PsychInfo for its specialization in psychology. Given the anticipated paucity of literature, we will not include date restrictions but rather allow any study to be included that was published prior to the search date. Finally, reference lists of included studies will be searched to ensure no potentially relevant works have been overlooked.

Table 1. Study Eligibility Criteria

Domain	Included	Excluded
Participants	Individuals with tuberculosis regardless of type (microbiologically confirmed or clinically diagnosed; pulmonary or extra-pulmonary, latent or active, drug-resistant or drug-susceptible)	Those without a confirmed tuberculosis diagnosis (for example those with symptoms of tuberculosis rather than a diagnosis), studies involving animals, studies involving nontuberculous mycobacterial infections (such as leprosy), and studies including only laboratory samples and isolates

Concept or intervention	Studies that contain information on depression and tuberculosis including diagnostic tools, methods, and protocols, and therapeutic interventions (pharmacological or psychological)	Studies that only include tuberculosis or depression independently without exploring their co-occurrence, or those that only focus on biological mechanisms of tuberculosis and/or depression
Outcome	Sensitivity, specificity, and/or accuracy of diagnostic tools for depression in TB patients, and studies focused on the identification of diagnostic challenges, barriers, or facilitators and validation of relevant diagnostic measures. Changes in depression symptoms (including patient-reported outcomes), TB treatment adherence, completion, and/or complication rates because of depression treatment or lack thereof. Studies with outcomes on methodological challenges in studying depression and tuberculosis together will be eligible.	Outcomes not explicitly linked to the study of depression in TB populations including studies reporting biochemical, genetic, or molecular outcomes without clinical or diagnostic relevance, those with general TB treatment outcomes unrelated to or without considering depression, those with insufficient detail on how outcomes were measured or analyzed, and those based solely on opinions or informal observations
Context	Research conducted in healthcare or community settings of relevance such as tuberculosis clinics, hospital settings, primary care clinics, community health programs and any other relevant setting where tuberculosis and depression services may be provided. Global geographic inclusion	Research from unrelated contexts such as veterinary or laboratory studies. No exclusion criteria based on geography
Language	English	Language other than English
Study Design	Primary studies, randomized controlled trials, prevalence surveys, case studies, cohort studies, case-control studies, systematic reviews, meta-analyses, scoping reviews, qualitative studies, mixed methods analyses, diagnostic test accuracy studies	Meta-reviews (reviews of reviews), opinion pieces, editorials, letters to the editor that do not contain empirical data, and abstracts without full-text availability

The preliminary search will focus on combinations of the following areas: terms related to “tuberculosis” and terms related to “depression”. Appropriate MeSH terms will be utilized for each of the proposed databases (S1 Appendix).

Study Selection

We will use Covidence to screen study titles, abstracts and full text articles for inclusion. Two reviewers (AJG, PT) will review abstracts in parallel for inclusion. To minimize bias, reviewers will be blinded to other reviewers' ratings for screening except when resolving conflicts. Reviewers will meet after every 20 abstracts to resolve any conflicts and to propose/implement updates to the search strategy or inclusion criteria. All abstracts that pass screening will have the full text reviewed by the two reviewers, again, in parallel for final inclusion in the review. Disagreements will be resolved via discussion and, if needed, by a third reviewer (DWD).

Charting the Data

All data will be extracted using Covidence. We will extract pertinent study details such as authors, location of publication/journal name, year of publication, research question addressed, study design, geographic context, date when study was conducted, study location (community, health facility, etc.), sample size, participants characteristics (such as age, HIV status, sex, other mental health disorders, etc.), screening and diagnostic tools used for depression, treatment intervention approach utilized for depression (e.g. psychological approaches like cognitive-behavioral therapy, pharmacological like antidepressants, and/or alternative interventions like mindfulness techniques or yoga), treatment intervention components outcomes assessed, treatment intervention mode of delivery (e.g. in-person therapy, oral medication etc.), duration and frequency of treatment intervention, statistical methods used (e.g. regression analysis, chi-square, multi-level models etc.), comparison population (if a control or other comparator used), outcome measures (e.g, prevalence estimates, odds ratios etc.), measures of precision

(e.g. 95% CI), statistical significance, and, for qualitative studies, key themes identified, barriers and facilitators to diagnosis and/or treatment identified, and the framework or content analysis utilized. Additionally, if the authors identify any limitations relevant to the research question, such as methodological limitations, we will extract this data. One author (AJG) will extract the data with a second reviewer (PT) checking extracted data for accuracy. These two authors will routinely meet during the data extraction phase to ensure the extraction tool is capturing all relevant data needed to answer the overall research question.

~~Results~~

Collating, Summarizing, and Reporting Results

We will provide descriptive characteristics of all studies included in the review using figures and tables to fully summarize the studies. While our focus in the present research is to characterize the current state of the literature with inclusion not dependent on literature quality, we will conduct a quality appraisal of each study as the presence or absence of high-quality research is relevant to understanding research gaps.

We will structure the reporting of our results using the RE-AIM (Reach, Effectiveness, Adoption, Implementation and Maintenance) framework [28]. As we are interested in the way these two diseases have been studied together, for *reach*, we will determine the extent to which studies have explored both directions of the TB-depression relationship. We will also determine the reach of researchers in terms of including representative

samples of PWTB, including diverse populations and healthcare settings, and their inclusion of high-risk groups (such as those with HIV, drug-resistant TB etc.). For *effectiveness* we will categorize the study designs used to assess the diversity of methodological approaches as a wide range of designs – such as cross-sectional, longitudinal and randomized controlled trials – is essential for fully elucidating the co-occurrence of TB and depression. A lack of diverse study designs may indicate gaps in the field's ability to fully understand and address this relationship. Additionally, we will determine the types of diagnostic measures used for depression along with their effectiveness (if reported) as well as how efficacious or effective any treatment interventions were at altering either depression or TB related outcomes. For *adoption* we will look at the extent to which depression screening, diagnosis, and treatment methodologies are being adopted in different settings (such as by national programs, referral centers, local clinics, etc) and by whom (e.g. physicians, nurses, community health works etc.) and examine the way in which research findings on these two diseases have impacted clinical guidelines and public health policies if mentioned by study authors. For *implementation*, we will determine barriers and facilitators identified to the implementation of depression related care in real-world TB treatment settings. Finally, for *maintenance* we will determine whether any depression related methodologies for diagnosis or treatment were sustained or embedded within existing health systems. This will include, if reported upon, long-term patient outcomes, if methodologies persisted beyond the research phase, and if any studies evaluated how health systems integrated and sustained any proposed diagnostic or treatment interventions.

Timeline

The initial search was conducted on March 31, 2025. All screening, including title and abstract screening and full text screening will take place from April to May 2025. Data extraction will take place in July 2025. Analysis and synthesis of results will take place from July to August 2025 with final results expected by September 2025.

Discussion

This scoping review will explore the existing literature on the intersection between depression and TB allowing us to identify research gaps, including in the diagnosis and treatment of depression, amongst PWTB. Importantly, this will allow us to understand any methodological gaps that exist and approaches that researchers have historically taken to tackle these two diseases concurrently.

By mapping the existing methodologies used to study depression in PWTB, this review will identify gaps in research design, highlight areas where more rigorous and diverse study approaches are needed, and provide insights into potential pathways for improving integrated care. Findings from this review will contribute to advancing research beyond cross-sectional prevalence surveys by encouraging the use of longitudinal, interventional, and mixed-methods studies that can better inform evidence-based practice. Furthermore, this review will underscore the urgent need for validated diagnostic tools and specialized treatment approaches to address the overlapping symptoms and complex interplay between tuberculosis and depression, ultimately

supporting the development of more effective strategies to guide future research and public health interventions.

Our review does have some limitations. First, we have limited the search to English language studies. Given the burden of tuberculosis in many non-English speaking countries such as China where many publications are in Mandarin, this may exclude several studies that would otherwise be eligible for this scoping review. Second, we are beginning this search with the term depression, a Western term, and its associated synonyms. Many countries use alternate terms for depression, such as shenjing shuairuo (nervous weakness) in China or yuutsu in Japan, meaning that our initial search may not fully capture the available literature. Given the long list of terms that could possibly be used by different countries, it is not likely we will be able to capture all possible terms and subsequently may miss eligible publications.

Conclusions

This scoping review will further our knowledge of the extent of the literature concerning depression amongst those with TB by identifying research gaps for future researchers to fill.

Acknowledgements

We would like to thank both Yale University and Johns Hopkins University for providing institutional support for this work.

Author Contributions

AJG conceptualized the research question and wrote the initial draft of this protocol. PT, JEG, and DWD provided substantial contributions to the conception and design of the protocol including reviewing it for intellectual merit and refinement of the research question. LR created the initial search queries and ran the initial search. All authors reviewed and approved the final manuscript.

References

1. World Health Organization. Global Tuberculosis Report 2022. Geneva, Switzerland: World Health Organization; 2022.
2. World Health Organization. Global Tuberculosis Report 2024. Geneva, Switzerland; World Health Organization; 2024. Available from: https://www.who.int/teams/global-programme-on-tuberculosis-and-lung-health/tb-reports/global-tuberculosis-report-2024
3. Chaisson RE, Frick M, Nahid P. The scientific response to TB - the other deadly global health emergency. Int J Tuberc Lung Dis. 2022;26(3):186-9.
4. Venkatesan P. Worrying lack of funding for tuberculosis. Lancet Infect Dis. 2022;22(3):318.
5. Treatment Action Group. Tuberculosis Research Funding Trends 2005-2023. New York, New York; 2024 13 December, 2024. Available from: https://www.treatmentactiongroup.org/resources/tbrd-report/tbrd-report-2024/
6. Armstrong-Hough M, Turimumahoro P, Meyer AJ, Ochom E, Babirye D, Ayakaka I, et al. Drop-out from the tuberculosis contact investigation cascade in a routine public health setting in urban Uganda: A prospective, multi-center study. PLoS One. 2017;12(11):e0187145.
7. Kim J, Keshavjee S, Atun R. Health systems performance in managing tuberculosis: analysis of tuberculosis care cascades among high-burden and non-high-burden countries. J Glob Health. 2019;9(1):010423.
8. Subbaraman R, Jhaveri T, Nathavitharana RR. Closing gaps in the tuberculosis care cascade: an action-oriented research agenda. Journal of Clinical Tuberculosis and Other Mycobacterial Diseases. 2020;19:100144.

9. Jarde A, Romano E, Afaq S, Elsony A, Lin Y, Huque R, et al. Prevalence and risks of tuberculosis multimorbidity in low-income and middle-income countries: a meta-review. BMJ Open. 2022;12(9):e060906.

10. Duarte R, Lönnroth K, Carvalho C, Lima F, Carvalho ACC, Muñoz-Torrico M, et al. Tuberculosis, social determinants and co-morbidities (including HIV). Pulmonology. 2018;24(2):115-9.

11. Bell LCK, Noursadeghi M. Pathogenesis of HIV-1 and Mycobacterium tuberculosis co-infection. Nat Rev Microbiol. 2018;16(2):80-90.

12. Méndez-Samperio P. Diagnosis of Tuberculosis in HIV Co-infected Individuals: Current Status, Challenges and Opportunities for the Future. Scand J Immunol. 2017;86(2):76-82.

13. Dorman SE. Diagnosis of HIV-associated tuberculosis. Curr Opin HIV AIDS. 2018;13(6):462-8.

14. Galea JT, Monedero-Recuero I, Sweetland AC. Beyond screening: a call for the routine integration of mental health care with tuberculosis treatment. Public Health Action. 2019;9(1):2.

15. Cáceres G, Calderon R, Ugarte-Gil C. Tuberculosis and comorbidities: treatment challenges in patients with comorbid diabetes mellitus and depression. Therapeutic Advances in Infectious Disease. 2022;9:20499361221095831.

16. Duko B, Bedaso A, Ayano G. The prevalence of depression among patients with tuberculosis: a systematic review and meta-analysis. Annals of General Psychiatry. 2020;19(1):30.

17. Cheng KC, Liao KF, Lin CL, Lai SW. Increased Risk of Pulmonary Tuberculosis in Patients with Depression: A Cohort Study in Taiwan. Front Psychiatry. 2017;8:235.

18. Dos Santos AP, Lazzari TK, Silva DR. Health-Related Quality of Life, Depression and Anxiety in Hospitalized Patients with Tuberculosis. Tuberc Respir Dis (Seoul). 2017;80(1):69-76.

19. Hayward SE, Deal A, Rustage K, Nellums LB, Sweetland AC, Boccia D, et al. The relationship between mental health and risk of active tuberculosis: a systematic review. BMJ Open. 2022;12(1):e048945.

20. Rathod S, Pinninti N, Irfan M, Gorczynski P, Rathod P, Gega L, et al. Mental Health Service Provision in Low- and Middle-Income Countries. Health Services Insights. 2017;10:1178632917694350.

21. Molebatsi K, Wang Q, Dima M, Ho-Foster A, Modongo C, Zetola N, et al. Depression and delayed tuberculosis treatment initiation among newly diagnosed patients in Botswana. Global Public Health. 2021;16(7):1088-98.

22. Theron G, Peter J, Zijenah L, Chanda D, Mangu C, Clowes P, et al. Psychological distress and its relationship with non-adherence to TB treatment: a multicentre study. BMC Infect Dis. 2015;15:253.

23. Yan S, Zhang S, Tong Y, Yin X, Lu Z, Gong Y. Nonadherence to Antituberculosis Medications: The Impact of Stigma and Depressive Symptoms. Am J Trop Med Hyg. 2018;98(1):262-5.

24. Ruiz-Grosso P, Cachay R, de la Flor A, Schwalb A, Ugarte-Gil C. Association between tuberculosis and depression on negative outcomes of tuberculosis treatment: A systematic review and meta-analysis. PLOS ONE. 2020;15(1):e0227472.

25. Ambaw F, Mayston R, Hanlon C, Medhin G, Alem A. Untreated depression and tuberculosis treatment outcomes, quality of life and disability, Ethiopia. Bull World Health Organ. 2018;96(4):243-55.

26. Arksey H, O'Malley L. Scoping studies: towards a methodological framework. International Journal of Social Research Methodology. 2005;8(1):19-32.

27. Levac D, Colquhoun H, O'Brien KK. Scoping studies: advancing the methodology. Implement Sci. 2010;5:69.

28. Glasgow RE, Vogt TM, Boles SM. Evaluating the public health impact of health promotion interventions: the RE-AIM framework. Am J Public Health. 1999;89(9):1322-7.

S1 Appendix: Initial Search terms by database

S1 Appendix Initial Search Criteria

Click here to access/download
Supporting Information
S1 Appendix.docx

PRISMA-P Checklist

Click here to access/download
Supporting Information
PRISMA-P-checklist.docx
