# Supplementary material for: Methodologies for studying depression in persons living with tuberculosis: Protocol for a scoping review
Source: PLoS One. 2025 Jul 21;20(7):e0322753. doi: 10.1371/journal.pone.0322753 (PMC12279151; doi:10.1371/journal.pone.0322753)
Supplement: Attachment — Submitted filename: PONE-D-25-16622_R1_Mickael Essouma comments.pdf [file pone.0322753.s005.pdf]

PLOS ONE

Methodologies for studying depression in persons living with tuberculosis: Protocol for a scoping review

--Manuscript Draft--

**Manuscript Number:**	PONE-D-25-16622R1
**Article Type:**	Study Protocol
**Full Title:**	Methodologies for studying depression in persons living with tuberculosis: Protocol for a scoping review
**Short Title:**	Depression and TB: Protocol for Scoping Review
**Corresponding Author:**	Amanda J Gupta, Master of Public Health<br>Yale University<br>New Haven, Connecticut UNITED STATES OF AMERICA
**Keywords:**	tuberculosis; Depression; global health; reviews
**Abstract:**	Tuberculosis (TB) and depression frequently co-occur, yet research has largely focused on prevalence rather than diagnostic or treatment methodologies. Given overlapping symptoms, robust research approaches are critical for improving detection and treatment strategies. This protocol defines a scoping review that aims to map methodologies used to study depression in individuals with TB, identifying gaps in research design, diagnosis, and treatment that may hinder clinical and public health advancements. A search was conducted in MEDLINE, Embase, PsycINFO, Global Health, Cochrane Library, and Africa-Wide Information using controlled vocabulary related to TB and depression. Studies examining the TB-depression relationship will be included. We will use Covidence to facilitate screening, selection, and data extraction. We will extract data on study design, diagnostic tools, treatment interventions, and analytical approaches. Descriptive characteristics of included studies will be presented using figures and tables. Using the RE-AIM framework, we will evaluate reach by assessing the extent to which studies explore both directions of the TB-depression relationship and include diverse, high-risk populations. Effectiveness will be examined by categorizing study designs to assess methodological diversity and evaluating the diagnostic tools and treatment interventions used, along with their reported efficacy. Adoption will be analyzed by identifying where and by whom depression diagnosis and treatment methodologies have been implemented and whether research findings have influenced clinical guidelines or public health policies. Implementation will be assessed by identifying barriers and facilitators reported in qualitative studies or by researchers regarding the integration of depression care into TB treatment settings. Finally, maintenance will be determined by examining whether diagnostic and treatment methodologies were sustained within health systems, including long-term patient outcomes and the persistence of interventions beyond the research phase. By mapping existing methodologies and identifying research gaps, this review will provide valuable insights to guide future research study designs and improve diagnostic and treatment strategies.
**Order of Authors:**	Amanda J Gupta, Master of Public Health
	Patricia Turimumahoro
	Lori Rosman
	Jonathan E Golub
	David W Dowdy
**Opposed Reviewers:**	
**Response to Reviewers:**	Dear Reviewers<br><br>We thank the review team for their careful consideration of our protocol manuscript. We believe the reviewers suggestions will improve both this protocol manuscript as well as our scoping review. Below we have responded to the reviewers suggestions as well as made changes where appropriate to the manuscript itself.

Powered by Editorial Manager® and ProduXion Manager® from Aries Systems Corporation

Best
Amanda J Gupta

Journal Requirements:

1. Please ensure that your manuscript meets PLOS ONE's style requirements, including those for file naming.

Please note, we have updated the formatting to meet PLOS ONE’s style requirements. Thank you for directing us to the appropriate guides for doing so.

2. When completing the data availability statement of the submission form, you indicated that you will make your data available on acceptance. We strongly recommend all authors decide on a data sharing plan before acceptance, as the process can be lengthy and hold up publication timelines. Please note that, though access restrictions are acceptable now, your entire data will need to be made freely accessible if your manuscript is accepted for publication. This policy applies to all data except where public deposition would breach compliance with the protocol approved by your research ethics board. If you are unable to adhere to our open data policy, please kindly revise your statement to explain your reasoning and we will seek the editor's input on an exemption. Please be assured that, once you have provided your new statement, the assessment of your exemption will not hold up the peer review process.

We have updated the data availability statement to indicate that in the present submission, there is no data generated as this is a protocol manuscript.

Additional Editor Comments:

Consider complying with PLOS One guidelines for formatting manuscripts. Accordingly, the authors are urged to not subdivide the abstract into sub-sections. Furthermore, the authors are not expected to present results in any section of the scoping review protocol article given that the study results are not expected at this stage of the research process.

Thank you. We have updated the abstract to reflect this guidance. While this study itself has no results, we still believe there is a benefit in having a results section for the main body of the manuscript as it outlines how we will report the results of our scoping review.

I appreciate the figures provided regarding the epidemiologic and economic burden of TB. However, the authors could strengthen this paragraph by starting the introduction with the definition and classification (by the type of organ involvement: pulmonary versus extrapulmonary [specification of the extrapulmonary body organs that can be afflicted by TB required] TB;by disease activity: active TB versus latent TB; and by drug resistance: MDR-susceptible TB and MDR-resistant TB of tuberculosis) of TB, especially since PLOS One is a generalist journal. This would set the stage for the authors to make comments about TB throughout the manuscript (not just in the introduction) based on its subtypes, because there are indeed differences between pulmonary TB and extrapulmonary TB in the one hand, and between MDR-susceptible TB and MDR-resistant TB on the other hand. As a result, there may also bedifferences in the burden of depression across TB subtypes

Thank you for making this point. To ensure all your readers, including those not as well versed in TB, understand the burden that TB poses as well as the disease itself, we have updated the intro paragraph (lines 65-82) to the following to highlight the different types of TB along with their burden:

Tuberculosis (TB), a communicable disease caused by Mycobacterium tuberculosis, is one of the leading infectious killers worldwide. While it primarily affects the lungs (pulmonary TB), it can be found in other places throughout the body (extrapulmonary TB) such as in the lymph nodes, pleura, bone and joints, urogenital tract, and meninges to name a few. The COVID-19 pandemic accelerated deaths due to TB as

Powered by Editorial Manager® and ProduXion Manager® from Aries Systems Corporation

less attention, and subsequently funding, has been paid to TB (1, 2). This resulted in an estimated 10.8 million people developing new cases of active TB in 2023 with an estimated 1.25 million deaths due to TB disease (2). Active TB refers to disease in which TB bacteria are replicating and causing clinical symptoms. Pulmonary forms of active TB are typically symptomatic and infectious while extrapulmonary TB is usually symptomatic but not transmissible. This contrasts with latent TB which is a state in which an individual is infected with TB but is generally asymptomatic and non-infectious. It is estimated that billions of individuals worldwide have latent TB. Furthermore, TB can be classified by drug resistance with ~400,000 PWTB having multidrug-resistant TB (resistance to at least isoniazid and rifampin) in 2023 (2). Despite this large burden, TB is vastly underfunded leading to under-supported research endeavors and national TB programs left without appropriate funding to successfully combat the large TB burdens that they may face (1-5). This results in gaps in TB diagnosis, treatment, and care in high TB burden settings (6-8).

In addition, we added in the following sentence (line 111-126) to reflect the fact that there may exist differences in depression burden by TB subtype:

Additionally, the burden of depression may also vary by TB subtype —individuals with extrapulmonary TB may face diagnostic delays and uncertainty that heighten psychological distress (26,27), while those with multidrug-resistant TB (MDR-TB) often endure prolonged treatment, more severe side effects, and greater social isolation, all of which may increase the risk or severity of depression (28,29).

Consider clarifying this statement. The revised statement could read: "While the diagnosis and treatment protocols of infectious TB comorbidities such as Human Immunodeficiency Virus (HIV) infection have been extensively researched, the diagnosis and treatment of comorbid non-communicable diseases (NCDs) such as mental health disorders have received little attention, even as the identification and treatment of comorbid NCDs has been shown to positively impact the TB outcome in affected patients."

We have updated the sentence to incorporate your suggested wording (see lines 85-92).

This sentence needs to be supported by at least one reference. Furthermore, it would be great to provide a multiperspective definition of depression after this sentence.

Suggested references:
1. PMID: 38357431 PMCID: PMC10863678 DOI: 10.32872/cpe.11699
2. https://doi.org/10.1016/j.socscimed.2016.12.030
3. PMID: 38856993 DOI: 10.1001/jama.2024.5756

Thank you for not only the suggestion but providing wonderful citations to go along with it! We have added in two TB specific citations for the original sentence in question (line 95) and have now added the following sentences (lines 95-100):

Depression is a common mental health condition characterized by persistent sadness, loss of interest or pleasure, and functional impairment which can affect the overall quality of life of individuals suffering from it if not properly managed (18). While clinically defined in various tools, such as the DSM-5, its expression and recognition vary across cultural and social contexts increasing the diagnostic and treatment complexity of the condition (19).

Consider revising this paragraph to address the mechanisms (including the high frequency of HIV infection in the population with TB) underpinning the TB-depression syndemic, challenges in and facilitators for diagnosing and treating depression across TB subtypes, and the public health impact of diagnostic and therapeutic interventions targeting depression in individuals with TB.
In addition to the references provided by reviewer 1, these references could be helpful for this purpose:
1. PMID: 34140898 PMCID: PMC8203803 DOI: 10.3389/fpsyt.2021.617751
2. Wang J, Wu X,

Powered by Editorial Manager® and ProduXion Manager® from Aries Systems Corporation

	Lai W, et al. Prevalence of<br>depression and depressive<br>symptoms among outpatients:<br>a systematic review and<br>meta-analysis. BMJ Open<br>2017;7:e017173. doi:10.1136/<br>bmjopen-2017-017173 for depression diagnosis in TN patients<br>3. https://doi.org/10.1016/j.genhosppsych.2021.01.006<br>4. https://doi.org/10.1038/s41392-024-01738-y<br><br>We have re-worked the introduction extensively (Lines 104-126) in an attempt to better capture the complexity of the underlying mechanisms of the TB-depression syndemic, and have now included the example of co-infection with HIV as an example of a potential underlying mechanism. We greatly appreciate your suggestion of references as well as those of Reviewer #1 and hope that our re-working of the introduction has now addressed your concerns.<br><br>I am unable to grasp the message conveyed by this statement.<br><br>We apologize for any lack of clarity. We have re-worked the sentence in question to the following (lines 130-131):<br><br>From a public health perspective, untreated depression contributes to delays in TB diagnosis and treatment initiation (26, 28), non-adherence to TB medications (29, 30), and ultimately increases negative TB outcomes such as loss to follow up and death (31, 32).<br><br>This last part of the introduction should be revised to clearly state the rationale for conducting a scoping review on this topic and the aim of this protocol article. Based on the cover letter and the fact that the authors will use the RE-AIM framework for data synthesis, the rationale for this scoping review seems to be the need to guide future studies by providing a roadmap for integrating appropriate (from a public health perspective) diagnostic and therapeutic interventions for depression into routine care of different forms of TB: see Munn et al. BMC Medical Research Methodology (2018) 18:143<br>https://doi.org/10.1186/s12874-018-0611-x.<br>Alonmg with my comment about the rationale for the scoping review, a simpler delineation of the aim of this scoping review protocol article could be: to evaluate the public health impact of diagnostic and therapeutic interventions for depression in individuals with different forms of TB: see PMID: 10474547 PMCID: PMC1508772 DOI: 10.2105/ajph.89.9.1322.<br><br>Thank you for this suggestion. We have updated the final paragraph of the introduction as follows (lines 165-175):<br><br>Given the substantial burden of depression among PWTB and the large focus on prevalence estimates within the literature with limited exploration of the methodological diversity and public health relevance of diagnostic and therapeutic approaches, a scoping review is particularly needed to broadly map the existing evidence, identify the most pressing knowledge gaps, and clarify how research has been conducted when studying these comorbid conditions. A scoping review would also help guide future studies by offering a roadmap for integrating effective and contextually appropriate mental health interventions into TB care. Therefore, we aim to conduct a scoping review to examine the methodologies used to study depression, including diagnostic and treatment methodologies, in PWTB, identifying research gaps and highlighting opportunities for improved methodological approaches in future public health research.<br><br>Because this is the protocol (not the scoping review) article, I suggest revising this statement in these likes:<br><br>"This section is presented in accordance with the scoping review framework by Arksey and O'Malley [26] and refined by Levac et al [27] which will guide the methodology of our scoping review."

	Did you also register the protocol in OSF for example? Pieper and Rombey Systematic Reviews (2022) 11:8<br>https://doi.org/10.1186/s13643-021-01877-1<br><br>Thank you. We have revised the sentence per your suggestion. We have not registered the protocol in OSF yet but as is now mentioned in manuscript (lines 326-329) we will be making all relevant data from the review available in OSF upon completion of the review and will have the protocol registered prior to data extraction<br><br>Along with the potential rationale and aim of the scoping review mentioned above, is this question not more exact:<br><br>"What methods have been used so far to diagnose and treat depression across different forms of TB and did those methods have sizeable public health effects?<br>We thank you for your consideration of our research question. We believe that our current research question captures the aim of our review as we will not be able to answer the second part of your proposed question (“did those methods have sizeable public health effects”) since we will not be conducting a meta-analysis of any diagnostic tools or treatment interventions. We are seeking to have a broad inclusion to see how researchers have tried to define the relationship between TB and depression. Studies may also be characterizing the depression risk profile of those with TB which would also be of interest beyond just treatment and diagnosis. That being said, depending on the results of the present scoping review, conducting a follow-up systematic review and meta-analysis looking at the public health impact of treatment interventions may be an appropriate next step.<br><br>Consider deleting this statement to leave only the research question in this sub-section.<br><br>We have deleted the sentence “We will screen studies that have sought to examine the relationship between TB and depression”<br><br>Can you reframe this statement to first enumerate the searched electronic databases and then state the reasons why you are planning to search those databases?<br><br>We have replaced the noted sentence with the following (lines 194-202):<br><br>We will search the following electronic databases: MEDLINE, Embase, Global Health, the Cochrane Library, the WHO Regional Libraries, Africa-Wide Information, and PsycINFO. These databases were selected for their relevance to the scope of this review: MEDLINE, Embase, Global Health, and the Cochrane Library are widely used for biomedical and public health research; the WHO Regional Libraries and Africa-Wide Information are included to ensure geographic representation, particularly from high TB burden regions such as sub-Saharan Africa; and PsycINFO is included for its comprehensive coverage of psychological and mental health research.<br>Could you revise this statement to clearly specify that the electronic search will be supplemented by manual searches consisting of the scrutinization of reference lists of studies retrieved from electronic search? Will you also conduc grey literature searches? Notably, I anticipate that there are non-peer reviewed/non-published materials addressing this topic in a subset of African TB/mental health settings.<br>We have updated the referred to sentence to the following (lines 219-222):<br>Finally, the electronic database search will be supplemented by manual searches, including the screening of reference lists from all included studies to identify any additional relevant articles that may have been missed.<br>We appreciate the suggestion to include grey literature and strongly considered it prior to the initial submission as well as again now. However, given the methodological focus of this scoping review and the resource constraints of our research team, we have chosen to limit our search to peer-reviewed literature to ensure consistency in reporting standards and methodological detail, which are often less clearly documented in grey literature sources, as we need studies fully detailing their methods in order to answer our research question.<br>Consider adding details about participants' age, sex and socioeconomic status (JBI Evid Synth. 2020 Oct;18(10):2119-2126. doi: 10.11124/JBIES-20-00167.)<br>Consider also mentioning a statement about comparators of participants with the TB-

	depression syndemic given that you will include interventional studies.<br>Within Table 1, we have added in wording to explicitly state that participants of any age, sex, or SES will be included.<br>I suggest reframing this statement like this:<br>"human participantss with only a clinical diagnosis of tuberculosis, those with a mycobaterial infection not due to M. tuberculosis (e.g., leprosy), and animal participants"<br>The fact that the authors also include participants with latent tuberculosis mean that they cannot exclude individuals considered to be affected by tuberculosis based solely on the presence of M. tuberculosis in biological samples.<br>We have updated Table 1 to make it more legible based on the recommendation of Reviewer 1. We have also integrated the language you mention here. To clarify the point on latent TB, we will include studies involving individuals with either active or latent tuberculosis, as long as there is confirmation of TB infection—regardless of the method used to establish that diagnosis (e.g., microbiological, radiological, or clinical assessment). Our aim is not to restrict inclusion based on a specific TB diagnostic modality but to ensure that participants included in studies have been clearly identified as having TB infection in accordance with the criteria used by the study authors.<br>It seems to me that the concept of the scoping review is "public health impact of diagnostic and therapeutic interventions for depression in all forms of TB". Accordingly, the authors would also like to specify here that they are planning to include any study design that could help assess the public health impact of diagnostic and therapeutic interventions for depression in TB patients. Along with a comment of Reviewer 2 and scoping review guidelines, this scoping review should include all available evidence, regardless of of the publication language, especially since TB is highly prevalent in many countries where English is not among the official languages.<br>Thank you for this insightful comment. We would like to clarify that we will not be able to determine the public health impact of diagnostic and therapeutic interventions for depression in PWTB as we are not proposing to conduct a meta-analysis. Rather, we are focused on establishing the current state of the literature and highlight methodological gaps that exist in the study of these two co-morbid conditions. Regarding language restrictions, while we recognize the importance of including non-English language publications—particularly given the global burden of TB—we must balance this with the capacity of our research team. Therefore, we regrettably must restrict the search to English language studies as we lack funding for translations and do not have at least 2 team members who speak additional languages beyond English. We will document and transparently report any exclusions based on language to acknowledge this as a potential limitation. This approach aims to maximize the inclusiveness of the review while remaining feasible within available resources.<br>I suggest splitting this arrow into two arrows: one on study setting and another one on the individuals who assessed the public health impact of diagnostic and therapeutic interventions for depression in TB patients. In the study setting arrow, the authors should report on i) the continental coverage of studies to be included, ii) countries where studies to be included should have been conducted, iii) study localities, i.e., urban, semi-urban, and rural; and housing platforms where included studies were conducted: community centres vs hospital centres, unspecialized versus specialized (TB centres versus mental health/depression centres versus TB-mental health centres [e.g., Jamot Hospital in Yaounde-Cameroon]) centres, and public health centres versus centres for purposes other than public health promotion. The second arrow would report on whether you will include studies regardless of credentials of individuals who evaluated the public health impact of diagnostic and therapeutic interventions for depression in individuals with TB: public health practioners versus others.<br>Thank you for this suggestion. We have re-worked Table 1 based on Reviewer #1's suggestions and believe that legibility and clarity has been improved. Many of the suggestions you make here are in our data extraction template for study details and we will be sure to report on them for our findings. Here we are wanting to make sure that the reader understands that the context is largely not determining the inclusion in this scoping review as long as the study has taken place within humans (i.e. not veterinary settings and not laboratory only settings)<br>This statement should come just after the statement on the searched databases in lines 109 to 113.<br><br>Could you try adding the forms of TB in preliminary search strategies and see whether this optimizes the sensitivity and specificity of search strategies?

	PMID: 30271302 PMCID: PMC6148622 DOI: 10.5195/jmla.2018.283<br><br>McGowan et al. Journal of Clinical Epidemiology 75 (2016) 40e46<br><br>Duko et al. Ann Gen Psychiatry (2020) 19:30<br>https://doi.org/10.1186/s12991-020-00281-8<br><br>Consider mentioning the search strategies in tables in the main manuscript. The search strategy is a core part of a scoping review protocol article.<br>Thank you for suggesting this. Per your suggestion, we have now moved the referred to sentence to right after our discussion of databases (Lines 202-216).<br>Since we are using MESH terms and terminology suggested by the respective databases, we have captured all various forms of tuberculosis in the current search strategy for each database. Our team librarian (LR) ran many test searches prior to arriving on this particular strategy to ensure all types of TB would be included and all possible articles discussing depression and TB co-occurrence would be included as well.<br>While we agree that the search strategies are a core part of the scoping review protocol, we also recognize that they do not translate well to tables/figures. We would prefer to keep it as a supplemental document to ensure the appropriate formatting for each individual database is maintained for those readers who are interested in replicating or updating our results.<br>Would you mind replacing this phrase with "immunosuppressive status" to also consider other causes of immunosuppression?<br>Thank you for this thoughtful suggestion. While we understand the rationale for broadening the HIV phrasing to "immunosuppressive status," we have chosen to retain the specific reference to HIV in this context. Given the well-documented and disproportionately high prevalence of HIV co-infection among people with tuberculosis, as well as its significant implications for both TB progression and mental health outcomes, HIV remains the most relevant and commonly reported immunosuppressive condition in TB research. As such, the research team believes that specifying HIV is both appropriate and necessary to accurately reflect the epidemiological and clinical realities in this population. We will not however limit our data extraction to only HIV related immunosuppressive states if a study does report on it. We just believe this is the most relevant to provide as an example.<br>Consider adding socioeconomic and nutritional status which are also important social determinants of TB.<br>Thank you. We have now added in.<br>Can you specify the theme analysis frameworks used in qualitative studies in brackets?<br><br>Medwave. 2024 Nov 22;24(10):e2972. doi: 10.5867/medwave.2024.10.2972.<br><br>We have now added in “(i.e. thematic analysis, grounded theory, case study approach, etc.)” (lins 268-269).<br><br>I see "narrative synthesis" nowhere in this paragraph despite this form of data synthesis being the main one used in the scoping review design.<br><br>Thank you for this comment. We recognize that narrative synthesis is commonly used in scoping reviews; however, for this study, we have intentionally chosen not to adopt a narrative synthesis approach. Instead, we will apply a structured framework-based synthesis using the RE-AIM framework to enhance methodological transparency and rigor. This approach allows us to systematically categorize and analyze the methodological characteristics of included studies in relation to public health impact, rather than relying on a primarily descriptive or thematic narrative. We believe this framework-guided strategy will provide greater clarity and utility for guiding future research and informing intervention development.<br><br>Can you specify the risk of bias assessment tools you plan to use?<br><br>Certainly! We have now added in the following sentence (Line 281-284):

	As there will be many different study designs, to assess quality, we will use the Jadad scale for randomized controlled trials, the Newcastle-Ottawa scale for observational studies, and the Joanna Briggs Institute Critical Appraisal Tool for Qualitative Research for qualitative studies<br><br>Will it not be more precise to say that you will assess the public health impact of diagnostic and therapeutic interventions for depression using the RE-AIM framework?<br><br>We will not be assessing the public health impact in this scoping review. We will be characterizing the state of existing literature. Assessing the public health impact could be done in a follow-up systematic review and meta-analysis depending on the findings of the current scoping review.<br><br>I suggest revising the text in lines 166-192 to clearly specify how the public health impact of depression diagnostic and therapeutic interventions will be assessed through the RE-AIM model ((Am J Public Health<br>. 1999 Sep;89(9):1322-7. doi: 10.2105/ajph.89.9.1322.). The revised text could read: "R for Reach, refers to the evaluation of how well depression diagnostic and therapeutic interventions were delivered to the target populations/high-risk TB groups. E for efficacy, refers to the assessment of the accuracy (sensitivity, specificity, AUC , etc) of depression diagnostic methods, efficacy of depression treatment methods, as well as cost-benefit, cost-utility and cost-effectiveness (Iran J Public Health<br>. 2022 Oct;51(10):2159-2170. doi: 10.18502/ijph.v51i10.10975.) of depression diagnostic and treatment methods in individuals with different forms of tuberculosis. A for adoption, refers to proportions of the different individuals or legal/regulatory bodies that adopted depression diagnostic and treatment methods as well as barriers and facilitators to the adoption of depression diagnostic and intervention methods in TB patients. I for implementation, refers to... M for mainetnance, refers to...<br><br>Thank you for your thoughtful suggestion. We appreciate the recommendation to align our use of the RE-AIM framework with more traditional applications that focus on evaluating the public health impact of interventions. However, we would like to clarify that this scoping review is not designed to evaluate the effectiveness or real-world impact of depression diagnostic or therapeutic interventions among individuals with tuberculosis. Rather, the purpose of this review is to map and characterize the methodologies used in the existing literature—including how depression is studied, diagnosed, and treated in TB populations—and to identify knowledge gaps that may inform future research and implementation efforts.<br>As such, we are applying the RE-AIM framework not to assess intervention outcomes per se, but to organize and interpret how existing studies reflect various dimensions relevant to public health research, such as who is being studied (Reach), what methodological designs are being used (Effectiveness, as a proxy), where and by whom depression-related methods are applied (Adoption), what contextual barriers and facilitators are reported (Implementation), and whether there is any evidence of longer-term integration (Maintenance). This use of RE-AIM aligns with its broader utility as a planning and evaluative framework that can guide the interpretation of complex evidence across multiple settings, even in the absence of formal impact evaluation (Glasgow et al., 1999).<br>Consider addressing the "Cosnultation with stakeholders" sub-section of the O'Malley and Arksey framework just before this sub-section.<br><br>This sub-title should be in bold because it points to a sub-section.<br><br>Consider adding four sub-sections after this one and before the discussion section:<br><br>-Patient and public involvement (if you include patients/patient partners, how will you select them?)<br>-Dissemination and ethics<br>-Data sharing (in line with Reviewer 1's comment)<br>-Protocol deviation (is it possible that the protocol be modified even after the publication of this article? how will you deal with that issue in case it happens?)?<br><br>Rather than include both a section on patient/public involvement and a section on consultation with stakeholders, we have added in a section on Consultation with

	Stakeholders that explicitly states that we will draw on a diverse group of stakeholders, including PWTB currently participating in an ongoing and unrelated cohort study in Uganda to ensure that all conclusions and interpretations that are made as part of the scoping review are in line with lived experiences of relevant stakeholders. Given our teams extensive network of TB professionals across many continents, we believe we will be able to make meaningful conclusions about our work by drawing on the various experts within these networks. Please see the newly created section in Lines 316-326.<br><br>As mentioned below in our response to reviewer 1, we have added in Data Sharing (lines 327-330). We believe this covers the dissemination portion you mention above. We do not anticipate any protocol deviations as the protocol is considered "locked" and all team members have been involved in the process of creating this protocol.<br><br>Can you revise this paragraph specifying the strengths of the methods planned to be used in the future scoping review?<br><br>Certainly. While we believe the paragraph in question highlights many of the strengths for our review's aims, we have now added in some additional strengths on the methodological rigor we plan to employ (lines 353-361):<br><br>Additionally, our review will be methodologically rigorous by using the Levac et al framework to ensure a systematic and transparent approach to study identification and selection. This will be enhanced through the use of RE-AIM to synthesize results, providing a structured lens to assess how methodological approaches to researching TB and depression have created knowledge gaps in studying the diseases concurrently. Finally, the planned stakeholder consultations will add depth and practical relevance to the final interpretation of the findings ensuring that the review will be both methodologically rigorous and highly relevant to global TB and mental health research practices.<br><br>The review has not yet been conducted. Would you mind revising this statement delineating the anticipated limitations of the methods planned to be used in the future scoping review? Focus should be made mainly on depression case-identification methods, limitations of the methods planned to be used for study identification, and anticipated limitations of RE-AIM assessment of the public health impact of depression diagnostic and therapeutic methods in individuals with different forms of TB.<br><br>As noted in the timeline section, we have conducted the initial search (on March 31, 2025). We are currently screening titles and abstracts now. Based on your comments and those of Reviewer 1, we have updated the limitations (lines 372-383). As noted above, determining the public health impact is beyond the scope of this review and is a question better suited for a subsequent systematic review and meta-analysis. Please see lines 372-3 for the newly added limitations about scoping review methodologies.<br><br>Third, since the primary aim is to describe and categorize existing research rather than synthesize findings or estimate effect sizes, scoping reviews cannot make definitive conclusions about intervention effectiveness or causality. However, depending on the results of the current review, it may be appropriate to conduct a subsequent systematic review and meta-analysis to assess the public health impact of depression-related interventions amongst PWTB. Finally, as with all scoping reviews, the breadth of study types and topics can make data synthesis more complex than in a systematic review. Given our aim of establishing methodologies used in the field, a broad inclusion is necessary but could provide more narrative results. We believe our use of RE-AIM to structure the results, regardless of study type, will aid in creating meaningful outputs.<br><br>This should also be revised to strengthen it consistently with the aim of the planned scoping review.<br><br>We have re-worded the sentence in question as follows (lines 386-388):<br><br>This scoping review will advance understanding of how depression has been studied in the context of tuberculosis by systematically mapping existing methodologies and identifying critical research gaps to guide future public health research and intervention development.

Powered by Editorial Manager® and ProduXion Manager® from Aries Systems Corporation

Reviewer #1: Review for: “Methodologies for Studying Depression in Persons living with Tuberculosis: Protocol
for a Scoping Review“

The paper at hand is the study protocol for a scoping review about methodologies for studying depression in persons suffering from tuberculosis. The paper is generally well written. The authors refer to established guidelines of conducting scoping reviews. The inclusion of supporting information like the PRISMA checklist or the search terms is laudable. The high prevalence of depression in tuberculosis and the detrimental consequences of both conditions makes this a very important scientific endeavor. In my view this work shows great promise and I applaud the authors for their work. Nevertheless, I have some concerns and comments that I feel need to be addressed. Please find my detailed review below.

My main point concerns the description of the aim of the planned scoping review. The methods section mostly seem to indicate that the aim is to find treatment trials, i.e., trials in which either depression and/or tuberculosis are treated. In the results section you convey that you will consider diverse study types. This seems good because treatment studies are not the only way to find out more about any associations between the two diseases. In any case, after the revision, the paper should give some examples for typical types of studies that you aim to include. I get that you cannot anticipate every type of study that explores the interrelationships between tuberculosis and depression but at the moment it is hard to grasp how do you aim to get relevant information on comorbidity and treatment options and whether interventional studies are the backbone of your review or one source of many. I don't think there is necessarily a problem with the content here, but rather difficulties arise due to the manner in which the research plan and the narrative flow of the text are presented.
Thank you for your thoughtful review and comments. We believe that we have updated the methods section to better reflect the fact that we will be going far beyond treatment studies for the present review but rather capturing any study that is aiming to characterize the relationship between TB and depression. We believe that our re-design of Table 1 (based on one of your later comments) in particular now makes it quite clear that we are aiming for a broad range of studies in order to characterize methodological gaps that exist in the study of comorbid depression and TB. We hope that our edits have made the narrative flow more clear to ensure readers understand the aim of our review.

The second major point that strikes me is the theoretical embedding of the connections between tuberculosis and depression. It is quite well deduced that it exists and that it should be investigated further. However, it does not go into what previous findings have shown as to why this connection exists. I am not an expert in this field. However, I wonder what psychological and/or biological factors could be interacting here? Is there relevant biopsychological literature regarding the interrelationship? What about the neuroscience of tuberculosis?

I also wonder whether meta-theories such as the “complex systems” perspective (which I am more familiar with) could be utilized to explain the relationships?
Fried, E. I., & Robinaugh, D. J. (2020). Systems all the way down: embracing complexity in mental health research. BMC medicine, 18, 1-4.

Scheffer, M., Bockting, C. L., Borsboom, D., Cools, R., Delecroix, C., Hartmann, J. A., ... & Nelson, B. (2024). A dynamical systems view of psychiatric disorders—theory: a review. JAMA psychiatry, 81(6), 618-623.

Westhoff, M., Berg, M., Reif, A., Rief, W., & Hofmann, S. G. (2024). Major problems in clinical psychological science and how to address them. Introducing a multimodal dynamical network approach. Cognitive Therapy and Research, 48(5), 791-807.
Thank you for highlighting the complexity of TB and depression co-occurrence. It is clear that you carefully reviewed our manuscript with the intention of improving our thinking and approach and we are deeply appreciative of your careful consideration and advice.
We have updated our introduction extensively and have included examples of the biological and psychological interactions (detailed more below). We have also taken

	your suggestion about highlighting complex systems thinking and have used many of the references you suggested to highlight the utility in using systems thinking as an approach to TB and depression. Please see the newly added sentences about systems thinking (lines 142-163):<br>Given this, a complex systems thinking approach (37-39) may be of use to researchers as it highlights the importance of moving beyond isolated risk factors to better consider the dynamic, bidirectional, and context-dependent interactions between TB and depression. This perspective also underscores the need for diverse and multi-level methodological approaches that can better capture feedback loops (e.g. how depression worsens TB outcomes, which may in turn exacerbate depressive symptoms), variability across TB subtypes, and health system contexts. Furthermore, applying such a lens can help identify critical gaps in the current research landscape and more effectively guide future investigative efforts.<br><br>More importantly, there seem to be reviews that discuss possible reasons for mental health problems in tuberculosis patients that you could cite (or discuss in more depth).<br>Cite:<br><br>Alene, K. A., Wangdi, K., Colquhoun, S., Chani, K., Islam, T., Rahevar, K., ... & Viney, K. (2021). Tuberculosis related disability: a systematic review and meta-analysis. BMC medicine, 19, 1-19.<br>Discuss in more depth:<br><br>Hayward, S. E., Deal, A., Rustage, K., Nellums, L. B., Sweetland, A. C., Boccia, D., ... & Friedland, J. S. (2022). The relationship between mental health and risk of active tuberculosis: a systematic review. BMJ open, 12(1), e048945.<br><br>Cáceres, G., Calderon, R., & Ugarte-Gil, C. (2022). Tuberculosis and comorbidities: treatment challenges in patients with comorbid diabetes mellitus and depression. Therapeutic Advances in Infectious Disease, 9, 20499361221095831.<br><br>I get that an extensive discussion might be to much for a protocol paper but some hints into the possible nature of the relationships should be given and be it on a meta (e.g., systems) level. For a protocol paper, I think that one to two paragraphs are enough but the reader will need some context information here.<br>We have now added additional details and examples into the introduction about the intersecting biological, social and structural factors that can increase the risk or severity of depression for those living with TB. We have also added in numerous references, including many that you suggest here. Please see the entire introduction which has been extensively re-worked or this excerpt here which focuses on the mechanisms underlying depression and tuberculosis (lines 104-126):<br>Depression may not only be a consequence of TB, but also a potential risk factor due to its effects on immune function, health-seeking behavior, and treatment engagement (21, 22). Furthermore, TB-depression co-occurrence can be thought of as a syndemic, or an interacting set of conditions that cluster within social disadvantage, including intersecting biological, social, and structural factors, that results in an increased burden of disease (23-25). For example, HIV infection, a highly prevalent co-morbidity in PWTB, contributes to immunosuppression, social stigma, and economic vulnerability, all of which heighten susceptibility to mental health disorders (20). Additionally, the burden of depression may also vary by TB subtype —individuals with extrapulmonary TB may face diagnostic delays and uncertainty that heighten psychological distress (26, 27), while those with multidrug-resistant TB (MDR-TB) often endure prolonged treatment, more severe side effects, greater economic uncertainty, and greater social isolation, all of which may increase the risk or severity of depression (28, 29)<br><br>Finally, the protocol should have a statement about open science practices. Will the extraction sheet be made openly available? How do you plan to share important study metadata? If you aim to share your data, at which repository do you want to share it? If you do not aim to share your data, this deserves justification in my view.<br>We have now added the following sentence (lines 327-330):<br>In line with open science principles, we will make the final data extraction sheet and study metadata openly available through a public repository such as the Open Science Framework upon completion of the review.

Powered by Editorial Manager® and ProduXion Manager® from Aries Systems Corporation

	Minor<br><br>Sometimes you seem to use [] for indicating your sources and sometimes you use ().<br>Please stick to a standardized style of reference indicators.<br>Thank you. We have now ensured that our citations fully comply with PLOS ONE guidelines.<br><br>The list of references is quite short at the moment. I am aware that this is a protocol paper but I would think that the paper would benefit from additional sources.<br>We have now greatly added to the overall number of references based on your suggestions as well as those of the other reviewers. Thank you for this suggestion as well as specific suggestions of papers to cite which we believe have greatly enhanced the rigor of our manuscript.<br><br>Line 55: "is one of the leading infectious killers worldwide": I would recommend against using "killer" and instead use words like lethality / mortality here. Furthermore, I would recommend you cite relevant literature to support this (true) claim.<br>We have altered the phrasing to now say "leading infectious causes of mortality" and cite the Global Tuberculosis Report 2024.<br><br>Line 74: Please remove the "staggering".<br>We have removed "staggering" as you suggest.<br><br>Table 1 contains long sentences and is really difficult to read. I think it needs substantial rework. I would recommend that you shorten the text, consider using bullet points instead of sentences and consider using spacing options to make your points clearer.<br>We have reformatted Table 1 with bullet points as you suggest as well as removed some wording. We believe this has improved the legibility of the table.<br><br>Line 115: Good that you screen the references of the included papers. Consider including relevant literature for the benefits<br>of footnote chasing.<br>Thank you for this suggestion. We agree that citation chasing can be a valuable method for identifying additional relevant studies. However, given the scope of our initial database search, which returned over 15,000 citations, and the resource constraints of our research team, we have opted to limit citation chasing to the references of included studies only, as originally planned. We believe this approach balances comprehensiveness with feasibility and aligns with the methodological standards for scoping reviews.<br><br>Limitations section: I would recommend that you critically discuss the strength and shortcomings of scoping reviews compared to traditional systematic reviews and meta-analyses and justify your choice for the scoping review. What I like about the limitations section is the critical discussion of cultural aspects.<br>We have added in additional limitations based on the general study design as follows (lines 372-383):<br>Third, since the primary aim is to describe and categorize existing research rather than synthesize findings or estimate effect sizes, scoping reviews cannot make definitive conclusions about intervention effectiveness or causality. However, depending on the results of the current review, it may be appropriate to conduct a subsequent systematic review and meta-analysis to assess the public health impact of depression-related interventions amongst PWTB. Finally, as with all scoping reviews, the breadth of study types and topics can make data synthesis more complex than in a systematic review. Given our aim of establishing methodologies used in the field, a broad inclusion is necessary but could provide more narrative results. We believe our use of RE-AIM to structure the results, regardless of study type, will aid in creating meaningful outputs.<br><br>Line 219: "Our review does have": Maybe write: "Our review will have some limitations that are worth pointing out a priori"... Right now it sounds as if the review was already conducted. Down below in the conclusions section you use "will" and I would recommend also using it here.<br>We have adopted the language you propose here (Line 363).

Powered by Editorial Manager® and ProduXion Manager® from Aries Systems Corporation

I support an open and transparent review process. Therefore, I do not wish to stay anonymous.
Thanks to the authors and to the journal editors for giving me the opportunity to review this paper.

Max Berg,

University of Marburg
Clinical Psychology Group

Again, we would like to thank you for your thoughtful and constructive review. We believe it has enhanced our protocol manuscript and we are grateful that you participated in this peer review process.

Reviewer #2: The article is a study protocol for a scoping review aimed at mapping methodologies used to study depression in individuals with tuberculosis (TB). The primary objective is to identify gaps in research design, diagnostic tools, and treatment strategies for depression in TB patients. The authors highlight the co-occurrence of TB and depression and the need for robust research approaches to improve integrated care. The protocol outlines a systematic approach to reviewing existing literature, with the goal of guiding future research and public health interventions.

Methodology Evaluation:
The study employs the Arksey and O'Malley framework, updated by Levac et al., for conducting scoping reviews, which is appropriate for mapping broad research areas. The protocol includes six steps: identifying the research question, selecting relevant studies, charting data, collating results, and consulting stakeholders. The search strategy is comprehensive, covering databases like MEDLINE, Embase, PsycINFO, and Africa-Wide Information, ensuring a global perspective. Inclusion criteria are well-defined, focusing on studies that examine the TB-depression relationship, while exclusion criteria eliminate irrelevant or low-quality studies.
Thank you for this summary and highlighting some of the strengths of our approach!

Data extraction will be performed using Covidence, with two reviewers screening abstracts and full texts to minimize bias. The RE-AIM framework (Reach, Effectiveness, Adoption, Implementation, Maintenance) will structure the analysis, which is a strength for evaluating the impact of research methodologies. However, the protocol limits the search to English-language studies, potentially excluding relevant research from high-TB-burden, non-English-speaking countries. Additionally, the reliance on Western terms like "depression" may overlook culturally specific expressions of mental health conditions.
As mentioned above, regarding language restrictions, while we recognize the importance of including non-English language publications—particularly given the global burden of TB—we must balance this with the capacity of our research team. Therefore, we regrettably must restrict the search to English language studies as we lack funding for translations and do not have at least 2 team members who speak additional languages beyond English. We will document and transparently report any exclusions based on language to acknowledge this as a potential limitation. This approach aims to maximize the inclusiveness of the review while remaining feasible within available resources.
You are right to highlight the reliance on Western terms and we were sure to specify it as a key limitation in our discussion section. After running test searches, we expanded our criteria to include broad keywords such as "Mental Health", "Mood Disorder", "Mental Disorder" and other similar words (please see S1 Appendix). We determined that these types of keywords resulted in studies from all continents and not just Western countries. We do however know that it is a limitation of our approach and why we have reported it here (lines 364-369), and will continue to report it in our subsequent findings.

Strengths and Weaknesses:
Strengths:

Comprehensive search strategy across multiple databases.

Clear inclusion/exclusion criteria to ensure relevance.

	Use of the RE-AIM framework to evaluate methodological impact.<br><br>Dual-reviewer process to reduce bias in study selection.<br><br>Weaknesses:<br><br>Language restriction (English-only) may exclude significant studies.<br><br>Potential cultural bias in defining depression, missing non-Western per...
**Additional Information:**	
**Question**	**Response**
**Financial Disclosure**<br><br>Enter a financial disclosure statement that describes the sources of funding for the work included in this submission. Review the submission guidelines for detailed requirements. View published research articles from *PLOS ONE* for specific examples.<br><br>This statement is required for submission and **will appear in the published article** if the submission is accepted. Please make sure it is accurate.<br><br>**Funded studies**<br>Enter a statement with the following details:<br>• Initials of the authors who received each award<br>• Grant numbers awarded to each author<br>• The full name of each funder<br>• URL of each funder website<br>• Did the sponsors or funders play any role in the study design, data collection and analysis, decision to publish, or preparation of the manuscript?<br><br>Did you receive funding for this work?	The author(s) received no specific funding for this work.
**Competing Interests**<br><br>Use the instructions below to enter a competing interest statement for this submission. On behalf of all authors, disclose any competing interests that could be perceived to bias this work—acknowledging all financial support and any other relevant financial or non-financial competing interests.	The authors have declared that no competing interests exist

This statement is required for submission and **will appear in the published article** if the submission is accepted. Please make sure it is accurate and that any funding sources listed in your Funding Information later in the submission form are also declared in your Financial Disclosure statement.<br><br>View published research articles from *PLOS ONE* for specific examples.<br><br>**NO authors have competing interests**<br><br>Enter: *The authors have declared that no competing interests exist.*<br><br>**Authors with competing interests**<br><br>Enter competing interest details beginning with this statement:<br><br>*I have read the journal's policy and the authors of this manuscript have the following competing interests: [insert competing interests here]*<br><br>* typeset	
**Ethics Statement**<br><br>Enter an ethics statement for this submission. This statement is required if the study involved:<br><br>• Human participants<br>• Human specimens or tissue<br>• Vertebrate animals or cephalopods<br>• Vertebrate embryos or tissues<br>• Field research<br><br>Write "N/A" if the submission does not require an ethics statement.<br><br>General guidance is provided below. Consult the submission guidelines for detailed instructions. **Make sure that all**	N/A

information entered here is included in the Methods section of the manuscript.<br><br>**Format for specific study types**<br><br>**Human Subject Research (involving human participants and/or tissue)**<br>• Give the name of the institutional review board or ethics committee that approved the study<br>• Include the approval number and/or a statement indicating approval of this research<br>• Indicate the form of consent obtained (written/oral) or the reason that consent was not obtained (e.g. the data were analyzed anonymously)<br><br>**Animal Research (involving vertebrate animals, embryos or tissues)**<br>• Provide the name of the Institutional Animal Care and Use Committee (IACUC) or other relevant ethics board that reviewed the study protocol, and indicate whether they approved this research or granted a formal waiver of ethical approval<br>• Include an approval number if one was obtained<br>• If the study involved *non-human primates*, add *additional details* about animal welfare and steps taken to ameliorate suffering<br>• If anesthesia, euthanasia, or any kind of animal sacrifice is part of the study, include briefly which substances and/or methods were applied<br><br>**Field Research**<br><br>Include the following details if this study involves the collection of plant, animal, or other materials from a natural setting:<br>• Field permit number<br>• Name of the institution or relevant body that granted permission	
**Data Availability**<br><br>Authors are required to make the data underlying their research findings fully available, without restriction.	N/A - No results are reported

If the manuscript reports pilot or preliminary results, the data underlying those results must be made available at the time of publication. A Data Availability Statement describing where the data can be found is required at submission. Your answers to this question constitute the Data Availability Statement and will be published in the article, if accepted. When legal or ethical requirements prohibit public data sharing, authors must indicate how researchers may obtain access to the data. See the PLOS Data Policy and FAQ for detailed information.<br><br>**Important:** Stating 'data available on request from the author' is not sufficient. If your data are only available upon request, select 'No' for the first question and explain your situation in the text box.<br><br>For protocols without pilot or preliminary data, authors are strongly encouraged to state how they plan to share research data from their study when it is completed or published.<br><br>If your study includes pilot data, will they be made fully available without restriction?	
**Describe where the data may be found in full sentences. If you are copying our sample text, replace any instances of XXX with the appropriate details.**<br><br>• If **no pilot data are reported** in the manuscript, please enter the following: *No datasets were generated or analysed during the current study. All relevant data from this study will be made available upon study completion.*<br>• If the manuscript reports pilot data **held in a public repository**, include URLs, accession numbers or DOIs. If this information will only be available after acceptance, indicate this by ticking the box below. For example: *All XXX files*	No datasets were generated or analysed during the current study.

are available from the XXX database (accession number(s) XXX, XXX.).<br>• If the manuscript reports pilot data contained **within the manuscript and/or Supporting Information files**, enter the following:<br>*All relevant data are within the manuscript and its Supporting Information files.*<br>• If neither of these applies but you are able to provide **details of access elsewhere**, with or without limitations, please do so. For example:<br><br>*Data cannot be shared publicly because of [XXX]. Data are available from the XXX Institutional Data Access / Ethics Committee (contact via XXX) for researchers who meet the criteria for access to confidential data.*<br><br>*The data underlying the results presented in the study are available from (include the name of the third party and contact information or URL).*<br>• This text is appropriate if the data are owned by a third party and authors do not have permission to share the data.<br><br>For study protocols with data management or sharing plans, authors are encouraged to briefly describe in their Data Availability Statement how data they generate will be made accessible when the study is completed. For example:<br>• Deidentified research data will be made publicly available when the study is completed and published.<br><br>* typeset	
Additional data availability information:	

Amanda J Gupta
Yale University
School of Public Health
amanda.meyer@yale.edu
March 25, 2025

Dear Academic Editors,

I am pleased to submit our protocol manuscript titled “Methodologies for Studying Depression in Persons living with Tuberculosis: Protocol for a Scoping Review” for consideration for publication in PLOS ONE. This manuscript presents a scoping review protocol that aims to systematically map the methodologies employed in studying the intersection of tuberculosis (TB) and depression. Given the high prevalence of depression among persons with tuberculosis and diagnostic challenges posed by overlapping symptomatology, our study contributes to the scientific literature by identifying methodological gaps that may limit the development of effective diagnostic tools and treatment strategies.

This work is a protocol for a scoping review and is designed to provide a comprehensive overview of current research approaches. Our protocol employs the Levac et al framework, and incorporates the RE-AIM framework to systematically evaluate reach, effectiveness, adoption, implementation, and maintenance of methodologies reported in the literature. This approach will highlight opportunities to optimize research and public health interventions, ultimately improving outcomes for individuals with TB and depression.

We believe that this protocol will be of particular interest to readers and researchers in global health, epidemiology, mental health, and public health policy, as it offers valuable insights into the current state of research and highlights areas in need of methodological advancement. The findings from our scoping review are expected to guide future studies by providing a roadmap for integrating validated diagnostic measures and specialized treatment approaches into routine TB care.

To handle our manuscript, we suggest that Academic Editors with expertise in systematic reviews, global health, and mental health be considered. We recommend:

- Dr. Divya Bhandari (expertise in systematic reviews and global health)
- Dr. Esther Buregyeya (expertise in tuberculosis and epidemiology)
- Dr. Abel Fekadu Dadi, (expertise in depression, global health, and reviews)

Thank you for considering our protocol for publication in PLOS ONE. We look forward to your feedback and the opportunity to contribute to the scientific discourse in this important area.

Sincerely,

Amanda J Gupta
Research Associate II
Yale School of Public Health

Manuscript Click here to access/download;Manuscript;Manuscript.docx

Methodologies for studying depression in persons living with tuberculosis: Protocol for a scoping review

Amanda J Gupta[1,2], Patricia Turimumahoro[3], Lori Rosman[4], Jonathan E. Golub[5-7], David W Dowdy[5,6]

[1] Epidemiology of Microbial Diseases, Yale School of Public Health, New Haven, CT, USA

[2] Johns Hopkins Bloomberg School of Public Health, Maryland, USA

[3] World Alliance for Lung and Intensive Care Medicine in Uganda (WALIMU), Kampala, Uganda

[4] Welch Medical Library, School of Medicine, Johns Hopkins University, Baltimore, MD, USA

[5] Department of Epidemiology, Johns Hopkins Bloomberg School of Public Health, Baltimore, Maryland, USA

[6] Department of International Health, Johns Hopkins Bloomberg School of Public Health, Baltimore, Maryland, USA

[7] Center for Tuberculosis Research, Johns Hopkins University School of Medicine, Baltimore, Maryland, USA

*Corresponding Author

amanda.meyer@yale.edu (AJG)

Abstract

Tuberculosis (TB) and depression frequently co-occur, yet research has largely focused on prevalence rather than diagnostic or treatment methodologies. Given overlapping symptoms, robust research approaches are critical for improving detection and treatment strategies. This protocol defines a scoping review that aims to map methodologies used to study depression in individuals with TB, identifying gaps in research design, diagnosis, and treatment that may hinder clinical and public health advancements. A search was conducted in MEDLINE, Embase, PsycINFO, Global Health, Cochrane Library, and Africa-Wide Information using controlled vocabulary related to TB and depression. Studies examining the TB-depression relationship will be included. We will use Covidence to facilitate screening, selection, and data extraction. We will extract data on study design, diagnostic tools, treatment interventions, and analytical approaches. Descriptive characteristics of included studies will be presented using figures and tables. Using the RE-AIM framework, we will evaluate reach by assessing the extent to which studies explore both directions of the TB-depression relationship and include diverse, high-risk populations. Effectiveness will be examined by categorizing study designs to assess methodological diversity and evaluating the diagnostic tools and treatment interventions used, along with their reported efficacy. Adoption will be analyzed by identifying where and by whom depression diagnosis and treatment methodologies have been implemented and whether research findings have influenced clinical guidelines or public health policies. Implementation will be assessed by identifying barriers and facilitators reported in qualitative studies or by researchers regarding the integration of depression care into TB treatment settings. Finally, maintenance will be determined by examining whether diagnostic and treatment

methodologies were sustained within health systems, including long-term patient outcomes and the persistence of interventions beyond the research phase. By mapping existing methodologies and identifying research gaps, this review will provide valuable insights to guide future research study designs and improve diagnostic and treatment ~~strategies~~.

Introduction

Tuberculosis (TB), a communicable disease caused by *Mycobacterium tuberculosis,* is one of the leading infectious causes of mortality worldwide [1]. While it primarily affects the lungs (pulmonary TB), it can be found in other places throughout the body (extrapulmonary TB) such as in the lymph nodes, pleura, bone and joints, urogenital tract, and meninges to name a few. The COVID-19 pandemic accelerated deaths due to TB as less attention, and subsequently funding, has been paid to TB [1, 2]. This resulted in an estimated 10.8 million people developing new cases of active TB in 2023 with an estimated 1.25 million deaths due to TB disease [1]. Active TB refers to disease in which TB bacteria are replicating and causing clinical symptoms. Pulmonary forms of active TB are typically symptomatic and infectious while extrapulmonary TB is usually symptomatic but not transmissible. This contrasts with latent TB which is a state in which an individual is infected with TB but is generally asymptomatic and non-infectious. It is estimated that billions of individuals worldwide have latent TB. Furthermore, TB can be classified by drug resistance with ~400,000 PWTB having multidrug-resistant TB (resistance to at least isoniazid and rifampin) in 2023 [1]. Despite this large burden, TB is vastly underfunded leading to under-supported research endeavors and national TB programs left without appropriate funding to successfully combat the large TB burdens that they may face [1-5]. This results in gaps in TB diagnosis, treatment, and care in high TB burden settings [6-8].

One such gap in care is in the screening, diagnosis, and treatment of comorbidities in people with TB (PWTB) [9]. While the diagnosis and treatment protocols of infectious comorbidities of TB such as HIV have been extensively researched [10-13], the

diagnosis and treatment of comorbid non-communicable diseases (NCDs) such as mental health disorders have received little attention, despite their impacts on successful TB treatment [9, 14, 15]. Depression is one such overlooked comorbidity with potentially large impacts on PWTB's long-term health and wellbeing [16, 17]. Depression is a common mental health condition characterized by persistent sadness, loss of interest or pleasure, and functional impairment which can affect the overall quality of life of individuals suffering from it if not properly managed [18]. While clinically defined in various tools, such as the DSM-5, its expression and recognition vary across cultural and social contexts increasing the diagnostic and treatment complexity of the condition [19].

Depression appears to impact PWTB more frequently than the general population with one systematic review estimating the prevalence of depression amongst PWTB to be 45% [20]. Depression may not only be a consequence of TB, but also a potential risk factor due to its effects on immune function, health-seeking behavior, and treatment engagement [15, 21, 22]. Furthermore, TB-depression co-occurrence can be thought of as a syndemic, or an interacting set of conditions that cluster within social disadvantage, including intersecting biological, social, and structural factors, that results in an increased burden of disease [23-25]. For example, HIV infection, a highly prevalent co-morbidity in PWTB, contributes to immunosuppression, social stigma, and economic vulnerability, all of which heighten susceptibility to mental health disorders [20]. Additionally, the burden of depression may also vary by TB subtype —individuals with extrapulmonary TB may face diagnostic delays and uncertainty that heighten

psychological distress [26, 27], while those with multidrug-resistant TB (MDR-TB) often endure prolonged treatment, more severe side effects, greater economic uncertainty, and greater social isolation, all of which may increase the risk or severity of depression [28, 29].

Although this complex interplay is widely acknowledged, most PWTB are never screened for depression, let alone receive treatment for depression, due to a lack of providers, funding, cultural norms, and stigma [20, 22, 30, 31]. From a public health perspective, untreated depression contributes to delays in TB diagnosis and treatment initiation [30, 32], non-adherence to TB medications [33, 34], and ultimately increases negative TB outcomes such as loss to follow up and death [35, 36].

Despite the significant impact that depression may have on both health systems and PWTB, research appears to have been limited to prevalence surveys with few studies using longitudinal designs to investigate the complex intersection of these two diseases and even fewer exploring the methodologies used to screen, diagnose, or treat depression in PWTB. This may be in part due to diagnostic challenges posed by overlapping symptoms, such as TB-related fatigue, weight loss, and general malaise, all of which can masquerade as signs of depression, especially at the point of TB diagnosis, leading to misdiagnoses or missed diagnoses. Given this, a complex systems thinking approach [37-39] may be of use to researchers as it highlights the importance of moving beyond isolated risk factors to better consider the dynamic, bidirectional, and context-dependent interactions between TB and depression. This

perspective also underscores the need for diverse and multi-level methodological approaches that can better capture feedback loops (e.g. how depression worsens TB outcomes, which may in turn exacerbate depressive symptoms), variability across TB subtypes, and health system contexts. Furthermore, applying such a lens can help identify critical gaps in the current research landscape and more effectively guide future investigative efforts.

Given the substantial burden of depression among PWTB and the large focus on prevalence estimates within the literature with limited exploration of the methodological diversity and public health relevance of diagnostic and therapeutic approaches, a scoping review is particularly needed to broadly map the existing evidence, identify the most pressing knowledge gaps, and clarify how research has been conducted when studying these comorbid conditions. A scoping review would also help guide future studies by offering a roadmap for integrating effective and contextually appropriate mental health interventions into TB care. Therefore, we aim to conduct a scoping review to examine the methodologies used to study depression, including diagnostic and treatment methodologies, in PWTB, identifying research gaps and highlighting opportunities for improved methodological approaches in future public health research.

Methods

This section is presented in accordance with the scoping review framework by Arskey and O'Malley [40] and refined by Levac et al [41] which will guide the methodology of our scoping review. This framework consists of six steps consisting of 1) Identifying the

research question 2) Identifying relevant studies 3) Study Selection 4) Charting the data 5) Collating, summarizing, and reporting the results and 6) Consultation with stakeholders on study findings.

Identifying the research question

The research question for our review is: what methodologies have been used in the research literature to study depression (including diagnosis and treatment) among individuals with tuberculosis, and what are the gaps in methodological approaches?

Identification of relevant studies

Table 1 includes the eligibility criteria for this scoping review. We will search the following electronic databases: MEDLINE, Embase, Global Health, the Cochrane Library, the WHO Regional Libraries, Africa-Wide Information, and PsycINFO. These databases were selected for their relevance to the scope of this review: MEDLINE, Embase, Global Health, and the Cochrane Library are widely used for biomedical and public health research; the WHO Regional Libraries and Africa-Wide Information are included to ensure geographic representation, particularly from high TB burden regions such as sub-Saharan Africa; and PsycINFO is included for its comprehensive coverage of psychological and mental health research. The preliminary search strategy, constructed by a trained medical librarian (LR) after several weeks of testing various combinations of terms to ensure maximum reach, focused on combinations of the following areas: terms related to “tuberculosis” and terms related to “depression”.

Appropriate MeSH terms were utilized for each of the proposed databases (S1 Appendix).

Given the anticipated paucity of literature, we did not include date restrictions but rather allowed any study to be included that was published prior to the search date. Finally, the electronic database search will be supplemented by manual searches, including the screening of reference lists from all included studies to identify any additional relevant articles that may have been missed.

Table 1. Study Eligibility Criteria

**Domain**	**Included**	**Excluded**
Participants	Individuals with tuberculosis regardless of age, sex, socioeconomic status and type including:<br>• Microbiologically confirmed<br>• Clinically diagnosed<br>• Pulmonary<br>• Extra-pulmonary<br>• Latent<br>• Active<br>• Drug-resistant<br>• Drug-susceptible	• Those without a confirmed tuberculosis diagnosis<br>• Animal participants<br>• Participants with nontuberculous mycobacterial infections (e.g. leprosy)<br>• Studies that only include laboratory samples and isolates
Concept or intervention	Studies that contain information on depression and tuberculosis including:<br>• Diagnostic tools, methods, and protocols<br>• Therapeutic interventions (pharmacological or psychological)<br>• Intersection of tuberculosis and depression (studies addressing risk, etc.)	• Studies that only include tuberculosis or depression independently without exploring their co-occurrence<br>• Those that only focus on biological mechanisms of tuberculosis and/or depression
Outcome	• Sensitivity, specificity, and/or accuracy of diagnostic tools for depression in TB patients<br>• Studies focused on the identification of diagnostic challenges, barriers, or facilitators<br>• Validation of relevant diagnostic measures	Outcomes not explicitly linked to the study of depression in TB populations including:<br>• Studies reporting biochemical, genetic, or molecular outcomes without clinical or diagnostic relevance

	• Changes in depression symptoms (including ~~patient~~-reported ~~outcomes~~) during TB treatment<br>• TB treatment adherence, completion, and/or complication ~~rates~~ because of depression treatment or lack thereof<br>• ~~Studies with outcomes on methodological challenges~~ in studying depression and tuberculosis together	• ~~Those with general~~ TB treatment outcomes unrelated to or ~~without~~ considering depression<br>• ~~Those~~ with insufficient detail on how outcomes were measured or analyzed<br>• ~~Those~~ based solely on opinions or informal observations
Context	Research conducted in healthcare or community settings of relevance such as<br>• Tuberculosis clinics<br>• Hospital settings<br>• Primary care clinics<br>• Community health programs<br>• Any other relevant setting where tuberculosis and depression services may be provided<br>Global geographic inclusion	Research from unrelated contexts such as veterinary or laboratory studies~~.~~<br><br>~~No exclusion criteria based on geography~~
~~Language~~	English	Language other than English
Study Design	• Primary studies<br>• Randomized controlled trials<br>• Prevalence surveys<br>• Case studies<br>• Cohort studies<br>• Case-control studies<br>• Systematic reviews<br>• Meta-analyses<br>• Scoping reviews<br>• Qualitative studies<br>• Mixed methods analyses<br>• Diagnostic test accuracy studies	• Meta-reviews (reviews of reviews)<br>• Opinion pieces, editorials, and letters to the editor that do not contain empirical data<br>• ~~Abstracts without full-text availability~~<br>• Protocol papers

Study selection

We ~~will~~ use Covidence to screen study titles, abstracts and full text articles for inclusion. Two reviewers (AJG, PT) will review abstracts in parallel for inclusion. To minimize ~~bias~~, ~~reviewers will be blinded to other~~ reviewers' ratings for screening ~~except~~ when resolving conflicts. Reviewers will meet after every 20 abstracts to resolve any conflicts and to propose/implement updates to the search strategy or inclusion criteria. All abstracts that

pass screening will have the full text reviewed by the two reviewers, again, in parallel for final inclusion in the review. Disagreements will be resolved via discussion and, if needed, by a third reviewer (DWD).

Charting the data

After piloting the data extraction tool, we will extract all data using Covidence. We will extract pertinent study details such as authors, location of publication/journal name, year of publication, research question addressed, study design, geographic context, date when study was conducted, study location (community, health facility, etc.), sample size, participants characteristics (such as age, HIV status, sex, socioeconomic status, other mental health disorders, etc.), screening and diagnostic tools used for depression, treatment intervention approach utilized for depression (e.g. psychological approaches like cognitive-behavioral therapy, pharmacological like antidepressants, and/or alternative interventions like mindfulness techniques or yoga), treatment intervention components outcomes assessed, treatment intervention mode of delivery (e.g. in-person therapy, oral medication etc.), duration and frequency of treatment intervention, statistical methods used (e.g. regression analysis, chi-square, multi-level models etc.), comparison population (if a control or other comparator used), outcome measures (e.g, depression prevalence estimates, odds ratios etc.), measures of precision (e.g. 95% CI), statistical significance, and, for qualitative studies, key themes identified, barriers and facilitators to diagnosis and/or treatment identified, and the framework or content analysis utilized (i.e. thematic analysis, grounded theory, case study approach, etc.). Additionally, if the authors identify any limitations relevant to the research question, such

as methodological limitations, we will extract this data. One author (AJG) will extract the data with a second reviewer (PT) checking extracted data for accuracy. These two authors will routinely meet during the data extraction phase to ensure the extraction tool is capturing all relevant data needed to answer the overall research question.

~~Results~~

Collating, summarizing, and reporting results

We will provide descriptive characteristics of all studies included in the review using figures and tables to fully summarize the studies. While ~~our focus in the present research is to characterize the current state of the literature with inclusion not dependent on literature quality,~~ we will conduct a quality appraisal ~~of~~ each study ~~as~~ the presence or absence of high-quality research is relevant to understanding ~~research~~ gaps. As ~~there~~ will be many different study designs, to assess quality, we will use the Jadad scale for randomized controlled trials, the Newcastle-Ottawa scale for observational studies, and the Joanna Briggs Institute Critical Appraisal Tool for Qualitative Research ~~for qualitative studies~~.

We will structure the reporting of our results using the RE-AIM (Reach, Effectiveness, Adoption, Implementation and Maintenance) framework [42]. As we are interested in the way these two diseases have been studied together, for *reach*, we will determine the extent to which studies have explored both directions of the TB-depression relationship. We will also determine the reach of researchers in terms of including representative samples of PWTB, including diverse populations and healthcare settings, and their

inclusion of high-risk groups (such as those with HIV, drug-resistant TB etc.). For *effectiveness* we will categorize the study designs used to assess the diversity of methodological approaches as a wide range of designs – such as cross-sectional, longitudinal and randomized controlled trials – is essential for fully elucidating the co-occurrence of TB and depression. A lack of diverse study designs may indicate gaps in the field's ability to fully understand and address this relationship. Additionally, we will determine the types of diagnostic measures used for depression along with their effectiveness (if reported) as well as how efficacious or effective any treatment interventions were at altering either depression or TB related outcomes. For *adoption* we will look at the extent to which depression screening, diagnosis, and treatment methodologies are being adopted in different settings (such as by national programs, referral centers, local clinics, etc) and by whom (e.g. physicians, nurses, community health works etc.) and examine the way in which research findings on these two diseases have impacted clinical guidelines and public health policies if mentioned by study authors. For *implementation*, we will determine barriers and facilitators identified to the implementation of depression related care in real-world TB treatment settings. Finally, for *maintenance* we will determine whether any depression related methodologies for diagnosis or treatment were sustained or embedded within existing health systems. This will include, if reported upon, long-term patient outcomes, if methodologies persisted beyond the research phase, and if any studies evaluated how health systems integrated and sustained any proposed diagnostic or treatment interventions.

Consultation with Stakeholders

We will include a consultation phase to enhance the relevance and applicability of our findings. We will engage a diverse group of stakeholders, drawing on global collaborators with expertise in TB and mental health from an established network of the research team, as well as individuals with lived experience of TB, including ~~patients~~ from Uganda who are participating in an unrelated, ongoing cohort study. These consultations will help provide contextual insights into the interpretation of findings and identify additional knowledge gaps or implementation considerations that may not be fully captured in the published literature. Input from public health practitioners and researchers working in high TB burden settings will also be sought to ensure the review's implications are both meaningful and actionable.

Data Sharing

In line with open science principles, we will make the final data extraction sheet and study metadata openly available through a public repository such as the Open Science Framework upon completion of the review.

Timeline

The initial search was conducted on March 31, 2025. All screening, including title and abstract screening and full text screening ~~will take~~ place from April to June 2025. Data extraction will take place in July 2025. Analysis and synthesis of results will take place from July to August 2025 with final results expected by September 2025.

Discussion

This scoping review will explore the existing literature on the intersection between depression and TB allowing us to identify research gaps, including in the diagnosis and treatment of depression, amongst PWTB. Importantly, this will allow us to understand any methodological gaps that exist and approaches that researchers have historically taken to tackle these two diseases concurrently.

By mapping the existing methodologies used to study depression in PWTB, this review will identify gaps in research design, highlight areas where more rigorous and diverse study approaches are needed, and provide insights into potential pathways for improving integrated care. Findings from this review will contribute to advancing research beyond cross-sectional prevalence surveys by encouraging the use of longitudinal, interventional, and mixed-methods studies that can better inform evidence-based practice. Furthermore, this review ~~will~~ underscore ~~the~~ urgent need for validated diagnostic tools and specialized treatment approaches to address the overlapping symptoms and complex interplay between tuberculosis and depression, ultimately supporting the development of more effective strategies to guide future research and public health interventions. ~~Additionally, our review will be methodologically rigorous by using the~~ Levac et al framework to ensure a systematic and transparent approach to study identification and selection. This will be enhanced through the use of RE-AIM to synthesize results, providing a structured lens to assess how methodological approaches to researching TB and depression have created knowledge ~~gaps~~ in studying the diseases concurrently. Finally, the planned stakeholder consultations will add depth and practical relevance to the final interpretation of the findings ~~ensuring that~~

the review will be both methodologically rigorous and highly relevant to global TB and mental health research practices.

Our review will have some limitations that are worth discussing a priori. First, we have limited the search to English language studies. Given the burden of tuberculosis in many non-English speaking countries such as China where many publications are in Mandarin, this may exclude several studies that would otherwise be eligible for this scoping review. Second, we are beginning this search with the term depression, a Western term, and its associated synonyms. Many countries use alternate terms for depression, such as shenjing shuairuo (nervous weakness) in China or yuutsu in Japan, meaning that our initial search may not fully capture the available literature. Given the long list of terms that could possibly be used by different countries, it is not likely we will be able to capture all possible terms and subsequently may miss eligible publications. Third, since the primary aim is to describe and categorize existing research rather than synthesize findings or estimate effect sizes, scoping reviews cannot make definitive conclusions about intervention effectiveness or causality. However, depending on the results of the current review, it may be appropriate to conduct a subsequent systematic review and meta-analysis to assess the public health impact of depression-related interventions amongst PWTB. Finally, as with all scoping reviews, the breadth of study types and topics can make data synthesis more complex than in a systematic review. Given our aim of establishing methodologies used in the field, a broad inclusion is necessary but could provide more narrative results. We believe our use of RE-AIM to structure the results, regardless of study type, will aid in creating meaningful outputs.

Conclusions

This scoping review will advance understanding of how depression has been studied in the context of tuberculosis by systematically mapping existing methodologies and identifying critical research gaps to guide future public health research and intervention development.

Acknowledgements

We would like to thank both Yale University and Johns Hopkins University for providing institutional support for this work.

Author Contributions

AJG conceptualized the research question and wrote the initial draft of this protocol. PT, JEG, and DWD provided substantial contributions to the conception and design of the protocol including reviewing it for intellectual merit and refinement of the research question. LR created the initial search queries and ran the initial search. All authors reviewed and approved the final manuscript.

References

1. World Health Organization. Global Tuberculosis Report 2024. Geneva; 2024 29 October 2024.https://www.who.int/teams/global-programme-on-tuberculosis-and-lung-health/tb-reports/global-tuberculosis-report-2024
2. World Health Organization. Global Tuberculosis Report 2022. Geneva, Switzerland: World Health Organization; 2022. https://www.iom.int/sites/g/files/tmzbdl486/files/documents/2023-03/Global-TB-Report-2022.pdf
3. Chaisson RE, Frick M, Nahid P. The scientific response to TB - the other deadly global health emergency. Int J Tuberc Lung Dis. 2022;26(3):186-9.
4. Venkatesan P. Worrying lack of funding for tuberculosis. Lancet Infect Dis. 2022;22(3):318.
5. Treatment Action Group. Tuberculosis Research Funding Trends 2005-2023. New York, New York; 2024 13 December, 2024.https://www.treatmentactiongroup.org/resources/tbrd-report/tbrd-report-2024/
6. Armstrong-Hough M, Turimumahoro P, Meyer AJ, Ochom E, Babirye D, Ayakaka I, et al. Drop-out from the tuberculosis contact investigation cascade in a routine public health setting in urban Uganda: A prospective, multi-center study. PLoS One. 2017;12(11):e0187145.
7. Kim J, Keshavjee S, Atun R. Health systems performance in managing tuberculosis: analysis of tuberculosis care cascades among high-burden and non-high-burden countries. J Glob Health. 2019;9(1):010423.

8. Subbaraman R, Jhaveri T, Nathavitharana RR. Closing gaps in the tuberculosis care cascade: an action-oriented research agenda. Journal of Clinical Tuberculosis and Other Mycobacterial Diseases. 2020;19:100144. https://www.sciencedirect.com/science/article/pii/S2405579419300683

9. Jarde A, Romano E, Afaq S, Elsony A, Lin Y, Huque R, et al. Prevalence and risks of tuberculosis multimorbidity in low-income and middle-income countries: a meta-review. BMJ Open. 2022;12(9):e060906. http://bmjopen.bmj.com/content/12/9/e060906.abstract

10. Duarte R, Lönnroth K, Carvalho C, Lima F, Carvalho ACC, Muñoz-Torrico M, et al. Tuberculosis, social determinants and co-morbidities (including HIV). Pulmonology. 2018;24(2):115-9.

11. Bell LCK, Noursadeghi M. Pathogenesis of HIV-1 and Mycobacterium tuberculosis co-infection. Nat Rev Microbiol. 2018;16(2):80-90.

12. Méndez-Samperio P. Diagnosis of Tuberculosis in HIV Co-infected Individuals: Current Status, Challenges and Opportunities for the Future. Scand J Immunol. 2017;86(2):76-82.

13. Dorman SE. Diagnosis of HIV-associated tuberculosis. Curr Opin HIV AIDS. 2018;13(6):462-8.

14. Galea JT, Monedero-Recuero I, Sweetland AC. Beyond screening: a call for the routine integration of mental health care with tuberculosis treatment. Public Health Action. 2019;9(1):2.

15. Cáceres G, Calderon R, Ugarte-Gil C. Tuberculosis and comorbidities: treatment challenges in patients with comorbid diabetes mellitus and depression. Therapeutic

Advances in Infectious Disease. 2022;9:20499361221095831. https://doi.org/10.1177/20499361221095831

16. Nightingale R, Carlin F, Meghji J, McMullen K, Evans D, van der Zalm MM, et al. Post-TB health and wellbeing. Int J Tuberc Lung Dis. 2023;27(4):248-83.

17. Chandra M, Rana P, Chandra K, Arora VK. Tuberculosis - Depression syndemic: A public health challenge. Indian J Tuberc. 2019;66(1):197-202.

18. Simon GE, Moise N, Mohr DC. Management of Depression in Adults: A Review. JAMA. 2024;332(2):141-52. https://doi.org/10.1001/jama.2024.5756

19. Haroz EE, Ritchey M, Bass JK, Kohrt BA, Augustinavicius J, Michalopoulos L, et al. How is depression experienced around the world? A systematic review of qualitative literature. Social Science & Medicine. 2017;183:151-62. https://www.sciencedirect.com/science/article/pii/S0277953616307109

20. Duko B, Bedaso A, Ayano G. The prevalence of depression among patients with tuberculosis: a systematic review and meta-analysis. Annals of General Psychiatry. 2020;19(1):30. https://doi.org/10.1186/s12991-020-00281-8

21. Cheng KC, Liao KF, Lin CL, Lai SW. Increased Risk of Pulmonary Tuberculosis in Patients with Depression: A Cohort Study in Taiwan. Front Psychiatry. 2017;8:235.

22. Hayward SE, Deal A, Rustage K, Nellums LB, Sweetland AC, Boccia D, et al. The relationship between mental health and risk of active tuberculosis: a systematic review. BMJ Open. 2022;12(1):e048945.

23. Verma AK, Singh A. Psychological disorders in tuberculosis: A narrative review. Indian Journal of Tuberculosis. 2024;71:S162-S4. https://www.sciencedirect.com/science/article/pii/S0019570724001641

24. Sweetland AC, Kritski A, Oquendo MA, Sublette ME, Norcini Pala A, Silva LRB, et al. Addressing the tuberculosis-depression syndemic to end the tuberculosis epidemic. Int J Tuberc Lung Dis. 2017;21(8):852-61.

25. Van Der Walt M, Keddy KH. The Tuberculosis-Depression Syndemic and Evolution of Pharmaceutical Therapeutics: From Ancient Times to the Future. Front Psychiatry. 2021;12:617751.

26. Wali A, Safdar N, Ambreen A, Hassan S, Yaqoob A, Mustafa T. Effects of extrapulmonary TB on patient quality of life and recurrence. Public Health Action. 2024;14(3):112-8.

27. Anye LC, Agbortabot Bissong ME, Njundah AL, Siewe Fodjo JN. Depression, anxiety and medication adherence among tuberculosis patients attending treatment centres in Fako Division, Cameroon: cross-sectional study. BJPsych Open. 2023;9(3):e65. https://www.cambridge.org/core/product/E5396B35E949366009834726AB74B8F0

28. Tomita A, Ramlall S, Naidu T, Mthembu SS, Padayatchi N, Burns JK. Major depression and household food insecurity among individuals with multidrug-resistant tuberculosis (MDR-TB) in South Africa. Social Psychiatry and Psychiatric Epidemiology. 2019;54(3):387-93. https://doi.org/10.1007/s00127-019-01669-y

29. Murugan Y, Patel N, Kumar V, Gandhi R. Mental Health Impacts of Multidrug-Resistant Tuberculosis in Patients and Household Contacts: A Mixed Methods Study. Cureus. 2024;16(5):e60412.

30. Dos Santos AP, Lazzari TK, Silva DR. Health-Related Quality of Life, Depression and Anxiety in Hospitalized Patients with Tuberculosis. Tuberc Respir Dis (Seoul). 2017;80(1):69-76.

31. Rathod S, Pinninti N, Irfan M, Gorczynski P, Rathod P, Gega L, et al. Mental Health Service Provision in Low- and Middle-Income Countries. Health Services Insights. 2017;10:1178632917694350. https://journals.sagepub.com/doi/abs/10.1177/1178632917694350

32. Molebatsi K, Wang Q, Dima M, Ho-Foster A, Modongo C, Zetola N, et al. Depression and delayed tuberculosis treatment initiation among newly diagnosed patients in Botswana. Global Public Health. 2021;16(7):1088-98. https://doi.org/10.1080/17441692.2020.1826049

33. Theron G, Peter J, Zijenah L, Chanda D, Mangu C, Clowes P, et al. Psychological distress and its relationship with non-adherence to TB treatment: a multicentre study. BMC Infect Dis. 2015;15:253.

34. Yan S, Zhang S, Tong Y, Yin X, Lu Z, Gong Y. Nonadherence to Antituberculosis Medications: The Impact of Stigma and Depressive Symptoms. Am J Trop Med Hyg. 2018;98(1):262-5.

35. Ruiz-Grosso P, Cachay R, de la Flor A, Schwalb A, Ugarte-Gil C. Association between tuberculosis and depression on negative outcomes of tuberculosis treatment: A systematic review and meta-analysis. PLOS ONE. 2020;15(1):e0227472. https://doi.org/10.1371/journal.pone.0227472

36. Ambaw F, Mayston R, Hanlon C, Medhin G, Alem A. Untreated depression and tuberculosis treatment outcomes, quality of life and disability, Ethiopia. Bull World Health Organ. 2018;96(4):243-55.

37. Fried EI, Robinaugh DJ. Systems all the way down: embracing complexity in mental health research. BMC Medicine. 2020;18(1):205. https://doi.org/10.1186/s12916-020-01668-w

38. Scheffer M, Bockting CL, Borsboom D, Cools R, Delecroix C, Hartmann JA, et al. A Dynamical Systems View of Psychiatric Disorders-Theory: A Review. JAMA Psychiatry. 2024;81(6):618-23.

39. Westhoff M, Berg M, Reif A, Rief W, Hofmann SG. Major Problems in Clinical Psychological Science and How to Address them. Introducing a Multimodal Dynamical Network Approach. Cognitive Therapy and Research. 2024;48(5):791-807. https://doi.org/10.1007/s10608-024-10487-9

40. Arksey H, O'Malley L. Scoping studies: towards a methodological framework. International Journal of Social Research Methodology. 2005;8(1):19-32. https://doi.org/10.1080/1364557032000119616

41. Levac D, Colquhoun H, O'Brien KK. Scoping studies: advancing the methodology. Implement Sci. 2010;5:69.

42. Glasgow RE, Vogt TM, Boles SM. Evaluating the public health impact of health promotion interventions: the RE-AIM framework. Am J Public Health. 1999;89(9):1322-7.

S1 Appendix: Initial Search terms by database

S1 Appendix Initial Search Criteria

Click here to access/download
Supporting Information
S1 Appendix.docx

PRISMA-P Checklist

Click here to access/download
Supporting Information
PRISMA-P-checklist.docx

~~Title:~~ Methodologies for ~~Studying~~ studying ~~Depression~~ depression in ~~Persons~~ persons living with ~~Tuberculosis~~tuberculosis: Protocol for a ~~Scoping~~ scoping ~~Review~~review

Formatted: Centered

~~Authors:~~ Amanda J Gupta[1,2], Patricia Turimumahoro[3], Lori Rosman[4], Jonathan E. Golub[5-7], David W Dowdy[5,6]

~~1.~~ 1 Epidemiology of Microbial Diseases, Yale School of Public Health, New Haven, CT, USA

~~2.~~ 2 Johns Hopkins Bloomberg School of Public Health, Maryland, USA

~~3.~~ 3 World Alliance for Lung and Intensive Care Medicine in Uganda (WALIMU), Kampala, Uganda

~~4.~~ 4 Welch Medical Library, School of Medicine, Johns Hopkins University, Baltimore, MD, USA

~~5.~~ 5 Department of Epidemiology, Johns Hopkins Bloomberg School of Public Health, Baltimore, Maryland, USA

~~6.~~ 6 Department of International Health, Johns Hopkins Bloomberg School of Public Health, Baltimore, Maryland, USA

~~7.~~ 7 Center for Tuberculosis Research, Johns Hopkins University School of Medicine, Baltimore, Maryland, USA

Formatted: Normal, No bullets or numbering

Formatted: Not Superscript/ Subscript

Formatted: Not Superscript/ Subscript

Formatted: Not Superscript/ Subscript

Formatted: Not Superscript/ Subscript

Formatted: Not Superscript/ Subscript

~~Keywords: tuberculosis; depression; global health; reviews~~

*Corresponding Author

amanda.meyer@yale.edu (AJG)

Field Code Changed

Abstract ~~(296 words):~~

Tuberculosis (TB) and depression frequently co-occur, yet research has largely focused on prevalence rather than diagnostic or treatment methodologies. Given overlapping symptoms, robust research approaches are critical for improving detection and treatment strategies. This ~~scoping review~~ protocol defines a scoping review that aims to map methodologies used to study depression in individuals with TB, identifying gaps in research design, diagnosis, and treatment that may hinder clinical and public health advancements. A search was conducted in MEDLINE, Embase, PsycINFO, Global Health, Cochrane Library, and Africa-Wide Information using controlled vocabulary related to TB and depression. Studies examining the TB-depression relationship will be included. We will use Covidence to facilitate screening, selection, and data extraction. We will extract data on study design, diagnostic tools, treatment interventions, and analytical approaches. Descriptive characteristics of included studies will be presented using figures and tables. Using the RE-AIM framework, we will evaluate reach by assessing the extent to which studies explore both directions of the TB-depression relationship and include diverse, high-risk populations. Effectiveness will be examined by categorizing study designs to assess methodological diversity and evaluating the diagnostic tools and treatment interventions used, along with their reported efficacy. Adoption will be analyzed by identifying where and by whom depression diagnosis and treatment methodologies have been implemented and whether research findings have influenced clinical guidelines or public health policies. Implementation will be assessed by identifying barriers and facilitators reported in qualitative studies or by researchers regarding the integration of depression care into TB treatment settings. Finally, maintenance will be determined by examining whether diagnostic and treatment

Formatted: Font: 18 pt, Bold, Font color: Text 1

Formatted: Heading 1

methodologies were sustained within health systems, including long-term patient outcomes and the persistence of interventions beyond the research phase. By mapping existing methodologies and identifying research gaps, this review will provide valuable insights to guide future research study designs and improve diagnostic and treatment strategies.

Introduction

Tuberculosis (TB), a communicable disease caused by *Mycobacterium tuberculosis*, is one of the leading infectious causes of mortality killers worldwide (1). While it primarily affects the lungs (pulmonary TB), it can be found in other places throughout the body (extrapulmonary TB) such as in the lymph nodes, pleura, bone and joints, urogenital tract, and meninges to name a few. The COVID-19 pandemic accelerated these deaths due to TB as less attention, and subsequently funding, has been paid to TB (1, 2). This resulted in an estimated 10.8 million people developing new cases of active TB in 2023 with an estimated 1.25 million deaths due to TB disease (1). Active TB refers to disease in which TB bacteria are replicating and causing clinical symptoms. Pulmonary forms of active TB are typically symptomatic and infectious while extrapulmonary TB is usually symptomatic but not transmissible. This contrasts with latent TB which is a state in which an individual is infected with TB but is generally asymptomatic and non-infectious. It is estimated that billions of individuals worldwide have latent TB. Furthermore, TB can be classified by drug resistance with ~400,000 PWTB having multidrug-resistant TB (resistance to at least isoniazid and rifampin) in 2023 (1). Despite this large burden, TB is vastly underfunded leading to under-supported research endeavors and national TB programs left without appropriate funding to successfully combat the large TB burdens that they may face (1-5). This results in gaps in TB diagnosis, treatment, and care in high TB burden settings (6-8).

One such gap in care is in the screening, diagnosis, and treatment of comorbidities in people with TB (PWTB) (9). While certain communicable comorbidities (such as TB diagnosis and treatment in those living with HIVWhile the diagnosis and treatment

Formatted: Font: 18 pt, Font color: Text 1

Formatted: Heading 1, Line spacing: single

protocols of infectious comorbidities of TB such as HIV have been extensively researched (10-13),~~)~~ the diagnosis and treatment of comorbid ~~have been extensively researched,~~ non-communicable ~~comorbidities~~ diseases (NCDs) ~~– and especially~~ such as mental health ~~comorbidities~~ disorders ~~–~~ have received little attention, despite their impacts on successful TB treatment (9, 14, 15). Depression is one such overlooked comorbidity with potentially large impacts on PWTB's long-term health and wellbeing (16, 17). Depression is a common mental health condition characterized by persistent sadness, loss of interest or pleasure, and functional impairment which can affect the overall quality of life of individuals suffering from it if not properly managed (18). While clinically defined in various tools, such as the DSM-5, its expression and recognition vary across cultural and social contexts increasing the diagnostic and treatment complexity of the condition (19).

Depression appears to impact PWTB more frequently than the general population with one systematic review estimating the prevalence of depression amongst PWTB to be ~~a staggering~~ 45% (20). ~~In fact, depression may even be a risk factor for developing TB~~Depression may not only be a consequence of TB, but also a potential risk factor due to its effects on immune function, health-seeking behavior, and treatment engagement (15, 21, 22). Furthermore, TB-depression co-occurrence can be thought of as a syndemic, or an interacting set of conditions that cluster within social disadvantage, including intersecting biological, social, and structural factors, that results in an increased burden of disease (23-25). For example, HIV infection, a highly prevalent co-morbidity in PWTB, contributes to immunosuppression, social stigma, and economic

vulnerability, all of which heighten susceptibility to mental health disorders (20). Additionally, the burden of depression may also vary by TB subtype —individuals with extrapulmonary TB may face diagnostic delays and uncertainty that heighten psychological distress (26, 27), while those with multidrug-resistant TB (MDR-TB) often endure prolonged treatment, more severe side effects, greater economic uncertainty, and greater social isolation, all of which may increase the risk or severity of depression (28, 29).

Although this ~~is the case~~complex interplay is widely acknowledged, most PWTB are never screened for depression, let alone receive treatment for ~~their~~ depression, due to a lack of providers, funding, cultural norms, and stigma (20, 22, 30, 31). From a public health perspective, ~~Without treatment,~~untreated depression ~~increases the chances that a PWTB delays seeking and initiating treatmen~~contributes to delays in TB diagnosis and treatment initiation (30, 32),~~ is~~ non-adherenc~~e~~t to TB medications ~~once they are diagnosed~~ (33, 34), and ultimately increases ~~their chances for~~ negative TB outcomes such as loss to follow up and death (35, 36).

Despite the ~~large burden~~significant impact that depression ~~poses~~ may have on ~~for~~ both health systems and PWTB, research appears to have been limited to prevalence surveys with few studies using longitudinal designs to investigate the complex intersection of these two diseases and even fewer exploring the methodologies used to screen, diagnose, or treat depression in PWTB. This may be in part due to diagnostic challenges posed by overlapping symptoms, such as TB-related fatigue, weight loss,

and general malaise, all of which can masquerade as signs of depression, especially at the point of TB diagnosis, leading to misdiagnoses or missed diagnoses. Given this, a complex systems thinking approach (37-39) may be of use to researchers as it highlights the importance of moving beyond isolated risk factors to better consider the dynamic, bidirectional, and context-dependent interactions between TB and depression. This perspective also underscores the need for diverse and multi-level methodological approaches that can better capture feedback loops (e.g. how depression worsens TB outcomes, which may in turn exacerbate depressive symptoms), variability across TB subtypes, and health system contexts. Furthermore, applying such a lens can help identify critical gaps in the current research landscape and more effectively guide future investigative efforts.

Given the substantial burden of depression among PWTB and the large focus on prevalence estimates within the literature with limited exploration of the methodological diversity and public health relevance of diagnostic and therapeutic approaches, a scoping review is particularly needed to broadly map the existing evidence, identify the most pressing knowledge gaps, and clarify how research has been conducted when studying these comorbid conditions. A scoping review would also help guide future studies by offering a roadmap for integrating effective and contextually appropriate mental health interventions into TB care. Therefore, we aim to conduct a scoping review to examine the methodologies used to study depression, including diagnostic and treatment methodologies, in PWTB, identifying research gaps and highlighting opportunities for improved methodological approaches in future public health research.

Methods

We will utilize the framework for conducting scoping studies originally developedThis section is presented in accordance with the scoping review framework by Arskey and O'Malley (40) and updated refined by Levac et al (41) which will guide the methodology of our scoping review. This framework consists of six steps consisting of 1) Identifying the research question 2) Identifying relevant studies 3) Study Selection 4) Charting the data 5) Collating, summarizing, and reporting the results and 6) Consultation with stakeholders on study findings.

Formatted: Font: 18 pt, Bold, Font color: Text 1

Formatted: Heading 1, Line spacing: single

Identifying the Research research Questionquestion

The research question for our review is: what methodologies have been used in the research literature to study depression (including diagnosis and treatment) among individuals with tuberculosis, and what are the gaps in methodological approaches? We will screen studies that have sought to examine the relationship between TB and depression.

Formatted: Font: Bold, Font color: Text 1

Formatted: Heading 2, Line spacing: single

Formatted: Font: Bold, Font color: Text 1

Formatted: Font: Bold, Font color: Text 1

Identification of rRelevant Studiesstudies

Table 1 includes the eligibility criteria for this scoping review. We will search the following electronic databases: MEDLINE, Embase, Global Health, the Cochrane Library, the WHO Regional Libraries, Africa-Wide Information, and PsycINFO. These databases were selected for their relevance to the scope of this review: MEDLINE, Embase, Global Health, and the Cochrane Library are widely used for biomedical and

Formatted: Font: Bold, Font color: Text 1

Formatted: Heading 2, Line spacing: single

Formatted: Font: Bold, Font color: Text 1

Formatted: Font: Bold, Font color: Text 1

public health research; the WHO Regional Libraries and Africa-Wide Information are included to ensure geographic representation, particularly from high TB burden regions such as sub-Saharan Africa; and PsycINFO is included for its comprehensive coverage of psychological and mental health research. ~~We will search MEDLINE, Embase, The WHO Regional Libraries, Global Health, and The Cochrane Library due to their near-ubiquitous use for biomedical and healthcare research along with Africa-Wide Information due to the high burden of TB in Africa, and PsychInfo for its specialization in psychology.~~ The preliminary search strategy, constructed by a trained medical librarian (LR) after several weeks of testing various combinations of terms to ensure maximum reach, focused on combinations of the following areas: terms related to “tuberculosis” and terms related to “depression”. Appropriate MeSH terms were utilized for each of the proposed databases (S1 Appendix).

Given the anticipated paucity of literature, we ~~will~~ did not include date restrictions but rather allowed any study to be included that was published prior to the search date. Finally, the electronic database search will be supplemented by manual searches, including the screening of reference lists from all included studies to identify any additional relevant articles that may have been missed.

Table 1. Study Eligibility Criteria

Formatted: Font: Bold

Domain	Included	Excluded
Participants	Individuals with tuberculosis regardless of age, sex, socioeconomic status and type including:<br>• Microbiologically confirmed<br>• Clinically diagnosed<br>• Pulmonary<br>• Extra-pulmonary<br>• Latent<br>• Active<br>• Drug-resistant	• Those without a confirmed tuberculosis diagnosis<br>• ~~Studies involving animals~~ Animal participants<br>• ~~Studies involving~~Participants with nontuberculous mycobacterial infections ~~such as~~(e.g. leprosy)<br>• Studies that only includ~~eing only~~ laboratory samples and isolates

<table>
<tr><td></td><td>Drug-susceptible</td><td></td></tr>
<tr><td>Concept or intervention</td><td>Studies that contain information on depression and tuberculosis including:
Diagnostic tools, methods, and protocols
Therapeutic interventions (pharmacological or psychological)
Intersection of tuberculosis and depression (studies addressing risk, etc.)</td><td>Studies that only include tuberculosis or depression independently without exploring their co-occurrence
Those that only focus on biological mechanisms of tuberculosis and/or depression</td></tr>
<tr><td>Outcome</td><td>Sensitivity, specificity, and/or accuracy of diagnostic tools for depression in TB patients
Studies focused on the identification of diagnostic challenges, barriers, or facilitators
Validation of relevant diagnostic measures
Changes in depression symptoms (including patient-reported outcomes) during TB treatment
TB treatment adherence, completion, and/or complication rates because of depression treatment or lack thereof
Studies with outcomes on methodological challenges in studying depression and tuberculosis together</td><td>Outcomes not explicitly linked to the study of depression in TB populations including:
Studies reporting biochemical, genetic, or molecular outcomes without clinical or diagnostic relevance
Those with general TB treatment outcomes unrelated to or without considering depression
Those with insufficient detail on how outcomes were measured or analyzed
Those based solely on opinions or informal observations</td></tr>
<tr><td>Context</td><td>Research conducted in healthcare or community settings of relevance such as
Tuberculosis clinics
Hospital settings
Primary care clinics
Community health programs
Any other relevant setting where tuberculosis and depression services may be provided
Global geographic inclusion</td><td>Research from unrelated contexts such as veterinary or laboratory studies.

No exclusion criteria based on geography</td></tr>
<tr><td>Language</td><td>English</td><td>Language other than English</td></tr>
<tr><td>Study Design</td><td>Primary studies
Randomized controlled trials
Prevalence surveys
Case studies
Cohort studies
Case-control studies
Systematic reviews</td><td>Meta-reviews (reviews of reviews)
Opinion pieces, editorials, and letters to the editor that do not contain empirical data
Abstracts without full-text availability
Protocol papers</td></tr>
</table>

	• Meta-analyses<br>• Scoping reviews<br>• Qualitative studies<br>• Mixed methods analyses<br>• Diagnostic test accuracy studies	

~~The preliminary search will focus on combinations of the following areas: terms related to "tuberculosis" and terms related to "depression". Appropriate MeSH terms will be utilized for each of the proposed databases (S1 Appendix).~~

Study ~~Selection~~selection

Formatted: Font: Bold, Font color: Text 1

Formatted: Heading 2, Line spacing: single

Formatted: Font: Bold, Font color: Text 1

We will use Covidence to screen study titles, abstracts and full text articles for inclusion. Two reviewers (AJG, PT) will review abstracts in parallel for inclusion. To minimize bias, reviewers will be blinded to other reviewers' ratings for screening except when resolving conflicts. Reviewers will meet after every 20 abstracts to resolve any conflicts and to propose/implement updates to the search strategy or inclusion criteria. All abstracts that pass screening will have the full text reviewed by the two reviewers, again, in parallel for final inclusion in the review. Disagreements will be resolved via discussion and, if needed, by a third reviewer (DWD).

Charting the ~~Data~~ data

Formatted: Font: Bold, Font color: Text 1

Formatted: Heading 2, Line spacing: single

Formatted: Font: Bold, Font color: Text 1

After piloting the data extraction tool, we will extract a~~A~~ll data will be extracted using Covidence. We will extract pertinent study details such as authors, location of publication/journal name, year of publication, research question addressed, study design, geographic context, date when study was conducted, study location (community, health facility, etc.), sample size, participants characteristics (such as age,

HIV status, sex, socioeconomic status, other mental health disorders, etc.), screening and diagnostic tools used for depression, treatment intervention approach utilized for depression (e.g. psychological approaches like cognitive-behavioral therapy, pharmacological like antidepressants, and/or alternative interventions like mindfulness techniques or yoga), treatment intervention components outcomes assessed, treatment intervention mode of delivery (e.g. in-person therapy, oral medication etc.), duration and frequency of treatment intervention, statistical methods used (e.g. regression analysis, chi-square, multi-level models etc.), comparison population (if a control or other comparator used), outcome measures (e.g, depression prevalence estimates, odds ratios etc.), measures of precision (e.g. 95% CI), statistical significance, and, for qualitative studies, key themes identified, barriers and facilitators to diagnosis and/or treatment identified, and the framework or content analysis utilized (i.e. thematic analysis, grounded theory, case study approach, etc.). Additionally, if the authors identify any limitations relevant to the research question, such as methodological limitations, we will extract this data. One author (AJG) will extract the data with a second reviewer (PT) checking extracted data for accuracy. These two authors will routinely meet during the data extraction phase to ensure the extraction tool is capturing all relevant data needed to answer the overall research question.

Results

Collating, ~~Summarizing~~summarizing, and ~~Reporting~~ reporting ~~Results~~results

We will provide descriptive characteristics of all studies included in the review using figures and tables to fully summarize the studies. While our focus in the present research is to characterize the current state of the literature with inclusion not dependent on literature quality, we will conduct a quality appraisal of each study as the presence or absence of high-quality research is relevant to understanding research gaps. As there will be many different study designs, to assess quality, we will use the Jadad scale for randomized controlled trials, the Newcastle-Ottawa scale for observational studies, and the Joanna Briggs Institute Critical Appraisal Tool for Qualitative Research for qualitative studies.

We will structure the reporting of our results using the RE-AIM (Reach, Effectiveness, Adoption, Implementation and Maintenance) framework (42). As we are interested in the way these two diseases have been studied together, for *reach*, we will determine the extent to which studies have explored both directions of the TB-depression relationship. We will also determine the reach of researchers in terms of including representative samples of PWTB, including diverse populations and healthcare settings, and their inclusion of high-risk groups (such as those with HIV, drug-resistant TB etc.). For *effectiveness* we will categorize the study designs used to assess the diversity of methodological approaches as a wide range of designs – such as cross-sectional, longitudinal and randomized controlled trials – is essential for fully elucidating the co-occurrence of TB and depression. A lack of diverse study designs may indicate gaps in

Formatted: Font: 18 pt, Bold, Font color: Text 1

Formatted: Heading 1, Line spacing: single

Formatted: Font: Bold, Font color: Text 1

Formatted: Font: Bold, Font color: Text 1

Formatted: Font: Bold, Font color: Text 1

the field's ability to fully understand and address this relationship. Additionally, we will determine the types of diagnostic measures used for depression along with their effectiveness (if reported) as well as how efficacious or effective any treatment interventions were at altering either depression or TB related outcomes. For *adoption* we will look at the extent to which depression screening, diagnosis, and treatment methodologies are being adopted in different settings (such as by national programs, referral centers, local clinics, etc) and by whom (e.g. physicians, nurses, community health works etc.) and examine the way in which research findings on these two diseases have impacted clinical guidelines and public health policies if mentioned by study authors. For *implementation*, we will determine barriers and facilitators identified to the implementation of depression related care in real-world TB treatment settings. Finally, for *maintenance* we will determine whether any depression related methodologies for diagnosis or treatment were sustained or embedded within existing health systems. This will include, if reported upon, long-term patient outcomes, if methodologies persisted beyond the research phase, and if any studies evaluated how health systems integrated and sustained any proposed diagnostic or treatment interventions.

Consultation with Stakeholders

Formatted: Line spacing: Double

We will include a consultation phase to enhance the relevance and applicability of our findings. We will engage a diverse group of stakeholders, drawing on global collaborators with expertise in TB and mental health from an established network of the research team, as well as individuals with lived experience of TB, including patients from Uganda who are participating in an unrelated, ongoing cohort study. These

consultations will help provide contextual insights into the interpretation of findings and identify additional knowledge gaps or implementation considerations that may not be fully captured in the published literature. Input from public health practitioners and researchers working in high TB burden settings will also be sought to ensure the review's implications are both meaningful and actionable.

Data Sharing

Formatted: Font color: Text 1

Formatted: Heading 2

In line with open science principles, we will make the final data extraction sheet and study metadata openly available through a public repository such as the Open Science Framework upon completion of the review.

Timeline

Formatted: Font: Bold, Font color: Text 1

Formatted: Heading 2, Line spacing: single

The initial search was conducted on March 31, 2025. All screening, including title and abstract screening and full text screening will take place from April to June 2025. Data extraction will take place in July 2025. Analysis and synthesis of results will take place from July to August 2025 with final results expected by September 2025.

Discussion

Formatted: Font: 18 pt, Bold, Font color: Text 1

Formatted: Heading 1, Line spacing: single

This scoping review will explore the existing literature on the intersection between depression and TB allowing us to identify research gaps, including in the diagnosis and treatment of depression, amongst PWTB. Importantly, this will allow us to understand any methodological gaps that exist and approaches that researchers have historically taken to tackle these two diseases concurrently.

By mapping the existing methodologies used to study depression in PWTB, this review will identify gaps in research design, highlight areas where more rigorous and diverse study approaches are needed, and provide insights into potential pathways for improving integrated care. Findings from this review will contribute to advancing research beyond cross-sectional prevalence surveys by encouraging the use of longitudinal, interventional, and mixed-methods studies that can better inform evidence-based practice. Furthermore, this review will underscore the urgent need for validated diagnostic tools and specialized treatment approaches to address the overlapping symptoms and complex interplay between tuberculosis and depression, ultimately supporting the development of more effective strategies to guide future research and public health interventions. Additionally, our review will be methodologically rigorous by using the Levac et al framework to ensure a systematic and transparent approach to study identification and selection. This will be enhanced through the use of RE-AIM to synthesize results, providing a structured lens to assess how methodological approaches to researching TB and depression have created knowledge gaps in studying the diseases concurrently. Finally, the planned stakeholder consultations will add depth and practical relevance to the final interpretation of the findings ensuring that the review will be both methodologically rigorous and highly relevant to global TB and mental health research practices.

Our review ~~does~~ will have some limitations that are worth discussing a priori. First, we have limited the search to English language studies. Given the burden of tuberculosis in many non-English speaking countries such as China where many publications are in

Mandarin, this may exclude several studies that would otherwise be eligible for this scoping review. Second, we are beginning this search with the term depression, a Western term, and its associated synonyms. Many countries use alternate terms for depression, such as shenjing shuairuo (nervous weakness) in China or yuutsu in Japan, meaning that our initial search may not fully capture the available literature. Given the long list of terms that could possibly be used by different countries, it is not likely we will be able to capture all possible terms and subsequently may miss eligible publications. Third, since the primary aim is to describe and categorize existing research rather than synthesize findings or estimate effect sizes, scoping reviews cannot make definitive conclusions about intervention effectiveness or causality. However, depending on the results of the current review, it may be appropriate to conduct a subsequent systematic review and meta-analysis to assess the public health impact of depression-related interventions amongst PWTB. Finally, as with all scoping reviews, the breadth of study types and topics can make data synthesis more complex than in a systematic review. Given our aim of establishing methodologies used in the field, a broad inclusion is necessary but could provide more narrative results. We believe our use of RE-AIM to structure the results, regardless of study type, will aid in creating meaningful outputs.

Conclusions

This scoping review will ~~further our knowledge of the extent of the literature concerning depression amongst those with TB by identifying research gaps for future researchers to fill~~advance understanding of how depression has been studied in the context of

Formatted: Font: Bold, Font color: Text 1

Formatted: Heading 2, Line spacing: single

tuberculosis by systematically mapping existing methodologies and identifying critical research gaps to guide future public health research and intervention development.

Acknowledgements

We would like to thank both Yale University and Johns Hopkins University for providing institutional support for this work.

Formatted: Font: 18 pt, Bold, Font color: Text 1

Formatted: Heading 1, Line spacing: single

Author Contributions

AJG conceptualized the research question and wrote the initial draft of this protocol. PT, JEG, and DWD provided substantial contributions to the conception and design of the protocol including reviewing it for intellectual merit and refinement of the research question. LR created the initial search queries and ran the initial search. All authors reviewed and approved the final manuscript.

Formatted: Font: 18 pt, Bold, Font color: Text 1

Formatted: Heading 1, Line spacing: single

References

Formatted: Font: 18 pt, Bold, Font color: Text 1

Formatted: Heading 1, Line spacing: Double

1. World Health Organization. Global Tuberculosis Report 2024. Geneva; 2024 29 October 2024.https://www.who.int/teams/global-programme-on-tuberculosis-and-lung-health/tb-reports/global-tuberculosis-report-2024

2. World Health Organization. Global Tuberculosis Report 2022. Geneva, Switzerland: World Health Organization; 2022.

3. Chaisson RE, Frick M, Nahid P. The scientific response to TB - the other deadly global health emergency. Int J Tuberc Lung Dis. 2022;26(3):186-9.

4. Venkatesan P. Worrying lack of funding for tuberculosis. Lancet Infect Dis. 2022;22(3):318.

5. Treatment Action Group. Tuberculosis Research Funding Trends 2005-2023. New York, New York; 2024 13 December, 2024.https://www.treatmentactiongroup.org/resources/tbrd-report/tbrd-report-2024/

6. Armstrong-Hough M, Turimumahoro P, Meyer AJ, Ochom E, Babirye D, Ayakaka I, et al. Drop-out from the tuberculosis contact investigation cascade in a routine public health setting in urban Uganda: A prospective, multi-center study. PLoS One. 2017;12(11):e0187145.

7. Kim J, Keshavjee S, Atun R. Health systems performance in managing tuberculosis: analysis of tuberculosis care cascades among high-burden and non-high-burden countries. J Glob Health. 2019;9(1):010423.

8. Subbaraman R, Jhaveri T, Nathavitharana RR. Closing gaps in the tuberculosis care cascade: an action-oriented research agenda. Journal of Clinical Tuberculosis and

Other Mycobacterial Diseases. 2020;19:100144. https://www.sciencedirect.com/science/article/pii/S2405579419300683

9. Jarde A, Romano E, Afaq S, Elsony A, Lin Y, Huque R, et al. Prevalence and risks of tuberculosis multimorbidity in low-income and middle-income countries: a meta-review. BMJ Open. 2022;12(9):e060906. http://bmjopen.bmj.com/content/12/9/e060906.abstract

10. Duarte R, Lönnroth K, Carvalho C, Lima F, Carvalho ACC, Muñoz-Torrico M, et al. Tuberculosis, social determinants and co-morbidities (including HIV). Pulmonology. 2018;24(2):115-9.

11. Bell LCK, Noursadeghi M. Pathogenesis of HIV-1 and Mycobacterium tuberculosis co-infection. Nat Rev Microbiol. 2018;16(2):80-90.

12. Méndez-Samperio P. Diagnosis of Tuberculosis in HIV Co-infected Individuals: Current Status, Challenges and Opportunities for the Future. Scand J Immunol. 2017;86(2):76-82.

13. Dorman SE. Diagnosis of HIV-associated tuberculosis. Curr Opin HIV AIDS. 2018;13(6):462-8.

14. Galea JT, Monedero-Recuero I, Sweetland AC. Beyond screening: a call for the routine integration of mental health care with tuberculosis treatment. Public Health Action. 2019;9(1):2.

15. Cáceres G, Calderon R, Ugarte-Gil C. Tuberculosis and comorbidities: treatment challenges in patients with comorbid diabetes mellitus and depression. Therapeutic Advances in Infectious Disease. 2022;9:20499361221095831. https://doi.org/10.1177/20499361221095831

16. Nightingale R, Carlin F, Meghji J, McMullen K, Evans D, van der Zalm MM, et al. Post-TB health and wellbeing. Int J Tuberc Lung Dis. 2023;27(4):248-83.

17. Chandra M, Rana P, Chandra K, Arora VK. Tuberculosis - Depression syndemic: A public health challenge. Indian J Tuberc. 2019;66(1):197-202.

18. Simon GE, Moise N, Mohr DC. Management of Depression in Adults: A Review. JAMA. 2024;332(2):141-52. https://doi.org/10.1001/jama.2024.5756

19. Haroz EE, Ritchey M, Bass JK, Kohrt BA, Augustinavicius J, Michalopoulos L, et al. How is depression experienced around the world? A systematic review of qualitative literature. Social Science & Medicine. 2017;183:151-62. https://www.sciencedirect.com/science/article/pii/S0277953616307109

20. Duko B, Bedaso A, Ayano G. The prevalence of depression among patients with tuberculosis: a systematic review and meta-analysis. Annals of General Psychiatry. 2020;19(1):30. https://doi.org/10.1186/s12991-020-00281-8

21. Cheng KC, Liao KF, Lin CL, Lai SW. Increased Risk of Pulmonary Tuberculosis in Patients with Depression: A Cohort Study in Taiwan. Front Psychiatry. 2017;8:235.

22. Hayward SE, Deal A, Rustage K, Nellums LB, Sweetland AC, Boccia D, et al. The relationship between mental health and risk of active tuberculosis: a systematic review. BMJ Open. 2022;12(1):e048945.

23. Verma AK, Singh A. Psychological disorders in tuberculosis: A narrative review. Indian Journal of Tuberculosis. 2024;71:S162-S4. https://www.sciencedirect.com/science/article/pii/S0019570724001641

24. Sweetland AC, Kritski A, Oquendo MA, Sublette ME, Norcini Pala A, Silva LRB, et al. Addressing the tuberculosis-depression syndemic to end the tuberculosis epidemic. Int J Tuberc Lung Dis. 2017;21(8):852-61.

25. Van Der Walt M, Keddy KH. The Tuberculosis-Depression Syndemic and Evolution of Pharmaceutical Therapeutics: From Ancient Times to the Future. Front Psychiatry. 2021;12:617751.

26. Wali A, Safdar N, Ambreen A, Hassan S, Yaqoob A, Mustafa T. Effects of extrapulmonary TB on patient quality of life and recurrence. Public Health Action. 2024;14(3):112-8.

27. Anye LC, Agbortabot Bissong ME, Njundah AL, Siewe Fodjo JN. Depression, anxiety and medication adherence among tuberculosis patients attending treatment centres in Fako Division, Cameroon: cross-sectional study. BJPsych Open. 2023;9(3):e65. https://www.cambridge.org/core/product/E5396B35E949366009834726AB74B8F0

28. Tomita A, Ramlall S, Naidu T, Mthembu SS, Padayatchi N, Burns JK. Major depression and household food insecurity among individuals with multidrug-resistant tuberculosis (MDR-TB) in South Africa. Social Psychiatry and Psychiatric Epidemiology. 2019;54(3):387-93. https://doi.org/10.1007/s00127-019-01669-y

29. Murugan Y, Patel N, Kumar V, Gandhi R. Mental Health Impacts of Multidrug-Resistant Tuberculosis in Patients and Household Contacts: A Mixed Methods Study. Cureus. 2024;16(5):e60412.

30. Dos Santos AP, Lazzari TK, Silva DR. Health-Related Quality of Life, Depression and Anxiety in Hospitalized Patients with Tuberculosis. Tuberc Respir Dis (Seoul). 2017;80(1):69-76.

31. Rathod S, Pinninti N, Irfan M, Gorczynski P, Rathod P, Gega L, et al. Mental Health Service Provision in Low- and Middle-Income Countries. Health Services Insights. 2017;10:1178632917694350. https://journals.sagepub.com/doi/abs/10.1177/1178632917694350

32. Molebatsi K, Wang Q, Dima M, Ho-Foster A, Modongo C, Zetola N, et al. Depression and delayed tuberculosis treatment initiation among newly diagnosed patients in Botswana. Global Public Health. 2021;16(7):1088-98. https://doi.org/10.1080/17441692.2020.1826049

33. Theron G, Peter J, Zijenah L, Chanda D, Mangu C, Clowes P, et al. Psychological distress and its relationship with non-adherence to TB treatment: a multicentre study. BMC Infect Dis. 2015;15:253.

34. Yan S, Zhang S, Tong Y, Yin X, Lu Z, Gong Y. Nonadherence to Antituberculosis Medications: The Impact of Stigma and Depressive Symptoms. Am J Trop Med Hyg. 2018;98(1):262-5.

35. Ruiz-Grosso P, Cachay R, de la Flor A, Schwalb A, Ugarte-Gil C. Association between tuberculosis and depression on negative outcomes of tuberculosis treatment: A systematic review and meta-analysis. PLOS ONE. 2020;15(1):e0227472. https://doi.org/10.1371/journal.pone.0227472

36. Ambaw F, Mayston R, Hanlon C, Medhin G, Alem A. Untreated depression and tuberculosis treatment outcomes, quality of life and disability, Ethiopia. Bull World Health Organ. 2018;96(4):243-55.

37. Fried EI, Robinaugh DJ. Systems all the way down: embracing complexity in mental health research. BMC Medicine. 2020;18(1):205. https://doi.org/10.1186/s12916-020-01668-w

38. Scheffer M, Bockting CL, Borsboom D, Cools R, Delecroix C, Hartmann JA, et al. A Dynamical Systems View of Psychiatric Disorders-Theory: A Review. JAMA Psychiatry. 2024;81(6):618-23.

39. Westhoff M, Berg M, Reif A, Rief W, Hofmann SG. Major Problems in Clinical Psychological Science and How to Address them. Introducing a Multimodal Dynamical Network Approach. Cognitive Therapy and Research. 2024;48(5):791-807. https://doi.org/10.1007/s10608-024-10487-9

40. Arksey H, O'Malley L. Scoping studies: towards a methodological framework. International Journal of Social Research Methodology. 2005;8(1):19-32. https://doi.org/10.1080/1364557032000119616

41. Levac D, Colquhoun H, O'Brien KK. Scoping studies: advancing the methodology. Implement Sci. 2010;5:69.

42. Glasgow RE, Vogt TM, Boles SM. Evaluating the public health impact of health promotion interventions: the RE-AIM framework. Am J Public Health. 1999;89(9):1322-7.

S1 Appendix: Initial Search terms by database

Dear Reviewers

We thank the review team for their careful consideration of our protocol manuscript. We believe the reviewers suggestions will improve both this protocol manuscript as well as our scoping review. Below we have responded to the reviewers suggestions as well as made changes where appropriate to the manuscript itself.

Best
Amanda J Gupta

Journal Requirements:

1. Please ensure that your manuscript meets PLOS ONE's style requirements, including those for file naming.

Please note, we have updated the formatting to meet PLOS ONE's style requirements. Thank you for directing us to the appropriate guides for doing so.

2. When completing the data availability statement of the submission form, you indicated that you will make your data available on acceptance. We strongly recommend all authors decide on a data sharing plan before acceptance, as the process can be lengthy and hold up publication timelines. Please note that, though access restrictions are acceptable now, your entire data will need to be made freely accessible if your manuscript is accepted for publication. This policy applies to all data except where public deposition would breach compliance with the protocol approved by your research ethics board. If you are unable to adhere to our open data policy, please kindly revise your statement to explain your reasoning and we will seek the editor's input on an exemption. Please be assured that, once you have provided your new statement, the assessment of your exemption will not hold up the peer review process.

We have updated the data availability statement to indicate that in the present submission, there is no data generated as this is a protocol manuscript.

Additional Editor Comments:

Consider complying with PLOS One guidelines for formatting manuscripts. Accordingly, the authors are urged to not subdivide the abstract into sub-sections. Furthermore, the authors are not expected to present results in any section of the scoping review protocol article given that the study results are not expected at this stage of the research process.

Thank you. We have updated the abstract to reflect this guidance. While this study itself has no results, we still believe there is a benefit in having a results section for the main body of the manuscript as it outlines how we will report the results of our scoping review.

I appreciate the figures provided regarding the epidemiologic and economic burden of TB. However, the authors could strengthen this paragraph by starting the introduction with the definition and classification (by the type of organ involvement: pulmonary versus extrapulmonary [specification of the extrapulmonary body organs that can be afflicted by TB required] TB;by disease activity: active TB versus latent TB; and by drug resistance: MDR-susceptible TB and MDR-resistant TB of tuberculosis) of TB, especially since PLOS One is a generalist journal. This would set the stage for the authors to make comments about TB throughout the manuscript (not just in the introduction) based on its subtypes, because there are indeed differences between pulmonary TB and extrapulmonary TB in the one hand, and between MDR-susceptible TB and MDR-resistant TB on the other hand. As a result, there may also bedifferences in the burden of depression across TB subtypes

Thank you for making this point. To ensure all your readers, including those not as well versed in TB, understand the burden that TB poses as well as the disease itself, we have updated the intro paragraph (lines 65-82) to the following to highlight the different types of TB along with their burden:

> Tuberculosis (TB), a communicable disease caused by *Mycobacterium tuberculosis,* is one of the leading infectious killers worldwide. While it primarily affects the lungs (pulmonary TB), it can be found in other places throughout the body (extrapulmonary TB) such as in the lymph nodes, pleura, bone and joints, urogenital tract, and meninges to name a few. The COVID-19 pandemic accelerated deaths due to TB as less attention, and subsequently funding, has been paid to TB (1, 2). This resulted in an estimated 10.8 million people developing new cases of active TB in 2023 with an estimated 1.25 million deaths due to TB disease (2). Active TB refers to disease in which TB bacteria are replicating and causing clinical symptoms. Pulmonary forms of active TB are typically symptomatic and infectious while extrapulmonary TB is usually symptomatic but not transmissible. This contrasts with latent TB which is a state in which an individual is infected with TB but is generally asymptomatic and non-infectious. It is estimated that billions of individuals worldwide have latent TB. Furthermore, TB can be classified by drug resistance with ~400,000 PWTB having multidrug-resistant TB (resistance to at least isoniazid and rifampin) in 2023 (2). Despite this large burden, TB is vastly underfunded leading to under-supported research endeavors and national TB programs left without appropriate funding to successfully combat the large TB burdens that they may face (1-5). This results in gaps in TB diagnosis, treatment, and care in high TB burden settings (6-8).

In addition, we added in the following sentence (line 111-126) to reflect the fact that there may exist differences in depression burden by TB subtype:

> Additionally, the burden of depression may also vary by TB subtype — individuals with extrapulmonary TB may face diagnostic delays and uncertainty that heighten psychological distress (26,27), while those with multidrug-resistant TB (MDR-TB) often endure prolonged treatment, more severe side effects, and greater social isolation, all of which may increase the risk or severity of depression (28,29).

Consider clarifying this statement. The revised statement could read: "While the diagnosis and treatment protocols of infectious TB comorbidities such as Human Immunodeficiency Virus (HIV) infection have been extensively researched, the diagnosis and treatment of comorbid non-communicable diseases (NCDs) such as mental health disorders have received little attention, even as the identification and treatment of comorbid NCDs has been shown to positively impact the TB outcome in affected patients."

We have updated the sentence to incorporate your suggested wording (see lines 85-92).

This sentence needs to be supported by at least one reference. Furthermore, it would be great to provide a multiperspective definition of depression after this sentence.

Suggested references:
1. PMID: 38357431 PMCID: PMC10863678 DOI: 10.32872/cpe.11699
2. https://doi.org/10.1016/j.socscimed.2016.12.030
3. PMID: 38856993 DOI: 10.1001/jama.2024.5756

Thank you for not only the suggestion but providing wonderful citations to go along with it! We have added in two TB specific citations for the original sentence in question (line 95) and have now added the following sentences (lines 95-100):

> Depression is a common mental health condition characterized by persistent sadness, loss of interest or pleasure, and functional impairment which can affect the overall quality of life of individuals suffering from it if not properly managed (18). While clinically defined in various tools, such as the DSM-5, its expression and recognition vary across cultural and social contexts increasing the diagnostic and treatment complexity of the condition (19).

Consider revising this paragraph to address the mechanisms (including the high frequency of HIV infection in the population with TB) underpinning the TB-depression syndemic, challenges in and facilitators for diagnosing and treating depression across TB subtypes, and the public health impact of diagnostic and therapeutic interventions targeting depression in individuals with TB.

In addition to the references provided by reviewer 1, these references could be helpful for this purpose:
1. PMID: 34140898 PMCID: PMC8203803 DOI: 10.3389/fpsyt.2021.617751
2. Wang J, Wu X,
Lai W, et al. Prevalence of
depression and depressive
symptoms among outpatients:
a systematic review and
meta-analysis. BMJ Open
2017;7:e017173. doi:10.1136/
bmjopen-2017-017173 for depression diagnosis in TN patients
3. https://doi.org/10.1016/j.genhosppsych.2021.01.006
4. https://doi.org/10.1038/s41392-024-01738-y

We have re-worked the introduction extensively (Lines 104-126) in an attempt to better capture the complexity of the underlying mechanisms of the TB-depression syndemic, and have now included the example of co-infection with HIV as an example of a potential underlying mechanism. We greatly appreciate your suggestion of references as well as those of Reviewer #1 and hope that our re-working of the introduction has now addressed your concerns.

I am unable to grasp the message conveyed by this statement.

We apologize for any lack of clarity. We have re-worked the sentence in question to the following (lines 130-131):

> From a public health perspective, untreated depression contributes to delays in TB diagnosis and treatment initiation (26, 28), non-adherence to TB medications (29, 30), and ultimately increases negative TB outcomes such as loss to follow up and death (31, 32).

This last part of the introduction should be revised to clearly state the rationale for conducting a scoping review on this topic and the aim of this protocol article. Based on the cover letter and the fact that the authors will use the RE-AIM framework for data synthesis, the rationale for this scoping review seems to be the need to guide future studies by providing a roadmap for integrating appropriate (from a public health perspective) diagnostic and therapeutic interventions for depression into routine care of different forms of TB: see Munn et al. BMC Medical Research Methodology (2018) 18:143 https://doi.org/10.1186/s12874-018-0611-x.
Alonmg with my comment about the rationale for the scoping review, a simpler delineation of the aim of this scoping review protocol article could be: to evaluate the public health impact of diagnostic and therapeutic interventions for depression in individuals with different forms of TB: see PMID: 10474547 PMCID: PMC1508772 DOI: 10.2105/ajph.89.9.1322.

Thank you for this suggestion. We have updated the final paragraph of the introduction as follows (lines 165-175):

> Given the substantial burden of depression among PWTB and the large focus on prevalence estimates within the literature with limited exploration of the methodological diversity and public health relevance of diagnostic and therapeutic approaches, a scoping review is particularly needed to broadly map the existing evidence, identify the most pressing knowledge gaps, and clarify how research has been conducted when studying these comorbid conditions. A scoping review would also help guide future studies by offering a roadmap for integrating effective and contextually appropriate mental health interventions into TB care. Therefore, we aim to conduct a scoping review to examine the methodologies used to study depression, including diagnostic and treatment methodologies, in PWTB, identifying research gaps and highlighting opportunities for improved methodological approaches in future public health research.

Because this is the protocol (not the scoping review) article, I suggest revising this statement in these likes:

"This section is presented in accordance with the scoping review framework by Arksey and O'Malley [26] and refined by Levac et al [27] which will guide the methodology of our scoping review."

Did you also register the protocol in OSF for example? Pieper and Rombey Systematic Reviews (2022) 11:8
https://doi.org/10.1186/s13643-021-01877-1

Thank you. We have revised the sentence per your suggestion. We have not registered the protocol in OSF yet but as is now mentioned in manuscript (lines 326-329) we will be making all relevant data from the review available in OSF upon completion of the review and will have the protocol registered prior to data extraction

Along with the potential rationale and aim of the scoping review mentioned above, is this question not more exact:

"What methods have been used so far to diagnose and treat depression across different forms of TB and did those methods have sizeable public health effects?
We thank you for your consideration of our research question. We believe that our current research question captures the aim of our review as we will not be able to answer the second part of your proposed question (“did those methods have sizeable public health effects”) since we will not be conducting a meta-analysis of any diagnostic tools or treatment interventions. We are seeking to

have a broad inclusion to see how researchers have tried to define the relationship between TB and depression. Studies may also be characterizing the depression risk profile of those with TB which would also be of interest beyond just treatment and diagnosis. That being said, depending on the results of the present scoping review, conducting a follow-up systematic review and meta-analysis looking at the public health impact of treatment interventions may be an appropriate next step.

Consider deleting this statement to leave only the research question in this sub-section.

We have deleted the sentence "We will screen studies that have sought to examine the relationship between TB and depression"

Can you reframe this statement to first enumerate the searched electronic databases and then state the reasons why you are planning to search those databases?

We have replaced the noted sentence with the following (lines 194-202):

> We will search the following electronic databases: MEDLINE, Embase, Global Health, the Cochrane Library, the WHO Regional Libraries, Africa-Wide Information, and PsycINFO. These databases were selected for their relevance to the scope of this review: MEDLINE, Embase, Global Health, and the Cochrane Library are widely used for biomedical and public health research; the WHO Regional Libraries and Africa-Wide Information are included to ensure geographic representation, particularly from high TB burden regions such as sub-Saharan Africa; and PsycINFO is included for its comprehensive coverage of psychological and mental health research.

Could you revise this statement to clearly specify that the electronic search will be supplemented by manual searches consisting of the scrutinization of reference lists of studies retrieved from electronic search? Will you also conduc grey literature searches? Notably, I anticipate that there are non-peer reviewed/non-published materials addressing this topic in a subset of African TB/mental health settings.

We have updated the referred to sentence to the following (lines 219-222):

> Finally, the electronic database search will be supplemented by manual searches, including the screening of reference lists from all included studies to identify any additional relevant articles that may have been missed.

We appreciate the suggestion to include grey literature and strongly considered it prior to the initial submission as well as again now. However, given the methodological focus of this scoping review and the resource constraints of our research team, we have chosen to limit our search to peer-reviewed literature to ensure consistency in reporting standards and methodological detail, which are often less clearly documented in grey literature sources, as we need studies fully detailing their methods in order to answer our research question.

Consider adding details about participants' age, sex and socioeconomic status (JBI Evid Synth. 2020 Oct;18(10):2119-2126. doi: 10.11124/JBIES-20-00167.)

Consider also mentioning a statement about comparators of participants with the TB-depression syndemic given that you will include interventional studies.

Within Table 1, we have added in wording to explicitly state that participants of any age, sex, or SES will be included.

I suggest reframing this statement like this:
"human participantss with only a clinical diagnosis of tuberculosis, those with a mycobaterial infection not due to M. tuberculosis (e.g., leprosy), and animal participants"

The fact that the authors also include participants with latent tuberculosis mean that they cannot exclude individuals considered to be affected by tuberculosis based solely on the presence of M. tuberculosis in biological samples.

We have updated Table 1 to make it more legible based on the recommendation of Reviewer 1. We have also integrated the language you mention here. To clarify the point on latent TB, we will include studies involving individuals with either active or latent tuberculosis, as long as there is confirmation of TB infection—regardless of the method used to establish that diagnosis (e.g., microbiological, radiological, or clinical assessment). Our aim is not to restrict inclusion based on a specific TB diagnostic modality but to ensure that participants included in studies have been clearly identified as having TB infection in accordance with the criteria used by the study authors.

It seems to me that the concept of the scoping review is "public health impact of diagnostic and therapeutic interventions for depression in all forms of TB". Accordingly, the authors would also like to specify here that they are planning to include any study design that could help assess the public health impact of diagnostic and therapeutic interventions for depression in TB patients. Along with a comment of Reviewer 2 and scoping review guidelines, this scoping review should include all

available evidence, regardless of of the publication language, especially since TB is highly prevalent in many countries where English is not among the official languages.

Thank you for this insightful comment. We would like to clarify that we will not be able to determine the public health impact of diagnostic and therapeutic interventions for depression in PWTB as we are not proposing to conduct a meta-analysis. Rather, we are focused on establishing the current state of the literature and highlight methodological gaps that exist in the study of these two co-morbid conditions.

Regarding language restrictions, while we recognize the importance of including non-English language publications—particularly given the global burden of TB—we must balance this with the capacity of our research team. Therefore, we regrettably must restrict the search to English language studies as we lack funding for translations and do not have at least 2 team members who speak additional languages beyond English. We will document and transparently report any exclusions based on language to acknowledge this as a potential limitation. This approach aims to maximize the inclusiveness of the review while remaining feasible within available resources.

I suggest splitting this arrow into two arrows: one on study setting and another one on the individuals who assessed the public health impact of diagnostic and therapeutic interventions for depression in TB patients. In the study setting arrow, the authors should report on i) the continental coverage of studies to be included, ii) countries where studies to be included should have been conducted, iii) study localities, i.e., urban, semi-urban, and rural; and housing platforms where included studies were conducted: community centres vs hospital centres, unspecialized versus specialized (TB centres versus mental health/depression centres versus TB-mental health centres [e.g., Jamot Hospital in Yaounde-Cameroon]) centres, and public health centres versus centres for purposes other than public health promotion. The second arrow would report on whether you will include studies regardless of credentials of individuals who evaluated the public health impact of diagnostic and therapeutic interventions for depression in individuals with TB: public health practioners versus others.

Thank you for this suggestion. We have re-worked Table 1 based on Reviewer #1's suggestions and believe that legibility and clarity has been improved. Many of the suggestions you make here are in our data extraction template for study details and we will be sure to report on them for our findings. Here we are wanting to make sure that the reader understands that the context is largely not determining the inclusion in this scoping review as long as the study has taken place within humans (i.e. not veterinary settings and not laboratory only settings)

This statement should come just after the statement on the searched databases in lines 109 to 113.

Could you try adding the forms of TB in preliminary search strategies and see whether this optimizes the sensitivity and specificity of search strategies?

PMID: 30271302 PMCID: PMC6148622 DOI: 10.5195/jmla.2018.283

McGowan et al. Journal of Clinical Epidemiology 75 (2016) 40e46

Duko et al. Ann Gen Psychiatry (2020) 19:30 https://doi.org/10.1186/s12991-020-00281-8

Consider mentioning the search strategies in tables in the main manuscript. The search strategy is a core part of a scoping review protocol article.

Thank you for suggesting this. Per your suggestion, we have now moved the referred to sentence to right after our discussion of databases (Lines 202-216).

Since we are using MESH terms and terminology suggested by the respective databases, we have captured all various forms of tuberculosis in the current search strategy for each database. Our team librarian (LR) ran many test searches prior to arriving on this particular strategy to ensure all types of TB would be included and all possible articles discussing depression and TB co-occurrence would be included as well.

While we agree that the search strategies are a core part of the scoping review protocol, we also recognize that they do not translate well to tables/figures. We would prefer to keep it as a supplemental document to ensure the appropriate formatting for each individual database is maintained for those readers who are interested in replicating or updating our results.

Would you mind replacing this phrase with "immunosuppressive status" to also consider other causes of immunosuppression?

Thank you for this thoughtful suggestion. While we understand the rationale for broadening the HIV phrasing to "immunosuppressive status," we have chosen to retain the specific reference to HIV in this context. Given the well-documented and disproportionately high prevalence of HIV co-infection among people with tuberculosis, as well as its significant implications for both TB progression and mental health outcomes, HIV remains the most relevant and commonly reported immunosuppressive condition in TB research. As such, the research team believes that specifying HIV is both appropriate and necessary to accurately reflect the epidemiological and clinical realities in this population. We will not however limit our data extraction to only HIV

related immunosuppressive states if a study does report on it. We just believe this is the most relevant to provide as an example.

Consider adding socioeconomic and nutritional status which are also important social determinants of TB.

Thank you. We have now added in.

Can you specify the theme analysis frameworks used in qualitative studies in brackets?

Medwave. 2024 Nov 22;24(10):e2972. doi: 10.5867/medwave.2024.10.2972.

We have now added in "(i.e. thematic analysis, grounded theory, case study approach, etc.)" *(lins 268-269).*

I see "narrative synthesis" nowhere in this paragraph despite this form of data synthesis being the main one used in the scoping review design.

Thank you for this comment. We recognize that narrative synthesis is commonly used in scoping reviews; however, for this study, we have intentionally chosen not to adopt a narrative synthesis approach. Instead, we will apply a structured framework-based synthesis using the RE-AIM framework to enhance methodological transparency and rigor. This approach allows us to systematically categorize and analyze the methodological characteristics of included studies in relation to public health impact, rather than relying on a primarily descriptive or thematic narrative. We believe this framework-guided strategy will provide greater clarity and utility for guiding future research and informing intervention development.

Can you specify the risk of bias assessment tools you plan to use?

Certainly! We have now added in the following sentence (Line 281-284):

> As there will be many different study designs, to assess quality, we will use the Jadad scale for randomized controlled trials, the Newcastle-Ottawa scale for observational studies, and the Joanna Briggs Institute Critical Appraisal Tool for Qualitative Research for qualitative studies

Will it not be more precise to say that you will assess the public health impact of diagnostic and therapeutic interventions for depression using the RE-AIM framework?

We will not be assessing the public health impact in this scoping review. We will be characterizing the state of existing literature. Assessing the public health impact could be done in a follow-up systematic review and meta-analysis depending on the findings of the current scoping review.

I suggest revising the text in lines 166-192 to clearly specify how the public health impact of depression diagnostic and therapeutic interventions will be assessed through the RE-AIM model ((Am J Public Health
. 1999 Sep;89(9):1322-7. doi: 10.2105/ajph.89.9.1322.). The revised text could read: "R for Reach, refers to the evaluation of how well depression diagnostic and therapeutic interventions were delivered to the target populations/high-risk TB groups.
E for efficacy, refers to the assessment of the accuracy (sensitivity, specificity, AUC , etc) of depression diagnostic methods, efficacy of depression treatment methods, as well as cost-benefit, cost-utility and cost-effectiveness (Iran J Public Health
. 2022 Oct;51(10):2159-2170. doi: 10.18502/ijph.v51i10.10975.) of depression diagnostic and treatment methods in individuals with different forms of tuberculosis. A for adoption, refers to proportions of the different individuals or legal/regulatory bodies that adopted depression diagnostic and treatment methods as well as barriers and facilitators to the adoption of depression diagnostic and intervention methods in TB patients. I for implementation, refers to... M for mainetnance, refers to...

Thank you for your thoughtful suggestion. We appreciate the recommendation to align our use of the RE-AIM framework with more traditional applications that focus on evaluating the public health impact of interventions. However, we would like to clarify that this scoping review is not designed to evaluate the effectiveness or real-world impact of depression diagnostic or therapeutic interventions among individuals with tuberculosis. Rather, the purpose of this review is to ***map and characterize the methodologies*** *used in the existing literature—including how depression is studied, diagnosed, and treated in TB populations—and to identify* ***knowledge gaps*** *that may inform future research and implementation efforts.*

*As such, we are applying the RE-AIM framework not to assess intervention outcomes per se, but to organize and interpret how existing studies reflect various dimensions relevant to public health research, such as who is being studied (**Reach**), what methodological designs are being used (**Effectiveness**, as a proxy), where and by whom depression-related methods are applied (**Adoption**), what contextual barriers and facilitators are reported (**Implementation**), and whether there is any evidence of longer-term integration (**Maintenance**). This use of RE-AIM aligns with its broader utility as a planning and evaluative framework that can guide the interpretation of complex evidence across multiple settings, even in the absence of formal impact evaluation (Glasgow et al., 1999).*

Consider addressing the "Cosnultation with stakeholders" sub-section of the O'Malley and Arksey framework just before this sub-section.

This sub-title should be in bold because it points to a sub-section.

Consider adding four sub-sections after this one and before the discussion section:

-Patient and public involvement (if you include patients/patient partners, how will you select them?)
-Dissemination and ethics
-Data sharing (in line with Reviewer 1's comment)
-Protocol deviation (is it possible that the protocol be modified even after the publication of this article? how will you deal with that issue in case it happens?)?

Rather than include both a section on patient/public involvement and a section on consultation with stakeholders, we have added in a section on Consultation with Stakeholders that explicitly states that we will draw on a diverse group of stakeholders, including PWTB currently participating in an ongoing and unrelated cohort study in Uganda to ensure that all conclusions and interpretations that are made as part of the scoping review are in line with lived experiences of relevant stakeholders. Given our teams extensive network of TB professionals across many continents, we believe we will be able to make meaningful conclusions about our work by drawing on the various experts within these networks. Please see the newly created section in Lines 316-326.

As mentioned below in our response to reviewer 1, we have added in Data Sharing (lines 327-330). We believe this covers the dissemination portion you mention above. We do not anticipate any protocol deviations as the protocol is considered “locked” and all team members have been involved in the process of creating this protocol.

Can you revise this paragraph specifying the strengths of the methods planned to be used in the future scoping review?

Certainly. While we believe the paragraph in question highlights many of the strengths for our review’s aims, we have now added in some additional strengths on the methodological rigor we plan to employ (lines 353-361):

> Additionally, our review will be methodologically rigorous by using the Levac et al framework to ensure a systematic and transparent approach to study identification and selection. This will be enhanced through the use of RE-AIM to synthesize results, providing a structured lens to assess how methodological approaches to researching TB and depression have created knowledge gaps in studying the diseases concurrently. Finally, the planned stakeholder consultations will add depth and practical relevance to the final interpretation of the findings ensuring that the review will be both methodologically rigorous and highly relevant to global TB and mental health research practices.

The review has not yet been conducted. Would you mind revising this statement delineating the anticipated limitations of the methods planned to be used in the

future scoping review? Focus should be made mainly on depression case-identification methods, limitations of the methods planned to be used for study identification, and anticipated limitations of RE-AIM assessment of the public health impact of depression diagnostic and therapeutic methods in individuals with different forms of TB.

As noted in the timeline section, we have conducted the initial search (on March 31, 2025). We are currently screening titles and abstracts now. Based on your comments and those of Reviewer 1, we have updated the limitations (lines 372-383). As noted above, determining the public health impact is beyond the scope of this review and is a question better suited for a subsequent systematic review and meta-analysis. Please see lines 372-3 for the newly added limitations about scoping review methodologies.

> Third, since the primary aim is to describe and categorize existing research rather than synthesize findings or estimate effect sizes, scoping reviews cannot make definitive conclusions about intervention effectiveness or causality. However, depending on the results of the current review, it may be appropriate to conduct a subsequent systematic review and meta-analysis to assess the public health impact of depression-related interventions amongst PWTB. Finally, as with all scoping reviews, the breadth of study types and topics can make data synthesis more complex than in a systematic review. Given our aim of establishing methodologies used in the field, a broad inclusion is necessary but could provide more narrative results. We believe our use of RE-AIM to structure the results, regardless of study type, will aid in creating meaningful outputs.

This should also be revised to strengthen it consistently with the aim of the planned scoping review.

We have re-worded the sentence in question as follows (lines 386-388):

> This scoping review will advance understanding of how depression has been studied in the context of tuberculosis by systematically mapping existing methodologies and identifying critical research gaps to guide future public health research and intervention development.

Reviewer #1: Review for: “Methodologies for Studying Depression in Persons living with Tuberculosis: Protocol for a Scoping Review“

The paper at hand is the study protocol for a scoping review about methodologies for studying depression in persons suffering from tuberculosis. The paper is generally well written. The authors refer to established guidelines of conducting scoping reviews. The inclusion of supporting information like the PRISMA checklist or the search terms is laudable. The high prevalence of

depression in tuberculosis and the detrimental consequences of both conditions makes this a very important scientific endeavor. In my view this work shows great promise and I applaud the authors for their work. Nevertheless, I have some concerns and comments that I feel need to be addressed. Please find my detailed review below.

My main point concerns the description of the aim of the planned scoping review. The methods section mostly seem to indicate that the aim is to find treatment trials, i.e., trials in which either depression and/or tuberculosis are treated. In the results section you convey that you will consider diverse study types. This seems good because treatment studies are not the only way to find out more about any associations between the two diseases. In any case, after the revision, the paper should give some examples for typical types of studies that you aim to include. I get that you cannot anticipate every type of study that explores the interrelationships between tuberculosis and depression but at the moment it is hard to grasp how do you aim to get relevant information on comorbidity and treatment options and whether interventional studies are the backbone of your review or one source of many. I don't think there is necessarily a problem with the content here, but rather difficulties arise due to the manner in which the research plan and the narrative flow of the text are presented.

Thank you for your thoughtful review and comments. We believe that we have updated the methods section to better reflect the fact that we will be going far beyond treatment studies for the present review but rather capturing any study that is aiming to characterize the relationship between TB and depression. We believe that our re-design of Table 1 (based on one of your later comments) in particular now makes it quite clear that we are aiming for a broad range of studies in order to characterize methodological gaps that exist in the study of comorbid depression and TB. We hope that our edits have made the narrative flow more clear to ensure readers understand the aim of our review.

The second major point that strikes me is the theoretical embedding of the connections between tuberculosis and depression. It is quite well deduced that it exists and that it should be investigated further. However, it does not go into what previous findings have shown as to why this connection exists. I am not an expert in this field. However, I wonder what psychological and/or biological factors could be interacting here? Is there relevant biopsychological literature regarding the interrelationship? What about the neuroscience of tuberculosis?

I also wonder whether meta-theories such as the “complex systems” perspective (which I am more familiar with) could be utilized to explain the relationships?

Fried, E. I., & Robinaugh, D. J. (2020). Systems all the way down: embracing complexity in mental health research. BMC medicine, 18, 1-4.

Scheffer, M., Bockting, C. L., Borsboom, D., Cools, R., Delecroix, C., Hartmann, J.

A., ... & Nelson, B. (2024). A dynamical systems view of psychiatric disorders—theory: a review. JAMA psychiatry, 81(6), 618-623.

Westhoff, M., Berg, M., Reif, A., Rief, W., & Hofmann, S. G. (2024). Major problems in clinical psychological science and how to address them. Introducing a multimodal dynamical network approach. Cognitive Therapy and Research, 48(5), 791-807.

Thank you for highlighting the complexity of TB and depression co-occurrence. It is clear that you carefully reviewed our manuscript with the intention of improving our thinking and approach and we are deeply appreciative of your careful consideration and advice.

We have updated our introduction extensively and have included examples of the biological and psychological interactions (detailed more below). We have also taken your suggestion about highlighting complex systems thinking and have used many of the references you suggested to highlight the utility in using systems thinking as an approach to TB and depression. Please see the newly added sentences about systems thinking (lines 142-163):

> Given this, a complex systems thinking approach (37-39) may be of use to researchers as it highlights the importance of moving beyond isolated risk factors to better consider the dynamic, bidirectional, and context-dependent interactions between TB and depression. This perspective also underscores the need for diverse and multi-level methodological approaches that can better capture feedback loops (e.g. how depression worsens TB outcomes, which may in turn exacerbate depressive symptoms), variability across TB subtypes, and health system contexts. Furthermore, applying such a lens can help identify critical gaps in the current research landscape and more effectively guide future investigative efforts.

More importantly, there seem to be reviews that discuss possible reasons for mental health problems in tuberculosis patients that you could cite (or discuss in more depth).
Cite:

Alene, K. A., Wangdi, K., Colquhoun, S., Chani, K., Islam, T., Rahevar, K., ... & Viney, K. (2021). Tuberculosis related disability: a systematic review and meta-analysis. BMC medicine, 19, 1-19.
Discuss in more depth:

Hayward, S. E., Deal, A., Rustage, K., Nellums, L. B., Sweetland, A. C., Boccia, D., ... & Friedland, J. S. (2022). The relationship between mental health and risk of active tuberculosis: a systematic review. BMJ open, 12(1), e048945.

Cáceres, G., Calderon, R., & Ugarte-Gil, C. (2022). Tuberculosis and comorbidities: treatment challenges in patients with comorbid diabetes mellitus and depression. Therapeutic Advances in Infectious Disease, 9, 20499361221095831.

I get that an extensive discussion might be to much for a protocol paper but some hints into the possible nature of the relationships should be given and be it on a meta (e.g., systems) level. For a protocol paper, I think that one to two paragraphs are enough but the reader will need some context information here.

We have now added additional details and examples into the introduction about the intersecting biological, social and structural factors that can increase the risk or severity of depression for those living with TB. We have also added in numerous references, including many that you suggest here. Please see the entire introduction which has been extensively re-worked or this excerpt here which focuses on the mechanisms underlying depression and tuberculosis (lines 104-126):

> Depression may not only be a consequence of TB, but also a potential risk factor due to its effects on immune function, health-seeking behavior, and treatment engagement (21, 22). Furthermore, TB-depression co-occurrence can be thought of as a syndemic, or an interacting set of conditions that cluster within social disadvantage, including intersecting biological, social, and structural factors, that results in an increased burden of disease (23-25). For example, HIV infection, a highly prevalent co-morbidity in PWTB, contributes to immunosuppression, social stigma, and economic vulnerability, all of which heighten susceptibility to mental health disorders (20). Additionally, the burden of depression may also vary by TB subtype —individuals with extrapulmonary TB may face diagnostic delays and uncertainty that heighten psychological distress (26, 27), while those with multidrug-resistant TB (MDR-TB) often endure prolonged treatment, more severe side effects, greater economic uncertainty, and greater social isolation, all of which may increase the risk or severity of depression (28, 29)

Finally, the protocol should have a statement about open science practices. Will the extraction sheet be made openly available? How do you plan to share important study metadata? If you aim to share your data, at which repository do you want to share it? If you do not aim to share your data, this deserves justification in my view.

We have now added the following sentence (lines 327-330):

In line with open science principles, we will make the final data extraction sheet and study metadata openly available through a public repository such as the Open Science Framework upon completion of the review.

Minor

Sometimes you seem to use [] for indicating your sources and sometimes you use (). Please stick to a standardized style of reference indicators.

Thank you. We have now ensured that our citations fully comply with PLOS ONE guidelines.

The list of references is quite short at the moment. I am aware that this is a protocol paper but I would think that the paper would benefit from additional sources.

We have now greatly added to the overall number of references based on your suggestions as well as those of the other reviewers. Thank you for this suggestion as well as specific suggestions of papers to cite which we believe have greatly enhanced the rigor of our manuscript.

Line 55: “is one of the leading infectious killers worldwide”: I would recommend against using “killer” and instead use words like lethality / mortality here. Furthermore, I would recommend you cite relevant literature to support this (true) claim.

We have altered the phrasing to now say “leading infectious causes of mortality” and cite the Global Tuberculosis Report 2024.

Line 74: Please remove the “staggering”.

We have removed “staggering” as you suggest.

Table 1 contains long sentences and is really difficult to read. I think it needs substantial rework. I would recommend that you shorten the text, consider using bullet points instead of sentences and consider using spacing options to make your points clearer.

We have reformatted Table 1 with bullet points as you suggest as well as removed some wording. We believe this has improved the legibility of the table.

Line 115: Good that you screen the references of the included papers. Consider including relevant literature for the benefits of footnote chasing.

Thank you for this suggestion. We agree that citation chasing can be a valuable method for identifying additional relevant studies. However, given the scope of our initial database search, which returned over 15,000 citations, and the resource constraints of our research team, we have opted to limit citation chasing to the references of included studies only, as originally planned. We believe this approach balances comprehensiveness with feasibility and aligns with the methodological standards for scoping reviews.

Limitations section: I would recommend that you critically discuss the strength and shortcomings of scoping reviews compared to traditional systematic reviews and meta-analyses and justify your choice for the scoping review. What I like about the limitations section is the critical discussion of cultural aspects.

We have added in additional limitations based on the general study design as follows (lines 372-383):

> Third, since the primary aim is to describe and categorize existing research rather than synthesize findings or estimate effect sizes, scoping reviews cannot make definitive conclusions about intervention effectiveness or causality. However, depending on the results of the current review, it may be appropriate to conduct a subsequent systematic review and meta-analysis to assess the public health impact of depression-related interventions amongst PWTB. Finally, as with all scoping reviews, the breadth of study types and topics can make data synthesis more complex than in a systematic review. Given our aim of establishing methodologies used in the field, a broad inclusion is necessary but could provide more narrative results. We believe our use of RE-AIM to structure the results, regardless of study type, will aid in creating meaningful outputs.

Line 219: "Our review does have": Maybe write: "Our review will have some limitations that are worth pointing out a priori"... Right now it sounds as if the review was already conducted. Down below in the conclusions section you use "will" and I would recommend also using it here.

We have adopted the language you propose here (Line 363).

I support an open and transparent review process. Therefore, I do not wish to stay anonymous.
Thanks to the authors and to the journal editors for giving me the opportunity to review this paper.

Max Berg,

University of Marburg
Clinical Psychology Group

Again, we would like to thank you for your thoughtful and constructive review. We believe it has enhanced our protocol manuscript and we are grateful that you participated in this peer review process.

Reviewer #2: The article is a study protocol for a scoping review aimed at mapping methodologies used to study depression in individuals with tuberculosis (TB). The primary objective is to identify gaps in research design, diagnostic tools, and treatment strategies for depression in TB patients. The authors highlight the co-occurrence of TB and depression and the need for robust research approaches to improve integrated care. The protocol outlines a systematic approach to reviewing existing literature, with the goal of guiding future research and public health interventions.

Methodology Evaluation:
The study employs the Arksey and O'Malley framework, updated by Levac et al., for conducting scoping reviews, which is appropriate for mapping broad research areas. The protocol includes six steps: identifying the research question, selecting relevant studies, charting data, collating results, and consulting stakeholders. The search strategy is comprehensive, covering databases like MEDLINE, Embase, PsycINFO, and Africa-Wide Information, ensuring a global perspective. Inclusion criteria are well-defined, focusing on studies that examine the TB-depression relationship, while exclusion criteria eliminate irrelevant or low-quality studies.

Thank you for this summary and highlighting some of the strengths of our approach!

Data extraction will be performed using Covidence, with two reviewers screening abstracts and full texts to minimize bias. The RE-AIM framework (Reach, Effectiveness, Adoption, Implementation, Maintenance) will structure the analysis, which is a strength for evaluating the impact of research methodologies. However, the protocol limits the search to English-language studies, potentially excluding relevant research from high-TB-burden, non-English-speaking countries. Additionally, the reliance on Western terms like "depression" may overlook culturally specific expressions of mental health conditions.

As mentioned above, regarding language restrictions, while we recognize the importance of including non-English language publications—particularly given the global burden of TB—we must balance this with the capacity of our research team. Therefore, we regrettably must restrict the search to English language studies as we lack funding for translations and do not have at least 2 team members who speak additional languages beyond English. We will document and transparently report any exclusions

based on language to acknowledge this as a potential limitation. This approach aims to maximize the inclusiveness of the review while remaining feasible within available resources.

You are right to highlight the reliance on Western terms and we were sure to specify it as a key limitation in our discussion section. After running test searches, we expanded our criteria to include broad keywords such as “Mental Health”, “Mood Disorder”, “Mental Disorder” and other similar words (please see S1 Appendix). We determined that these types of keywords resulted in studies from all continents and not just Western countries. We do however know that it is a limitation of our approach and why we have reported it here (lines 364-369), and will continue to report it in our subsequent findings.

Strengths and Weaknesses:
Strengths:

Comprehensive search strategy across multiple databases.

Clear inclusion/exclusion criteria to ensure relevance.

Use of the RE-AIM framework to evaluate methodological impact.

Dual-reviewer process to reduce bias in study selection.

Weaknesses:

Language restriction (English-only) may exclude significant studies.

Potential cultural bias in defining depression, missing non-Western perspectives.

Thank you again for highlighting these weaknesses. Please see above our response to these weaknesses.

No pilot data or preliminary results to validate the approach.

We did conduct piloting of the search strategy over several weeks and agree it was an oversight on our part to not make that clear in the text. We have now added in sentences (Lines 202 – 217) to make that process clear.

> The preliminary search strategy, constructed by a trained medical librarian (LR) after several weeks of testing various combinations of terms, focused on combinations of the following areas: terms related to “tuberculosis” and terms related to “depression”.

Limited discussion of how heterogeneity in study designs will be managed during synthesis.

We have now added in the following to highlight the different ways we will assess study quality by study design (Lines 281-284):

> As there will be many different study designs, to assess quality, we will use the Jadad scale for randomized controlled trials, the Newcastle-Ottawa scale for observational studies, and the Joanna Briggs Institute Critical Appraisal Tool for Qualitative Research for qualitative studies.

Additionally, we propose using RE-AIM, which can be used for any study design type, to assist in creating coherence across many contexts, study designs, and study populations. Finally, we have explicitly added into the limitations section a discussion on the difficulties that heterogeneous study designs can present (lines 377-383).

> Finally, as with all scoping reviews, the breadth of study types and topics can make data synthesis more complex than in a systematic review. Given our aim of establishing methodologies used in the field, a broad inclusion is necessary but could provide more narrative results. We believe our use of RE-AIM to structure the results, regardless of study type, will aid in creating meaningful outputs.

Conclusion and Recommendations:
This protocol provides a rigorous framework for a scoping review that could significantly advance understanding of depression in TB patients. However, the exclusion of non-English studies and culturally specific terminology may limit its comprehensiveness. Future research should:

Expand language inclusion to capture studies from high-TB-burden regions.

While we hope that we have captured most of the relevant literature, this unfortunately remains a limitation given our study team and resource availability.

Incorporate culturally adapted definitions of depression.

As mentioned above, we have now made it clear that the search critera were developed after testing numerous combinations to maximize the number of terms captured. However, we will continue to point out of difficult it can be to capture all possible terms used for depression, especially outside the Western context.

Pilot the methodology to refine search terms and data extraction processes.

We have now explicitly stated that the search was created after weeks of piloting the process. We have now also specified that the data extraction tool will be piloted prior to implementation (line 250).

Address heterogeneity in study designs during data synthesis.

Please see our response above.

Overall, the protocol is well-structured and promises valuable insights, but its impact could be enhanced by addressing these limitations.

Thank you again for your thoughtful comments. They have helped to improve our manuscript and we are deeply appreciative that you have participated in the peer-review process.
