# Supplementary material for: Methodologies for studying depression in persons living with tuberculosis: Protocol for a scoping review
Source: PLoS One. 2025 Jul 21;20(7):e0322753. doi: 10.1371/journal.pone.0322753 (PMC12279151; doi:10.1371/journal.pone.0322753)
Supplement: Attachment — Submitted filename: Response to Reviewers.docx [file pone.0322753.s004.docx]

Dear Reviewers

We thank the review team for their careful consideration of our protocol manuscript. We believe the reviewers suggestions will improve both this protocol manuscript as well as our scoping review. Below we have responded to the reviewers suggestions as well as made changes where appropriate to the manuscript itself.

Best

Amanda J Gupta

**Journal Requirements:**

**1. Please ensure that your manuscript meets PLOS ONE's style requirements, including those for file naming.**

*Please note, we have updated the formatting to meet PLOS ONE’s style requirements. Thank you for directing us to the appropriate guides for doing so.*

**2. When completing the data availability statement of the submission form, you indicated that you will make your data available on acceptance. We strongly recommend all authors decide on a data sharing plan before acceptance, as the process can be lengthy and hold up publication timelines. Please note that, though access restrictions are acceptable now, your entire data will need to be made freely accessible if your manuscript is accepted for publication. This policy applies to all data except where public deposition would breach compliance with the protocol approved by your research ethics board. If you are unable to adhere to our open data policy, please kindly revise your statement to explain your reasoning and we will seek the editor's input on an exemption. Please be assured that, once you have provided your new statement, the assessment of your exemption will not hold up the peer review process.**

*We have updated the data availability statement to indicate that in the present submission, there is no data generated as this is a protocol manuscript.*

**Additional Editor Comments:**

**Consider complying with PLOS One guidelines for formatting manuscripts. Accordingly, the authors are urged to not subdivide the abstract into sub-sections. Furthermore, the authors are not expected to present results in any section of the scoping review protocol article given that the study results are not expected at this stage of the research process.**

*Thank you. We have updated the abstract to reflect this guidance. While this study itself has no results, we still believe there is a benefit in having a results section for the main body of the manuscript as it outlines how we will report the results of our scoping review.*

**I appreciate the figures provided regarding the epidemiologic and economic burden of TB. However, the authors could strengthen this paragraph by starting the introduction with the definition and classification (by the type of organ involvement: pulmonary versus extrapulmonary [specification of the extrapulmonary body organs that can be afflicted by TB required] TB;by disease activity: active TB versus latent TB; and by drug resistance: MDR-susceptible TB and MDR-resistant TB of tuberculosis) of TB, especially since PLOS One is a generalist journal. This would set the stage for the authors to make comments about TB throughout the manuscript (not just in the introduction) based on its subtypes, because there are indeed differences between pulmonary TB and extrapulmonary TB in the one hand, and between MDR-susceptible TB and MDR-resistant TB on the other hand. As a result, there may also bedifferences in the burden of depression across TB subtypes**

*Thank you for making this point. To ensure all your readers, including those not as well versed in TB, understand the burden that TB poses as well as the disease itself, we have updated the intro paragraph (lines 65-82) to the following to highlight the different types of TB along with their burden:*

Tuberculosis (TB), a communicable disease caused by *Mycobacterium tuberculosis,* is one of the leading infectious killers worldwide. While it primarily affects the lungs (pulmonary TB), it can be found in other places throughout the body (extrapulmonary TB) such as in the lymph nodes, pleura, bone and joints, urogenital tract, and meninges to name a few. The COVID-19 pandemic accelerated deaths due to TB as less attention, and subsequently funding, has been paid to TB (1, 2). This resulted in an estimated 10.8 million people developing new cases of active TB in 2023 with an estimated 1.25 million deaths due to TB disease (2). Active TB refers to disease in which TB bacteria are replicating and causing clinical symptoms. Pulmonary forms of active TB are typically symptomatic and infectious while extrapulmonary TB is usually symptomatic but not transmissible. This contrasts with latent TB which is a state in which an individual is infected with TB but is generally asymptomatic and non-infectious. It is estimated that billions of individuals worldwide have latent TB. Furthermore, TB can be classified by drug resistance with ~400,000 PWTB having multidrug-resistant TB (resistance to at least isoniazid and rifampin) in 2023 (2). Despite this large burden, TB is vastly underfunded leading to under-supported research endeavors and national TB programs left without appropriate funding to successfully combat the large TB burdens that they may face (1-5). This results in gaps in TB diagnosis, treatment, and care in high TB burden settings (6-8).

*In addition, we added in the following sentence (line 111-126) to reflect the fact that there may exist differences in depression burden by TB subtype:*

Additionally, the burden of depression may also vary by TB subtype —individuals with extrapulmonary TB may face diagnostic delays and uncertainty that heighten psychological distress (26,27), while those with multidrug-resistant TB (MDR-TB) often endure prolonged treatment, more severe side effects, and greater social isolation, all of which may increase the risk or severity of depression (28,29).

**Consider clarifying this statement. The revised statement could read: "While the diagnosis and treatment protocols of infectious TB comorbidities such as Human Immunodeficiency Virus (HIV) infection have been extensively researched, the diagnosis and treatment of comorbid non-communicable diseases (NCDs) such as mental health disorders have received little attention, even as the identification and treatment of comorbid NCDs has been shown to positively impact the TB outcome in affected patients."**

*We have updated the sentence to incorporate your suggested wording (see lines 85-92).*

**This sentence needs to be supported by at least one reference. Furthermore, it would be great to provide a multiperspective definition of depression after this sentence.**

**Suggested references:**

**1. PMID: 38357431 PMCID: PMC10863678 DOI: 10.32872/cpe.11699**

**2. https://doi.org/10.1016/j.socscimed.2016.12.030**

**3. PMID: 38856993 DOI: 10.1001/jama.2024.5756**

*Thank you for not only the suggestion but providing wonderful citations to go along with it! We have added in two TB specific citations for the original sentence in question (line 95) and have now added the following sentences (lines 95-100):*

Depression is a common mental health condition characterized by persistent sadness, loss of interest or pleasure, and functional impairment which can affect the overall quality of life of individuals suffering from it if not properly managed (18). While clinically defined in various tools, such as the DSM-5, its expression and recognition vary across cultural and social contexts increasing the diagnostic and treatment complexity of the condition (19).

**Consider revising this paragraph to address the mechanisms (including the high frequency of HIV infection in the population with TB) underpinning the TB-depression syndemic, challenges in and facilitators for diagnosing and treating depression across TB subtypes, and the public health impact of diagnostic and therapeutic interventions targeting depression in individuals with TB.**

**In addition to the references provided by reviewer 1, these references could be helpful for this purpose:**

**1. PMID: 34140898 PMCID: PMC8203803 DOI: 10.3389/fpsyt.2021.617751**

**2. Wang J, Wu X,**

**Lai W, et al. Prevalence of**

**depression and depressive**

**symptoms among outpatients:**

**a systematic review and**

**meta-analysis. BMJ Open**

**2017;7:e017173. doi:10.1136/**

**bmjopen-2017-017173 for depression diagnosis in TN patients**

**3. https://doi.org/10.1016/j.genhosppsych.2021.01.006**

**4.** [**https://doi.org/10.1038/s41392-024-01738-y**](https://doi.org/10.1038/s41392-024-01738-y)

*We have re-worked the introduction extensively (Lines 104-126) in an attempt to better capture the complexity of the underlying mechanisms of the TB-depression syndemic, and have now included the example of co-infection with HIV as an example of a potential underlying mechanism. We greatly appreciate your suggestion of references as well as those of Reviewer #1 and hope that our re-working of the introduction has now addressed your concerns.*

**I am unable to grasp the message conveyed by this statement.**

*We apologize for any lack of clarity. We have re-worked the sentence in question to the following (lines 130-131):*

From a public health perspective, untreated depression contributes to delays in TB diagnosis and treatment initiation (26, 28), non-adherence to TB medications (29, 30), and ultimately increases negative TB outcomes such as loss to follow up and death (31, 32).

**This last part of the introduction should be revised to clearly state the rationale for conducting a scoping review on this topic and the aim of this protocol article. Based on the cover letter and the fact that the authors will use the RE-AIM framework for data synthesis, the rationale for this scoping review seems to be the need to guide future studies by providing a roadmap for integrating appropriate (from a public health perspective) diagnostic and therapeutic interventions for depression into routine care of different forms of TB: see Munn et al. BMC Medical Research Methodology (2018) 18:143**

**https://doi.org/10.1186/s12874-018-0611-x.**

**Alonmg with my comment about the rationale for the scoping review, a simpler delineation of the aim of this scoping review protocol article could be: to evaluate the public health impact of diagnostic and therapeutic interventions for depression in individuals with different forms of TB: see PMID: 10474547 PMCID: PMC1508772 DOI: 10.2105/ajph.89.9.1322.**

*Thank you for this suggestion. We have updated the final paragraph of the introduction as follows (lines 165-175):*

Given the substantial burden of depression among PWTB and the large focus on prevalence estimates within the literature with limited exploration of the methodological diversity and public health relevance of diagnostic and therapeutic approaches, a scoping review is particularly needed to broadly map the existing evidence, identify the most pressing knowledge gaps, and clarify how research has been conducted when studying these comorbid conditions. A scoping review would also help guide future studies by offering a roadmap for integrating effective and contextually appropriate mental health interventions into TB care. Therefore, we aim to conduct a scoping review to examine the methodologies used to study depression, including diagnostic and treatment methodologies, in PWTB, identifying research gaps and highlighting opportunities for improved methodological approaches in future public health research.

**Because this is the protocol (not the scoping review) article, I suggest revising this statement in these likes:**

**"This section is presented in accordance with the scoping review framework by Arksey and O'Malley [26] and refined by Levac et al [27] which will guide the methodology of our scoping review."**

**Did you also register the protocol in OSF for example? Pieper and Rombey Systematic Reviews (2022) 11:8**

[**https://doi.org/10.1186/s13643-021-01877-1**](https://doi.org/10.1186/s13643-021-01877-1)

*Thank you. We have revised the sentence per your suggestion. We have not registered the protocol in OSF yet but as is now mentioned in manuscript (lines 326-329) we will be making all relevant data from the review available in OSF upon completion of the review and will have the protocol registered prior to data extraction*

**Along with the potential rationale and aim of the scoping review mentioned above, is this question not more exact:**

**"What methods have been used so far to diagnose and treat depression across different forms of TB and did those methods have sizeable public health effects?**

*We thank you for your consideration of our research question. We believe that our current research question captures the aim of our review as we will not be able to answer the second part of your proposed question (“did those methods have sizeable public health effects”) since we will not be conducting a meta-analysis of any diagnostic tools or treatment interventions. We are seeking to have a broad inclusion to see how researchers have tried to define the relationship between TB and depression. Studies may also be characterizing the depression risk profile of those with TB which would also be of interest beyond just treatment and diagnosis. That being said, depending on the results of the present scoping review, conducting a follow-up systematic review and meta-analysis looking at the public health impact of treatment interventions may be an appropriate next step.*

**Consider deleting this statement to leave only the research question in this sub-section.**

*We have deleted the sentence “We will screen studies that have sought to examine the relationship between TB and depression”*

**Can you reframe this statement to first enumerate the searched electronic databases and then state the reasons why you are planning to search those databases?**

*We have replaced the noted sentence with the following (lines 194-202):*

We will search the following electronic databases: MEDLINE, Embase, Global Health, the Cochrane Library, the WHO Regional Libraries, Africa-Wide Information, and PsycINFO. These databases were selected for their relevance to the scope of this review: MEDLINE, Embase, Global Health, and the Cochrane Library are widely used for biomedical and public health research; the WHO Regional Libraries and Africa-Wide Information are included to ensure geographic representation, particularly from high TB burden regions such as sub-Saharan Africa; and PsycINFO is included for its comprehensive coverage of psychological and mental health research.

**Could you revise this statement to clearly specify that the electronic search will be supplemented by manual searches consisting of the scrutinization of reference lists of studies retrieved from electronic search? Will you also conduc grey literature searches? Notably, I anticipate that there are non-peer reviewed/non-published materials addressing this topic in a subset of African TB/mental health settings.**

*We have updated the referred to sentence to the following (lines 219-222):*

Finally, the electronic database search will be supplemented by manual searches, including the screening of reference lists from all included studies to identify any additional relevant articles that may have been missed.

*We appreciate the suggestion to include grey literature and strongly considered it prior to the initial submission as well as again now. However, given the methodological focus of this scoping review and the resource constraints of our research team, we have chosen to limit our search to peer-reviewed literature to ensure consistency in reporting standards and methodological detail, which are often less clearly documented in grey literature sources, as we need studies fully detailing their methods in order to answer our research question.*

**Consider adding details about participants' age, sex and socioeconomic status (JBI Evid Synth. 2020 Oct;18(10):2119-2126. doi: 10.11124/JBIES-20-00167.)**

**Consider also mentioning a statement about comparators of participants with the TB-depression syndemic given that you will include interventional studies.**

*Within Table 1, we have added in wording to explicitly state that participants of any age, sex, or SES will be included.*

**I suggest reframing this statement like this:**

**"human participantss with only a clinical diagnosis of tuberculosis, those with a mycobaterial infection not due to M. tuberculosis (e.g., leprosy), and animal participants"**

**The fact that the authors also include participants with latent tuberculosis mean that they cannot exclude individuals considered to be affected by tuberculosis based solely on the presence of M. tuberculosis in biological samples.**

*We have updated Table 1 to make it more legible based on the recommendation of Reviewer 1. We have also integrated the language you mention here. To clarify the point on latent TB, we will include studies involving individuals with either active or latent tuberculosis, as long as there is confirmation of TB infection—regardless of the method used to establish that diagnosis (e.g., microbiological, radiological, or clinical assessment). Our aim is not to restrict inclusion based on a specific TB diagnostic modality but to ensure that participants included in studies have been clearly identified as having TB infection in accordance with the criteria used by the study authors.*

**It seems to me that the concept of the scoping review is "public health impact of diagnostic and therapeutic interventions for depression in all forms of TB". Accordingly, the authors would also like to specify here that they are planning to include any study design that could help assess the public health impact of diagnostic and therapeutic interventions for depression in TB patients. Along with a comment of Reviewer 2 and scoping review guidelines, this scoping review should include all available evidence, regardless of of the publication language, especially since TB is highly prevalent in many countries where English is not among the official languages.**

*Thank you for this insightful comment. We would like to clarify that we will not be able to determine the public health impact of diagnostic and therapeutic interventions for depression in PWTB as we are not proposing to conduct a meta-analysis. Rather, we are focused on establishing the current state of the literature and highlight methodological gaps that exist in the study of these two co-morbid conditions.*

*Regarding language restrictions, while we recognize the importance of including non-English language publications—particularly given the global burden of TB—we must balance this with the capacity of our research team. Therefore, we regrettably must restrict the search to English language studies as we lack funding for translations and do not have at least 2 team members who speak additional languages beyond English. We will document and transparently report any exclusions based on language to acknowledge this as a potential limitation. This approach aims to maximize the inclusiveness of the review while remaining feasible within available resources.*

**I suggest splitting this arrow into two arrows: one on study setting and another one on the individuals who assessed the public health impact of diagnostic and therapeutic interventions for depression in TB patients. In the study setting arrow, the authors should report on i) the continental coverage of studies to be included, ii) countries where studies to be included should have been conducted, iii) study localities, i.e., urban, semi-urban, and rural; and housing platforms where included studies were conducted: community centres vs hospital centres, unspecialized versus specialized (TB centres versus mental health/depression centres versus TB-mental health centres [e.g., Jamot Hospital in Yaounde-Cameroon]) centres, and public health centres versus centres for purposes other than public health promotion. The second arrow would report on whether you will include studies regardless of credentials of individuals who evaluated the public health impact of diagnostic and therapeutic interventions for depression in individuals with TB: public health practioners versus others.**

*Thank you for this suggestion. We have re-worked Table 1 based on Reviewer #1’s suggestions and believe that legibility and clarity has been improved. Many of the suggestions you make here are in our data extraction template for study details and we will be sure to report on them for our findings. Here we are wanting to make sure that the reader understands that the context is largely not determining the inclusion in this scoping review as long as the study has taken place within humans (i.e. not veterinary settings and not laboratory only settings)*

**This statement should come just after the statement on the searched databases in lines 109 to 113.**

**Could you try adding the forms of TB in preliminary search strategies and see whether this optimizes the sensitivity and specificity of search strategies?**

**PMID: 30271302 PMCID: PMC6148622 DOI: 10.5195/jmla.2018.283**

**McGowan et al. Journal of Clinical Epidemiology 75 (2016) 40e46**

**Duko et al. Ann Gen Psychiatry (2020) 19:30**

**https://doi.org/10.1186/s12991-020-00281-8**

**Consider mentioning the search strategies in tables in the main manuscript. The search strategy is a core part of a scoping review protocol article.**

*Thank you for suggesting this. Per your suggestion, we have now moved the referred to sentence to right after our discussion of databases (Lines 202-216).*

*Since we are using MESH terms and terminology suggested by the respective databases, we have captured all various forms of tuberculosis in the current search strategy for each database. Our team librarian (LR) ran many test searches prior to arriving on this particular strategy to ensure all types of TB would be included and all possible articles discussing depression and TB co-occurrence would be included as well.*

*While we agree that the search strategies are a core part of the scoping review protocol, we also recognize that they do not translate well to tables/figures. We would prefer to keep it as a supplemental document to ensure the appropriate formatting for each individual database is maintained for those readers who are interested in replicating or updating our results.*

**Would you mind replacing this phrase with "immunosuppressive status" to also consider other causes of immunosuppression?**

*Thank you for this thoughtful suggestion. While we understand the rationale for broadening the HIV phrasing to "immunosuppressive status," we have chosen to retain the specific reference to HIV in this context. Given the well-documented and disproportionately high prevalence of HIV co-infection among people with tuberculosis, as well as its significant implications for both TB progression and mental health outcomes, HIV remains the most relevant and commonly reported immunosuppressive condition in TB research. As such, the research team believes that specifying HIV is both appropriate and necessary to accurately reflect the epidemiological and clinical realities in this population. We will not however limit our data extraction to only HIV related immunosuppressive states if a study does report on it. We just believe this is the most relevant to provide as an example.*

**Consider adding socioeconomic and nutritional status which are also important social determinants of TB.**

*Thank you. We have now added in.*

**Can you specify the theme analysis frameworks used in qualitative studies in brackets?**

**Medwave. 2024 Nov 22;24(10):e2972. doi: 10.5867/medwave.2024.10.2972.**

*We have now added in “*(i.e. thematic analysis, grounded theory, case study approach, etc.)” *(lins 268-269).*

**I see "narrative synthesis" nowhere in this paragraph despite this form of data synthesis being the main one used in the scoping review design.**

*Thank you for this comment. We recognize that narrative synthesis is commonly used in scoping reviews; however, for this study, we have intentionally chosen not to adopt a narrative synthesis approach. Instead, we will apply a structured framework-based synthesis using the RE-AIM framework to enhance methodological transparency and rigor. This approach allows us to systematically categorize and analyze the methodological characteristics of included studies in relation to public health impact, rather than relying on a primarily descriptive or thematic narrative. We believe this framework-guided strategy will provide greater clarity and utility for guiding future research and informing intervention development.*

**Can you specify the risk of bias assessment tools you plan to use?**

*Certainly! We have now added in the following sentence (Line 281-284):*

As there will be many different study designs, to assess quality, we will use the Jadad scale for randomized controlled trials, the Newcastle-Ottawa scale for observational studies, and the Joanna Briggs Institute Critical Appraisal Tool for Qualitative Research for qualitative studies

**Will it not be more precise to say that you will assess the public health impact of diagnostic and therapeutic interventions for depression using the RE-AIM framework?**

*We will not be assessing the public health impact in this scoping review. We will be characterizing the state of existing literature. Assessing the public health impact could be done in a follow-up systematic review and meta-analysis depending on the findings of the current scoping review.*

**I suggest revising the text in lines 166-192 to clearly specify how the public health impact of depression diagnostic and therapeutic interventions will be assessed through the RE-AIM model ((Am J Public Health**

**. 1999 Sep;89(9):1322-7. doi: 10.2105/ajph.89.9.1322.). The revised text could read:**

**"R for Reach, refers to the evaluation of how well depression diagnostic and therapeutic interventions were delivered to the target populations/high-risk TB groups.**

**E for efficacy, refers to the assessment of the accuracy (sensitivity, specificity, AUC , etc) of depression diagnostic methods, efficacy of depression treatment methods, as well as cost-benefit, cost-utility and cost-effectiveness (Iran J Public Health**

**. 2022 Oct;51(10):2159-2170. doi: 10.18502/ijph.v51i10.10975.) of depression diagnostic and treatment methods in individuals with different forms of tuberculosis. A for adoption, refers to proportions of the different individuals or legal/regulatory bodies that adopted depression diagnostic and treatment methods as well as barriers and facilitators to the adoption of depression diagnostic and intervention methods in TB patients. I for implementation, refers to... M for mainetnance, refers to...**

*Thank you for your thoughtful suggestion. We appreciate the recommendation to align our use of the RE-AIM framework with more traditional applications that focus on evaluating the public health impact of interventions. However, we would like to clarify that this scoping review is not designed to evaluate the effectiveness or real-world impact of depression diagnostic or therapeutic interventions among individuals with tuberculosis. Rather, the purpose of this review is to* ***map and characterize the methodologies*** *used in the existing literature—including how depression is studied, diagnosed, and treated in TB populations—and to identify* ***knowledge gaps*** *that may inform future research and implementation efforts.*

*As such, we are applying the RE-AIM framework not to assess intervention outcomes per se, but to organize and interpret how existing studies reflect various dimensions relevant to public health research, such as who is being studied (****Reach****), what methodological designs are being used (****Effectiveness****, as a proxy), where and by whom depression-related methods are applied (****Adoption****), what contextual barriers and facilitators are reported (****Implementation****), and whether there is any evidence of longer-term integration (****Maintenance****). This use of RE-AIM aligns with its broader utility as a planning and evaluative framework that can guide the interpretation of complex evidence across multiple settings, even in the absence of formal impact evaluation (Glasgow et al., 1999).*

**Consider addressing the "Cosnultation with stakeholders" sub-section of the O'Malley and Arksey framework just before this sub-section.**

**This sub-title should be in bold because it points to a sub-section.**

**Consider adding four sub-sections after this one and before the discussion section:**

**-Patient and public involvement (if you include patients/patient partners, how will you select them?)**

**-Dissemination and ethics**

**-Data sharing (in line with Reviewer 1's comment)**

**-Protocol deviation (is it possible that the protocol be modified even after the publication of this article? how will you deal with that issue in case it happens?)?**

*Rather than include both a section on patient/public involvement and a section on consultation with stakeholders, we have added in a section on Consultation with Stakeholders that explicitly states that we will draw on a diverse group of stakeholders, including PWTB currently participating in an ongoing and unrelated cohort study in Uganda to ensure that all conclusions and interpretations that are made as part of the scoping review are in line with lived experiences of relevant stakeholders. Given our teams extensive network of TB professionals across many continents, we believe we will be able to make meaningful conclusions about our work by drawing on the various experts within these networks. Please see the newly created section in Lines 316-326.*

*As mentioned below in our response to reviewer 1, we have added in Data Sharing (lines 327-330). We believe this covers the dissemination portion you mention above. We do not anticipate any protocol deviations as the protocol is considered “locked” and all team members have been involved in the process of creating this protocol.*

**Can you revise this paragraph specifying the strengths of the methods planned to be used in the future scoping review?**

*Certainly. While we believe the paragraph in question highlights many of the strengths for our review’s aims, we have now added in some additional strengths on the methodological rigor we plan to employ (lines 353-361):*

Additionally, our review will be methodologically rigorous by using the Levac et al framework to ensure a systematic and transparent approach to study identification and selection. This will be enhanced through the use of RE-AIM to synthesize results, providing a structured lens to assess how methodological approaches to researching TB and depression have created knowledge gaps in studying the diseases concurrently. Finally, the planned stakeholder consultations will add depth and practical relevance to the final interpretation of the findings ensuring that the review will be both methodologically rigorous and highly relevant to global TB and mental health research practices.

**The review has not yet been conducted. Would you mind revising this statement delineating the anticipated limitations of the methods planned to be used in the future scoping review? Focus should be made mainly on depression case-identification methods, limitations of the methods planned to be used for study identification, and anticipated limitations of RE-AIM assessment of the public health impact of depression diagnostic and therapeutic methods in individuals with different forms of TB.**

*As noted in the timeline section, we have conducted the initial search (on March 31, 2025). We are currently screening titles and abstracts now. Based on your comments and those of Reviewer 1, we have updated the limitations (lines 372-383). As noted above, determining the public health impact is beyond the scope of this review and is a question better suited for a subsequent systematic review and meta-analysis. Please see lines 372-3 for the newly added limitations about scoping review methodologies.*

Third, since the primary aim is to describe and categorize existing research rather than synthesize findings or estimate effect sizes, scoping reviews cannot make definitive conclusions about intervention effectiveness or causality. However, depending on the results of the current review, it may be appropriate to conduct a subsequent systematic review and meta-analysis to assess the public health impact of depression-related interventions amongst PWTB. Finally, as with all scoping reviews, the breadth of study types and topics can make data synthesis more complex than in a systematic review. Given our aim of establishing methodologies used in the field, a broad inclusion is necessary but could provide more narrative results. We believe our use of RE-AIM to structure the results, regardless of study type, will aid in creating meaningful outputs.

**This should also be revised to strengthen it consistently with the aim of the planned scoping review.**

*We have re-worded the sentence in question as follows (lines 386-388):*

This scoping review will advance understanding of how depression has been studied in the context of tuberculosis by systematically mapping existing methodologies and identifying critical research gaps to guide future public health research and intervention development.

**Reviewer #1: Review for: “Methodologies for Studying Depression in Persons living with Tuberculosis: Protocol
for a Scoping Review“

The paper at hand is the study protocol for a scoping review about methodologies for studying depression in persons suffering from tuberculosis. The paper is generally well written. The authors refer to established guidelines of conducting scoping reviews. The inclusion of supporting information like the PRISMA checklist or the search terms is laudable. The high prevalence of depression in tuberculosis and the detrimental consequences of both conditions makes this a very important scientific endeavor. In my view this work shows great promise and I applaud the authors for their work. Nevertheless, I have some concerns and comments that I feel need to be addressed. Please find my detailed review below.**

**My main point concerns the description of the aim of the planned scoping review. The methods section mostly seem to indicate that the aim is to find treatment trials, i.e., trials in which either depression and/or tuberculosis are treated. In the results section you convey that you will consider diverse study types. This seems good because treatment studies are not the only way to find out more about any associations between the two diseases. In any case, after the revision, the paper should give some examples for typical types of studies that you aim to include. I get that you cannot anticipate every type of study that explores the interrelationships between tuberculosis and depression but at the moment it is hard to grasp how do you aim to get relevant information on comorbidity and treatment options and whether interventional studies are the backbone of your review or one source of many. I don't think there is necessarily a problem with the content here, but rather difficulties arise due to the manner in which the research plan and the narrative flow of the text are presented.**

*Thank you for your thoughtful review and comments. We believe that we have updated the methods section to better reflect the fact that we will be going far beyond treatment studies for the present review but rather capturing any study that is aiming to characterize the relationship between TB and depression. We believe that our re-design of Table 1 (based on one of your later comments) in particular now makes it quite clear that we are aiming for a broad range of studies in order to characterize methodological gaps that exist in the study of comorbid depression and TB. We hope that our edits have made the narrative flow more clear to ensure readers understand the aim of our review.*  **The second major point that strikes me is the theoretical embedding of the connections between tuberculosis and depression. It is quite well deduced that it exists and that it should be investigated further. However, it does not go into what previous findings have shown as to why this connection exists. I am not an expert in this field. However, I wonder what psychological and/or biological factors could be interacting here? Is there relevant biopsychological literature regarding the interrelationship? What about the neuroscience of tuberculosis?

I also wonder whether meta-theories such as the “complex systems” perspective (which I am more familiar with) could be utilized to explain the relationships?**

**Fried, E. I., & Robinaugh, D. J. (2020). Systems all the way down: embracing complexity in mental health research. BMC medicine, 18, 1-4.

Scheffer, M., Bockting, C. L., Borsboom, D., Cools, R., Delecroix, C., Hartmann, J. A., ... & Nelson, B. (2024). A dynamical systems view of psychiatric disorders—theory: a review. JAMA psychiatry, 81(6), 618-623.

Westhoff, M., Berg, M., Reif, A., Rief, W., & Hofmann, S. G. (2024). Major problems in clinical psychological science and how to address them. Introducing a multimodal dynamical network approach. Cognitive Therapy and Research, 48(5), 791-807.**

*Thank you for highlighting the complexity of TB and depression co-occurrence. It is clear that you carefully reviewed our manuscript with the intention of improving our thinking and approach and we are deeply appreciative of your careful consideration and advice.*

*We have updated our introduction extensively and have included examples of the biological and psychological interactions (detailed more below). We have also taken your suggestion about highlighting complex systems thinking and have used many of the references you suggested to highlight the utility in using systems thinking as an approach to TB and depression. Please see the newly added sentences about systems thinking (lines 142-163):*

Given this, a complex systems thinking approach (37-39) may be of use to researchers as it highlights the importance of moving beyond isolated risk factors to better consider the dynamic, bidirectional, and context-dependent interactions between TB and depression. This perspective also underscores the need for diverse and multi-level methodological approaches that can better capture feedback loops (e.g. how depression worsens TB outcomes, which may in turn exacerbate depressive symptoms), variability across TB subtypes, and health system contexts. Furthermore, applying such a lens can help identify critical gaps in the current research landscape and more effectively guide future investigative efforts.

**More importantly, there seem to be reviews that discuss possible reasons for mental health problems in tuberculosis patients that you could cite (or discuss in more depth).
Cite:

Alene, K. A., Wangdi, K., Colquhoun, S., Chani, K., Islam, T., Rahevar, K., ... & Viney, K. (2021). Tuberculosis related disability: a systematic review and meta-analysis. BMC medicine, 19, 1-19.
Discuss in more depth:

Hayward, S. E., Deal, A., Rustage, K., Nellums, L. B., Sweetland, A. C., Boccia, D., ... & Friedland, J. S. (2022). The relationship between mental health and risk of active tuberculosis: a systematic review. BMJ open, 12(1), e048945.

Cáceres, G., Calderon, R., & Ugarte-Gil, C. (2022). Tuberculosis and comorbidities: treatment challenges in patients with comorbid diabetes mellitus and depression. Therapeutic Advances in Infectious Disease, 9, 20499361221095831.

I get that an extensive discussion might be to much for a protocol paper but some hints into the possible nature of the relationships should be given and be it on a meta (e.g., systems) level. For a protocol paper, I think that one to two paragraphs are enough but the reader will need some context information here.**

*We have now added additional details and examples into the introduction about the intersecting biological, social and structural factors that can increase the risk or severity of depression for those living with TB. We have also added in numerous references, including many that you suggest here. Please see the entire introduction which has been extensively re-worked or this excerpt here which focuses on the mechanisms underlying depression and tuberculosis (lines 104-126):*

Depression may not only be a consequence of TB, but also a potential risk factor due to its effects on immune function, health-seeking behavior, and treatment engagement (21, 22). Furthermore, TB-depression co-occurrence can be thought of as a syndemic, or an interacting set of conditions that cluster within social disadvantage, including intersecting biological, social, and structural factors, that results in an increased burden of disease (23-25). For example, HIV infection, a highly prevalent co-morbidity in PWTB, contributes to immunosuppression, social stigma, and economic vulnerability, all of which heighten susceptibility to mental health disorders (20). Additionally, the burden of depression may also vary by TB subtype —individuals with extrapulmonary TB may face diagnostic delays and uncertainty that heighten psychological distress (26, 27), while those with multidrug-resistant TB (MDR-TB) often endure prolonged treatment, more severe side effects, greater economic uncertainty, and greater social isolation, all of which may increase the risk or severity of depression (28, 29)

**Finally, the protocol should have a statement about open science practices. Will the extraction sheet be made openly available? How do you plan to share important study metadata? If you aim to share your data, at which repository do you want to share it? If you do not aim to share your data, this deserves justification in my view.**

*We have now added the following sentence (lines 327-330):*

In line with open science principles, we will make the final data extraction sheet and study metadata openly available through a public repository such as the Open Science Framework upon completion of the review.

**Minor**

**Sometimes you seem to use [] for indicating your sources and sometimes you use (). Please stick to a standardized style of reference indicators.**

*Thank you. We have now ensured that our citations fully comply with PLOS ONE guidelines.*

**The list of references is quite short at the moment. I am aware that this is a protocol paper but I would think that the paper would benefit from additional sources.**

*We have now greatly added to the overall number of references based on your suggestions as well as those of the other reviewers. Thank you for this suggestion as well as specific suggestions of papers to cite which we believe have greatly enhanced the rigor of our manuscript.*

**Line 55: “is one of the leading infectious killers worldwide”: I would recommend against using “killer” and instead use words like lethality / mortality here. Furthermore, I would recommend you cite relevant literature to support this (true) claim.**

*We have altered the phrasing to now say “leading infectious causes of mortality” and cite the Global Tuberculosis Report 2024.*

**Line 74: Please remove the “staggering”.**

*We have removed “staggering” as you suggest.*

**Table 1 contains long sentences and is really difficult to read. I think it needs substantial rework. I would recommend that you shorten the text, consider using bullet points instead of sentences and consider using spacing options to make your points clearer.**

*We have reformatted Table 1 with bullet points as you suggest as well as removed some wording. We believe this has improved the legibility of the table.*

**Line 115: Good that you screen the references of the included papers. Consider including relevant literature for the benefits
of footnote chasing.**

*Thank you for this suggestion. We agree that citation chasing can be a valuable method for identifying additional relevant studies. However, given the scope of our initial database search, which returned over 15,000 citations, and the resource constraints of our research team, we have opted to limit citation chasing to the references of included studies only, as originally planned. We believe this approach balances comprehensiveness with feasibility and aligns with the methodological standards for scoping reviews.*

**Limitations section: I would recommend that you critically discuss the strength and shortcomings of scoping reviews compared to traditional systematic reviews and meta-analyses and justify your choice for the scoping review. What I like about the limitations section is the critical discussion of cultural aspects.**

*We have added in additional limitations based on the general study design as follows (lines 372-383):*

Third, since the primary aim is to describe and categorize existing research rather than synthesize findings or estimate effect sizes, scoping reviews cannot make definitive conclusions about intervention effectiveness or causality. However, depending on the results of the current review, it may be appropriate to conduct a subsequent systematic review and meta-analysis to assess the public health impact of depression-related interventions amongst PWTB. Finally, as with all scoping reviews, the breadth of study types and topics can make data synthesis more complex than in a systematic review. Given our aim of establishing methodologies used in the field, a broad inclusion is necessary but could provide more narrative results. We believe our use of RE-AIM to structure the results, regardless of study type, will aid in creating meaningful outputs.

**Line 219: “Our review does have”: Maybe write: “Our review will have some limitations that are worth pointing out a priori”... Right now it sounds as if the review was already conducted. Down below in the conclusions section you use “will” and I would recommend also using it here.**

*We have adopted the language you propose here (Line 363).*  **I support an open and transparent review process. Therefore, I do not wish to stay anonymous.
Thanks to the authors and to the journal editors for giving me the opportunity to review this paper.

Max Berg,

University of Marburg
Clinical Psychology Group**

*Again, we would like to thank you for your thoughtful and constructive review. We believe it has enhanced our protocol manuscript and we are grateful that you participated in this peer review process.*

**Reviewer #2: The article is a study protocol for a scoping review aimed at mapping methodologies used to study depression in individuals with tuberculosis (TB). The primary objective is to identify gaps in research design, diagnostic tools, and treatment strategies for depression in TB patients. The authors highlight the co-occurrence of TB and depression and the need for robust research approaches to improve integrated care. The protocol outlines a systematic approach to reviewing existing literature, with the goal of guiding future research and public health interventions.

Methodology Evaluation:
The study employs the Arksey and O’Malley framework, updated by Levac et al., for conducting scoping reviews, which is appropriate for mapping broad research areas. The protocol includes six steps: identifying the research question, selecting relevant studies, charting data, collating results, and consulting stakeholders. The search strategy is comprehensive, covering databases like MEDLINE, Embase, PsycINFO, and Africa-Wide Information, ensuring a global perspective. Inclusion criteria are well-defined, focusing on studies that examine the TB-depression relationship, while exclusion criteria eliminate irrelevant or low-quality studies.**

*Thank you for this summary and highlighting some of the strengths of our approach!*  **Data extraction will be performed using Covidence, with two reviewers screening abstracts and full texts to minimize bias. The RE-AIM framework (Reach, Effectiveness, Adoption, Implementation, Maintenance) will structure the analysis, which is a strength for evaluating the impact of research methodologies. However, the protocol limits the search to English-language studies, potentially excluding relevant research from high-TB-burden, non-English-speaking countries. Additionally, the reliance on Western terms like "depression" may overlook culturally specific expressions of mental health conditions.**

*As mentioned above, regarding language restrictions, while we recognize the importance of including non-English language publications—particularly given the global burden of TB—we must balance this with the capacity of our research team. Therefore, we regrettably must restrict the search to English language studies as we lack funding for translations and do not have at least 2 team members who speak additional languages beyond English. We will document and transparently report any exclusions based on language to acknowledge this as a potential limitation. This approach aims to maximize the inclusiveness of the review while remaining feasible within available resources.*

*You are right to highlight the reliance on Western terms and we were sure to specify it as a key limitation in our discussion section. After running test searches, we expanded our criteria to include broad keywords such as “Mental Health”, “Mood Disorder”, “Mental Disorder” and other similar words (please see S1 Appendix). We determined that these types of keywords resulted in studies from all continents and not just Western countries. We do however know that it is a limitation of our approach and why we have reported it here (lines 364-369), and will continue to report it in our subsequent findings.*  **Strengths and Weaknesses:
Strengths:

Comprehensive search strategy across multiple databases.

Clear inclusion/exclusion criteria to ensure relevance.

Use of the RE-AIM framework to evaluate methodological impact.

Dual-reviewer process to reduce bias in study selection.

Weaknesses:

Language restriction (English-only) may exclude significant studies.

Potential cultural bias in defining depression, missing non-Western perspectives.**

*Thank you again for highlighting these weaknesses. Please see above our response to these weaknesses.*  **No pilot data or preliminary results to validate the approach.**

*We did conduct piloting of the search strategy over several weeks and agree it was an oversight on our part to not make that clear in the text. We have now added in sentences (Lines 202 – 217) to make that process clear.*

The preliminary search strategy, constructed by a trained medical librarian (LR) after several weeks of testing various combinations of terms, focused on combinations of the following areas: terms related to “tuberculosis” and terms related to “depression”.

**Limited discussion of how heterogeneity in study designs will be managed during synthesis.**

*We have now added in the following to highlight the different ways we will assess study quality by study design (Lines 281-284):*

As there will be many different study designs, to assess quality, we will use the Jadad scale for randomized controlled trials, the Newcastle-Ottawa scale for observational studies, and the Joanna Briggs Institute Critical Appraisal Tool for Qualitative Research for qualitative studies.

*Additionally, we propose using RE-AIM, which can be used for any study design type, to assist in creating coherence across many contexts, study designs, and study populations. Finally, we have explicitly added into the limitations section a discussion on the difficulties that heterogeneous study designs can present (lines 377-383).*

Finally, as with all scoping reviews, the breadth of study types and topics can make data synthesis more complex than in a systematic review. Given our aim of establishing methodologies used in the field, a broad inclusion is necessary but could provide more narrative results. We believe our use of RE-AIM to structure the results, regardless of study type, will aid in creating meaningful outputs.

**Conclusion and Recommendations:
This protocol provides a rigorous framework for a scoping review that could significantly advance understanding of depression in TB patients. However, the exclusion of non-English studies and culturally specific terminology may limit its comprehensiveness. Future research should:

Expand language inclusion to capture studies from high-TB-burden regions.**

*While we hope that we have captured most of the relevant literature, this unfortunately remains a limitation given our study team and resource availability.*  **Incorporate culturally adapted definitions of depression.**

*As mentioned above, we have now made it clear that the search critera were developed after testing numerous combinations to maximize the number of terms captured. However, we will continue to point out of difficult it can be to capture all possible terms used for depression, especially outside the Western context.*  **Pilot the methodology to refine search terms and data extraction processes.**

*We have now explicitly stated that the search was created after weeks of piloting the process. We have now also specified that the data extraction tool will be piloted prior to implementation (line 250).*  **Address heterogeneity in study designs during data synthesis.**

*Please see our response above.*  **Overall, the protocol is well-structured and promises valuable insights, but its impact could be enhanced by addressing these limitations.**

*Thank you again for your thoughtful comments. They have helped to improve our manuscript and we are deeply appreciative that you have participated in the peer-review process.*
