# Supplementary material for: Methodologies for studying depression in persons living with tuberculosis: Protocol for a scoping review
Source: PLoS One. 2025 Jul 21;20(7):e0322753. doi: 10.1371/journal.pone.0322753 (PMC12279151; doi:10.1371/journal.pone.0322753)
Supplement: Attachment — Submitted filename: Response_to_Reviewers_auresp_2.docx [file pone.0322753.s006.docx]

*We would like to once again thank the editor and reviewer for careful consideration of our manuscript. Many thanks to Dr. Max Berg for his approval of the current version. We believe we have now addressed the concerns of Dr. Essouma as well. We have outlined changes and responses below.*

*To Dr. Essouma:
Thank you for your detailed feedback. For ease of incorporating revisions and ensuring we don’t overlook any of your comments, would you mind providing any additional feedback after this version using a Word document and citing line numbers rather than a PDF? We greatly appreciate your time and suggestions, and working in Word will help us respond more efficiently and accurately.*

“You tend to make long sentences. Think about reversing this tendency by rather increasing the number of simple sentences in the manuscript. Consider also being consistent with the use of abbreviations, not to mention the need for full spelling of abbreviations when first using them.
'etc' is not warranted in the manuscript."

*We have gone through and done as you suggest by shortening sentences and removed the use of ‘etc’.*

*Also to note, there were many spots where you have suggested certain wording. We have updated per your wording suggestions in the abstract and throughout the manuscript.*

Where are the keywords?

*In the documentation you had previously suppled about formatting per PLoS ONE guidelines, the keywords were not included within the manuscript itself but simply in the manuscript data fields. In this case the keywords are: tuberculosis; depression; global health; reviews. They can be found on the first page of the pdf in the six row of the table.*

This introduction needs to be shortened by eliminating redundant statements for example. This will help put more emphasis on the methods section which is the most important section of a protocol article such as this one. Even the reference count of the introduction would better be reduced, notably the number of references addressing the relationship between depression and TB. Consider keeping only the most recent highest-quality data synthesis articles or primary study articles where data synthesis articles are not available.

*We had previously expanded the introduction per the request of the reviewers. This included greatly expanding the reference count and included adding in references specifically requested by the reviewers. Given that this was an explicit ask of the reviewers, we would like to leave most of the added references in to maintain the approval of the reviewers. However, we have now shortened the introduction as you suggest and believe this puts more emphasis on the methods section.*

Consider supporting the statements on latent tuberculosis with at least one reference.

*We have now added in a reference at the designated location (Line 64).*

I still make this proposition to remove some statements below in order to reduce the length of the introduction.

*We have now shortened the introduction while trying to maintain the requested edits by the reviewers. We hope we have now struck a better balance between giving complete information while being succinct.*

This statement is implicitly repeated in lines 90-92. This is the reason why I propose this modification in lines 81-83.

*We have made the change as you proposed although have left the relevant references as they are both recent and relevant.*

Given that the sentence in lines 97-99 contains a bit of information provided in lines 77 and 78 and widespread knowledge that HIV infection naturally leads to immunosuppression, I suggest abbreviating this statement like this:

"TB-depression co-occurrence can be thought of as a syndemic, or an interacting set of both conditions that cluster within a constellation of biological (e.g., HIV infection) and social factors (e.g., stigma, economic vulnerability) factors capable of heightening susceptibility to mental health disorders."

I did not use the word "structural" because I thought "structural factors" may be embedded within "social factors".

*We have updated this sentence to the following (Lines 99-102) which we believe captures your intent while maintaining the emphasis on the definition of a syndemic:*

TB-depression co-occurrence can be thought of as a syndemic, or an interacting set of conditions that cluster within social disadvantage (e.g. stigmatization of health conditions and economic vulnerability) that results in an increased burden of disease (23-25).

Consider merging this paragraph with the previous one because the idea developed in this paragraph and that developed in the previous one are in the same vein and this will help decrease the length of the introduction.

*After much consideration, we have decided to maintain the current paragraph structure. This avoids having an extremely large paragraph and will improve readability. We thank you for the suggestion and as mentioned previously, we have worked to reduce the size of the introduction.*

I propose the deletion of this paragraph because this information has been provided in lines 70-74 though not using the exact same words.

*The paragraph in question specifies various mechanisms of the interaction between depression and tuberculosis. The lines that you reference here (lines 70-74) are specific to TB and do not touch on the interplay between the two diseases. Given this, we believe the readers will be expecting a simple synthesis of the known interactions between depression and tuberculosis so have chosen to leave this paragraph in. We have made of wording changes to further reduce the length of the introduction but believe this is an important section for synthesizing what has been previously stated.*

This information has already been provided above and is also provided in lines 284-287 though not using the exact same words. I moved a section of this text "appears to have been limited to prevalence surveys" to line 90. Therefore, consider deleting this statement here.

*Unfortunately, we are not entirely sure which lines you are referencing as 284-287 is in the discussion and lines 84-87 do not provide comparable information. We have however altered this paragraph to be more concise (lines 179-184):*

Despite the significant impact of depression in PWTB, research has largely been limited to prevalence surveys. Few studies have used longitudinal designs and even fewer explore the methodologies used to screen, diagnose, or treat depression in PWTB. This may be in part due to diagnostic challenges posed by overlapping symptoms, such as TB-related fatigue, weight loss, and general malaise, all of which can masquerade as signs of depression, leading to misdiagnoses or missed diagnoses.

I suggest also deleting this statement (lines 118-131) from the introduction because some bits of information provided here have already been provided above though not using the exact same words. Furthermore, comments about diagnostic challenges and the need for complex systems thinking and multiple perspectives in the diagnosis and management of depression in PWTB would better be moved to the discussion section.

*We originally added this into the introduction at the request of a reviewer. We have now however eliminated this discussion from the introduction.*

The verb tenses in the methods, discussion and conclusion sections should be consistent with the information from the timeline sub-section.

*We believe we have now updated tense use appropriately based on the timeline.*

Because based on the information in line 269, it seems that you have started searching the literature for the scoping review.

*You are correct. We have placed the search in the past tense now.*

MeSH are only for PubMed, not the other databases. I therefore suggest deleting this sentence and referring readers to S1 appendix for the full search strategies used in different electronic databases at the end of the previous sentence.

*Thank you for this comment. While it is correct that MeSH terms are specific to PubMed, we note that several other databases included in our search strategy—such as Cochrane Library and PsycINFO—use controlled vocabularies that are either derived from or aligned with MeSH terminology. However, to avoid any potential confusion, we have removed the sentence in question and now refer readers directly to S1 Appendix for the complete search strategies used across databases. We appreciate the opportunity to clarify this point.*

Which laboratory samples? Specify, remembering that you said you will include even only microbiologically confirmed TB.

*In this case, the exclusion criteria means we will not be included “bench” studies. So for example studies that are utilizing blood or sputum samples to test new diagnostics or therapeutics but do not involve a clinical component. Many of these studies are focused on immune interactions and cellular responses which are not the focus of the current scoping review. This is why we have specified studies that are only looking at laboratory samples and isolates. Any individual with TB diagnosed in any way will be included with most PWTB being diagnosed microbiologically.*

I would replace this term with "Main variables"

*This is about the word “Outcome” used in our Table 1. However, we would like leave it as “Outcome” as this is the standard terminology utilized for reviews (Population, Intervention, Comparator, Outcome, Timing, Setting; PICOTS table).*

Revise this sub-section with simple sentences. Accordingly, you could summarize the data to be collected in a table inserted in the main manuscript (Table 2). This would shorten this sub-section while making it more readable.

*We have done as you advise and switched the majority of the “Charting the Data” paragraph into a Table, now labeled Table 2: Data Extraction.*

You would better present overall data and segregated data (segregation by age category [infants, children, adolescents, adults, and older adults] sex, gender, race, ethnicity, geographic location [continents/world regions] to name a few) and you need to mention that information in this sub-section as well.

*We have added in the following (Lines 358-360):*

We will provide descriptive characteristics of all included studies using figures and tables to fully summarize the studies, stratifying where appropriate by key variables such as by age, TB type, geographic location and/or study design to name a few.

Given that this paragraph is very long and difficult to follow and considering the importance of well explaining how the RE-AIM approach will be used in your scoping review, I suggest explaining how you will use the RE-AIM approach in the scoping review using a figure or a table inserted in the main manuscript. Alternatively, consider subdividing this paragraph into two paragraph including simple sentences.

*We have now shortened this paragraph and split it into two paragraphs.*

Consider revising this statement with the discussion section to avoid repeating information available here in the discussion section

*We have now shortened the referred to sentences (lines 416-418) to avoid replication of later sentences:*

Consultations will offer contextual insights and help identify gaps or implementation issues not captured in the published literature, ensuring the findings are meaningful and actionable for high TB burden settings.

There is also a need to shorten the discussion section by removing redundancy. The revisions proposed in the discussion therefore aim to abbreviate its text while keeping its main content unchanged.

*We had similarly expanded the Discussion to address concerns of the reviewers. We appreciate your assistance in identifying areas where we can be more concise and believe we have also further cut down on its size.*

I suggest deleting this statement because it repeats the information provided in lines 278 and 279, and the information "provide insights into potential pathways for improving integrated care" is somewhat provided in lines 290 and 291 using different words.

*We have eliminated as you suggest.*

Given that this statement discusses an idea that is quite different from the idea discussed in the preceding sentence, I suggest you go to the line from here.

It seems to me that the authors are discussing the strengths of the upcoming scoping review here. Therefore, I suggest you go to the line here because this idea is quite different from the one discussed in the previous sentence. Furthermore, consider revising the beginning of this statement in these likes:

"Strengths of our review will include a methodological rigour based on

*We have started a new paragraph at the requested spot (Line 467) and adjusted the sentence as you requested.*

This paragraph would better be shortened.

*Thank you for this suggestion. This comment refers to the limitations paragraph. While we have implemented some of the suggested edits to improve clarity, we have retained much of the original content, as this section was specifically highlighted by both peer reviewers as a strength of the manuscript. We believe preserving this version maintains alignment with their positive feedback.*

The terms "diagnosis" and "treatment" appear nowhere in the conclusion?

I also notice that from the introduction to the conclusion, you focus on the fact that the scoping review will help you find research gaps, but will it not first help you find the "state of knowledge" on methodologies used to assess depression diagnosis and treatment before helping you find the gaps in knowledge? Consider addressing this issue throughout the manuscript.

*Thank you for this thoughtful observation. We agree that the primary aim of the scoping review is to first map the current state of knowledge regarding methodologies used to study depression diagnosis and treatment in individuals with TB, and then to identify key gaps. While this intent is reflected throughout the manuscript, we acknowledge that the conclusion could more explicitly echo this two-step purpose. We have revised the conclusion slightly to incorporate the terms "diagnosis" and "treatment" and to more clearly emphasize that the review will both summarize existing methodological approaches and highlight areas where further research is needed (Lines 540-543). We believe this clarification strengthens alignment across the manuscript.*

This scoping review will advance understanding of how depression screening, diagnosis, and treatment have been studied in the context of tuberculosis by systematically mapping existing methodologies and identifying critical research gaps to guide future public health research and intervention development.
